# Supplementary material for: Unsupervised encoding selection through ensemble pruning for biomedical classification
Source: BioData Min. 2023 Mar 16;16:10. doi: 10.1186/s13040-022-00317-7 (PMC10018861; doi:10.1186/s13040-022-00317-7)

List of encodings

Refer to Spänig *et al.* (2021) for more details (https://doi.org/10.1093/nargab/lqab039).

encoding	params_1	params_2	params_3	params_4
aac				
aaindex	QIAN880102; KUMS000103; QIAN880103; BUNA790103; GEOR030106; QIAN880101; BUNA790102; RACS820107; AURR980115; GEOR030103; KHAG800101; FINA910104; RACS820102; FASG760103; RICJ880104; ZIMJ680104; ROBB760111; AURR980118; WOLS870102; QIAN880117; VASM830101			
apaac	lambda	3; 1; 7; 4; 5; 8; 10; 6; 2; 9		
asa				
binary				
blomap				
blosum62				
cgr	res	10; 20; 200; 100	sf	0.8632713; 0.5
cksaagp	gap	3; 1; 7; 5; 4; 8; 6; 2; 9		
cksaap	gap	3; 1; 7; 5; 4; 8; 6; 2; 9		
ctdc				

encoding	params_1	params_2	params_3	params_4
ctdd				
ctdt				
ctriad				
dde				
delaunay	average; frequency; total; cartesian; number	product; instances; distance		
disorderb				
disorderc				
dist_freq	dn	100; 5; 50; 10; 20	dc	100; 5; 50; 10; 20
distance	distribution			
dpc				
eaac	window	3; 1; 7; 4; 5; 8; 10; 6; 2; 9		
egaac	window	3; 1; 7; 5; 4; 8; 6; 2		
electrostatic_hull		3; 12; 0; 6; 9		
fft	aaindex	QIAN880102; KUMS000103; QIAN880103; BUNA790103; GEOR030106; QIAN880101; BUNA790102; RACS820107; AURR980115; GEOR030103; KHAG800101; FINA910104; RACS820102; FASG760103; RICJ880104; ZIMJ680104; ROBB760111; AURR980118;		

encoding	params_1	params_2	params_3	params_4
		WOLS870102; QIAN880117; VASM830101		
fldpc	aaindex	QIAN880102; KUMS000103; QIAN880103; BUNA790103; GEOR030106; QIAN880101; BUNA790102; RACS820107; AURR980115; GEOR030103; KHAG800101; FINA910104; RACS820102; FASG760103; RICJ880104; ZIMJ680104; ROBB760111; AURR980118; WOLS870102; QIAN880117; VASM830101		
flgc	aaindex	QIAN880102; KUMS000103; QIAN880103; BUNA790103; GEOR030106; QIAN880101; BUNA790102; RACS820107; AURR980115; GEOR030103; KHAG800101; FINA910104; RACS820102; FASG760103; RICJ880104; ZIMJ680104; ROBB760111; AURR980118; WOLS870102;		

encoding	params_1	params_2	params_3	params_4
		QIAN880117; VASM830101		
gaac				
gdpc				
geary	nlag	3; 1; 7; 4; 5; 8; 10; 6; 2; 9		
gtpc				
ksctriad	gap	2; 1; 3; 4		
moran	nlag	3; 1; 7; 4; 5; 8; 10; 6; 2; 9		
ngram	a2; s2; s3; a3; e3; e2	200; 100; 1; 5; 50; 300; 20		
nmbroto	nlag	3; 1; 7; 4; 5; 8; 10; 6; 2; 9		
paac	lambda	3; 1; 7; 4; 5; 8; 10; 6; 2; 9		
qsar				
qsorder	nlag	3; 1; 7; 4; 5; 8; 10; 6; 2; 9		
socnumber	nlag	3; 1; 7; 4; 5; 8; 10; 6; 2; 9		
sseb				
ssec				
psekraac t1	st-lambda-correlation	rt-10	ktu-3	la-6
psekraac t10	st-lambda-correlation	rt-10	ktu-1	la-3
psekraac t11	st-g-gap	rt-9	ktu-1	la-3
psekraac t12	st-g-gap	rt-8	ktu-1	la-1
psekraac t13	st-g-gap	rt-20	ktu-2	la-2
psekraac t14	st-g-gap	rt-8	ktu-1	la-2
psekraac t15	st-lambda-correlation	rt-13	ktu-2	la-2

encoding	params_1	params_2	params_3	params_4
psekraac t16	st-lambda-correlation	rt-10	ktu-2	la-2
psekraac t2	st-g-gap	rt-8	ktu-1	la-2
psekraac t3A	st-g-gap	rt-13	ktu-2	la-3
psekraac t3B	st-g-gap	rt-8	ktu-1	la-1
psekraac t4	st-g-gap	rt-9	ktu-1	la-1
psekraac t5	st-g-gap	rt-10	ktu-1	la-3
psekraac t6A	st-lambda-correlation	rt-20	ktu-2	la-3
psekraac t6B	st-lambda-correlation	rt-5	ktu-2	la-2
psekraac t6C	st-g-gap	rt-5	ktu-2	la-2
psekraac t7	st-g-gap	rt-10	ktu-2	la-3
psekraac t8	st-lambda-correlation	rt-12	ktu-2	la-3
psekraac t9	st-g-gap	rt-6	ktu-3	la-3
ta				
tpc				
waac	aaindex	QIAN880102; KUMS000103; QIAN880103; BUNA790103; GEOR030106; QIAN880101; BUNA790102; RACS820107; AURR980115; GEOR030103; KHAG800101; FINA910104; RACS820102; FASG760103; RICJ880104; ZIMJ680104; ROBB760111; AURR980118; WOLS870102;		

encoding	params_1	params_2	params_3	params_4
		QIAN880117; VASM830101		
zscale				

Statistics

anova_summary_aov

	term	df	sumsq	meansq	statistic	p.value	experiment
**1**	model	3	20.336352	6.778784	819.022774	0.0	anova_summary_aov
**2**	Residuals	396	3.277563	0.008277	-	-	anova_summary_aov

anova_tukey_hsd

	term	contrast	null.value	estimate	conf.low	conf.high	adj.p.value	experiment
**1**	model	dt-bayes	0	-0.428714	-0.461908	-0.395520	0.000000	anova_tukey_hsd
**2**	model	lr-bayes	0	-0.544838	-0.578032	-0.511644	0.000000	anova_tukey_hsd
**3**	model	rf-bayes	0	-0.551538	-0.584732	-0.518344	0.000000	anova_tukey_hsd
**4**	model	lr-dt	0	-0.116124	-0.149318	-0.082930	0.000000	anova_tukey_hsd
**5**	model	rf-dt	0	-0.122824	-0.156018	-0.089630	0.000000	anova_tukey_hsd
**6**	model	rf-lr	0	-0.006700	-0.039894	0.026493	0.954068	anova_tukey_hsd

anova_error_summary_aov

	term	df	sumsq	meansq	statistic	p.value	experiment
**1**	model	4	886.723119	221.680780	64876.43218	0.0	anova_error_summary_aov
**2**	Residuals	500912	1711.600948	0.003417	-	-	anova_error_summary_aov

anova_error_tukey_hsd

	term	contrast	null.value	estimate	conf.low	conf.high	adj.p.value	experiment
**1**	model	dt-bayes	0	-0.045910	-0.046622	-0.045198	0	anova_error_tukey_hsd
**2**	model	lr-bayes	0	-0.066478	-0.067190	-0.065765	0	anova_error_tukey_hsd
**3**	model	mlp-bayes	0	-0.093076	-0.093789	-0.092364	0	anova_error_tukey_hsd
**4**	model	rf-bayes	0	-0.123956	-0.124669	-0.123244	0	anova_error_tukey_hsd
**5**	model	lr-dt	0	-0.020568	-0.021280	-0.019855	0	anova_error_tukey_hsd
**6**	model	mlp-dt	0	-0.047166	-0.047878	-0.046454	0	anova_error_tukey_hsd
**7**	model	rf-dt	0	-0.078046	-0.078759	-0.077334	0	anova_error_tukey_hsd
**8**	model	mlp-lr	0	-0.026598	-0.027311	-0.025886	0	anova_error_tukey_hsd

	term	contrast	null.value	estimate	conf.low	conf.high	adj.p.value	experiment
**9**	model	rf-lr	0	-0.057479	-0.058191	-0.056766	0	anova_error_tukey_hsd
**10**	model	rf-mlp	0	-0.030880	-0.031593	-0.030168	0	anova_error_tukey_hsd

anova_kappa_summary_aov

	term	df	sumsq	meansq	statistic	p.value	experiment
**1**	model	4	6356.266286	1589.066571	34525.541017	0.0	anova_kappa_summary_aov
**2**	Residuals	500912	23054.888961	0.046026	-	-	anova_kappa_summary_aov

anova_kappa_tukey_hsd

	term	contrast	null.value	estimate	conf.low	conf.high	adj.p.value	experiment
**1**	model	dt-bayes	0	0.064871	0.062256	0.067485	0	anova_kappa_tukey_hsd
**2**	model	lr-bayes	0	0.038168	0.035553	0.040782	0	anova_kappa_tukey_hsd
**3**	model	mlp-bayes	0	0.142361	0.139747	0.144976	0	anova_kappa_tukey_hsd
**4**	model	rf-bayes	0	0.317744	0.315129	0.320359	0	anova_kappa_tukey_hsd
**5**	model	lr-dt	0	-0.026703	-0.029317	-0.024089	0	anova_kappa_tukey_hsd
**6**	model	mlp-dt	0	0.077491	0.074876	0.080105	0	anova_kappa_tukey_hsd
**7**	model	rf-dt	0	0.252874	0.250259	0.255489	0	anova_kappa_tukey_hsd
**8**	model	mlp-lr	0	0.104194	0.101579	0.106808	0	anova_kappa_tukey_hsd
**9**	model	rf-lr	0	0.279577	0.276962	0.282192	0	anova_kappa_tukey_hsd
**10**	model	rf-mlp	0	0.175383	0.172768	0.177998	0	anova_kappa_tukey_hsd

manova_summary

	term	df	pillai	statistic	num.df	den.df	p.value	experiment
**1**	model	4	0.371843	30209.171131	8.0	1058194.0	0.0	manova_summary
**2**	Residuals	529097	-	-	-	-	-	manova_summary

manova_summary_aov

	Df	Sum.Sq	Mean.Sq	F.value	Pr..F.	response	experiment
**model**	4	6340.009140	1585.002285	30630.834702	0.0	Response 1	manova_summary_aov
**Residuals**	529097	27378.292564	0.051745	-	-	Response	manova_summar

	Df	Sum.Sq	Mean.Sq	F.value	Pr..F.	response	experiment
						1	y_aov
**model 1**	4	906.161403	226.540351	58151.351568	0.0	Response 2	manova_summary_aov
**Residuals 1**	529097	2061.204369	0.003896	-	-	Response 2	manova_summary_aov

Plots

Refer to main manuscript for more details.

Suppl. Fig. 1. MVO fitness vs. generations.

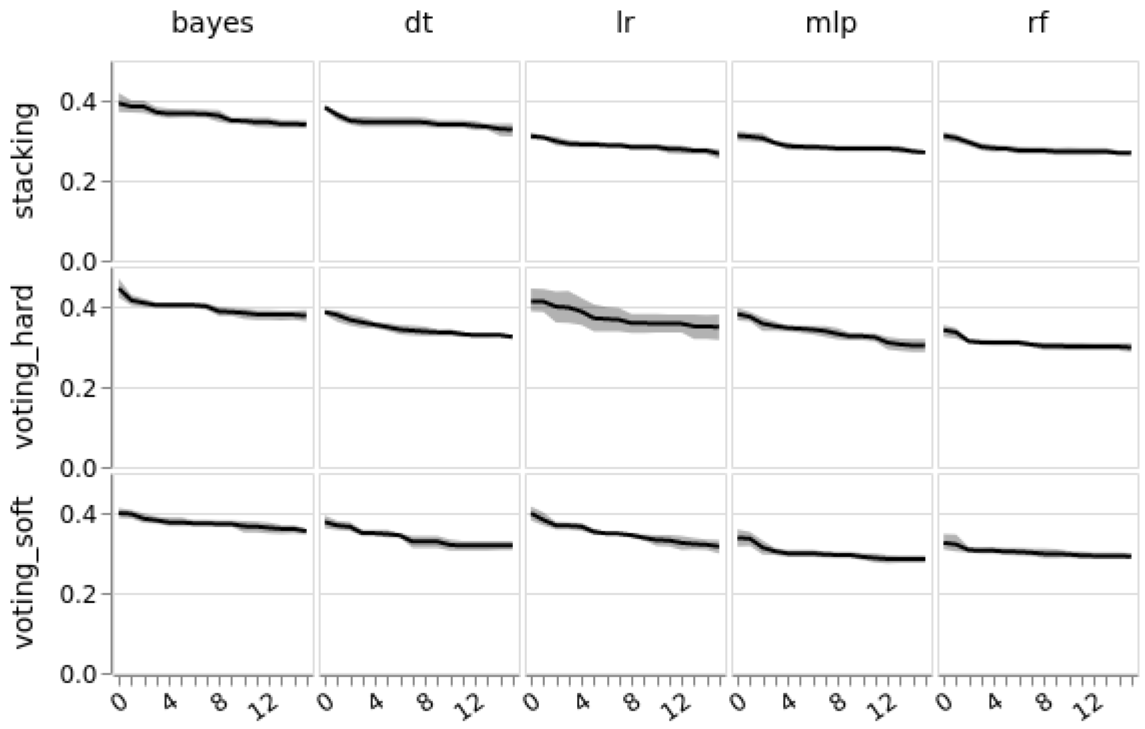

Suppl. Fig. 2. XCD chart

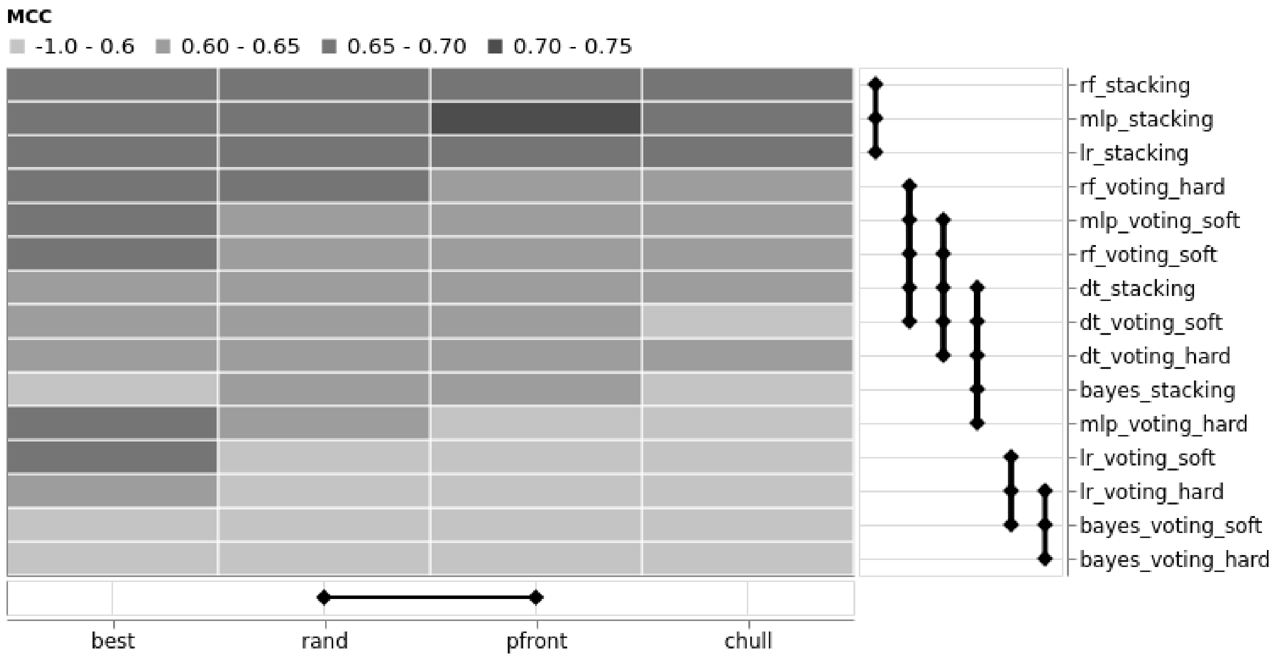

Suppl. Fig. 3. Boxplot

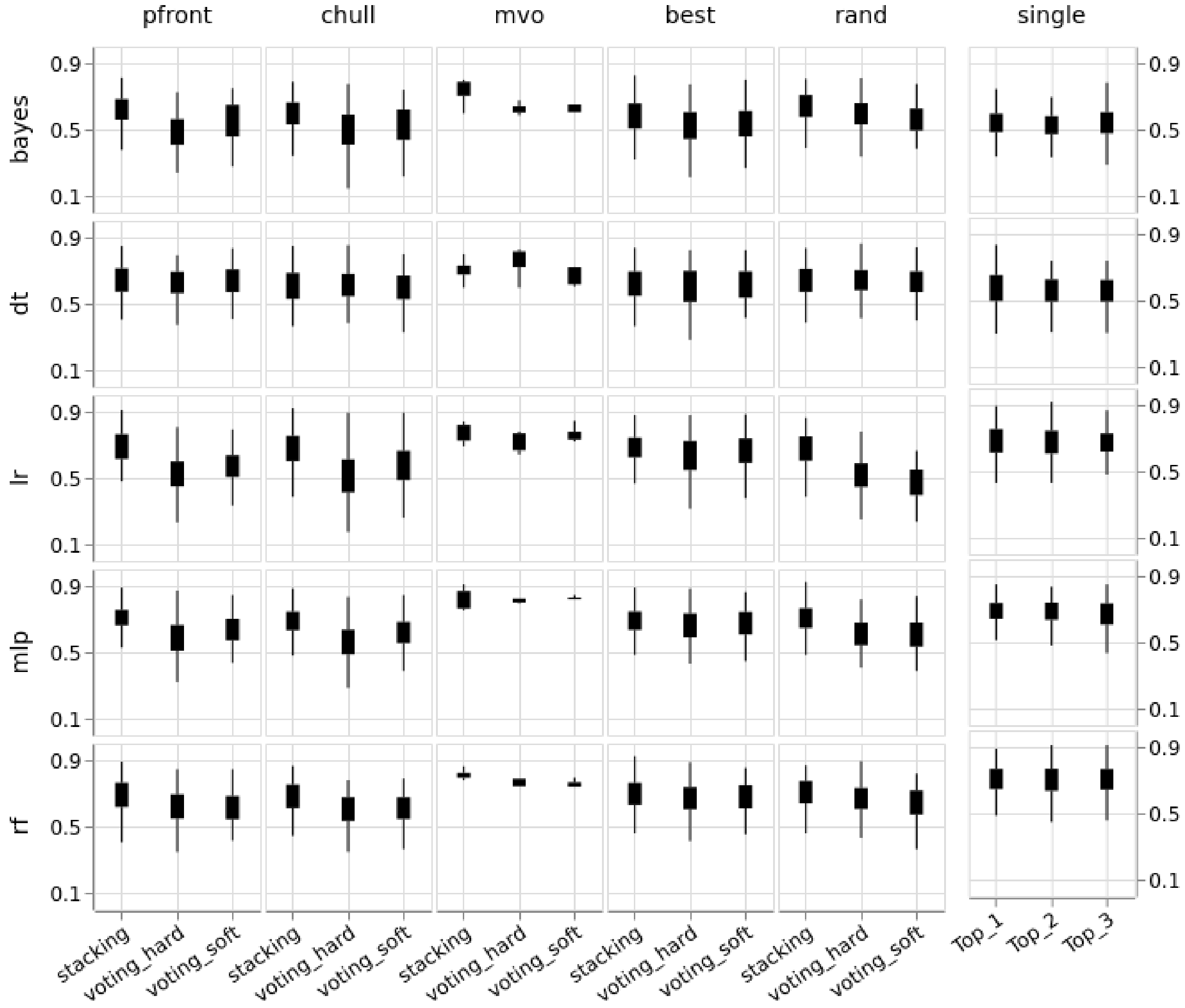

Suppl. Fig. 4. Kappa-error plot

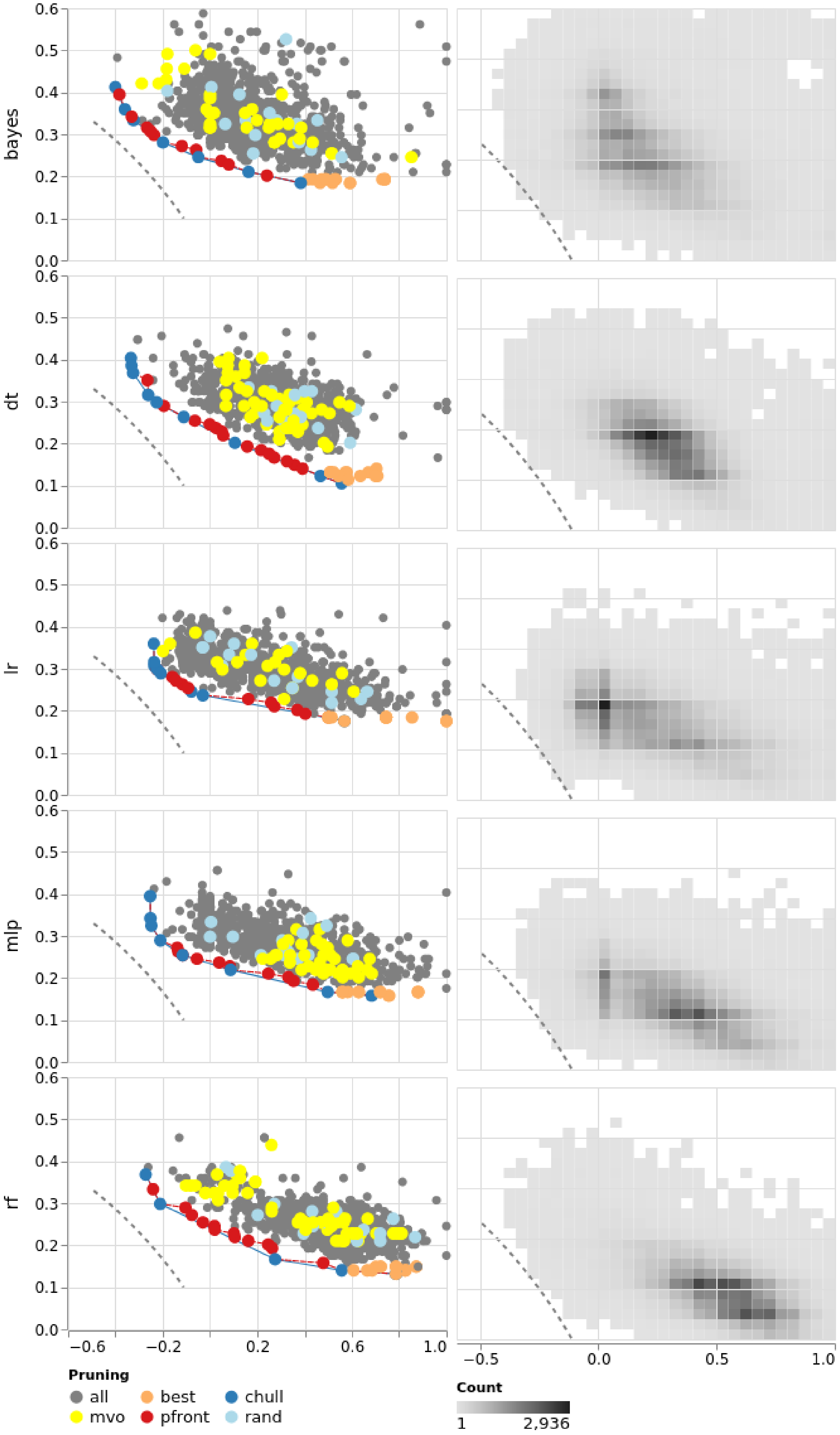
bayes
dt
lr
mlp
rf
0.0
0.1
0.2
0.3
0.4
0.5
0.6
−0.6
−0.2
0.2
0.6
1.0
−0.5
0.0
0.5
1.0
Pruning
all
best
chull
mvo
pfront
rand
Count
1
2,936

Suppl. Fig. 5. Boxplot MANOVA

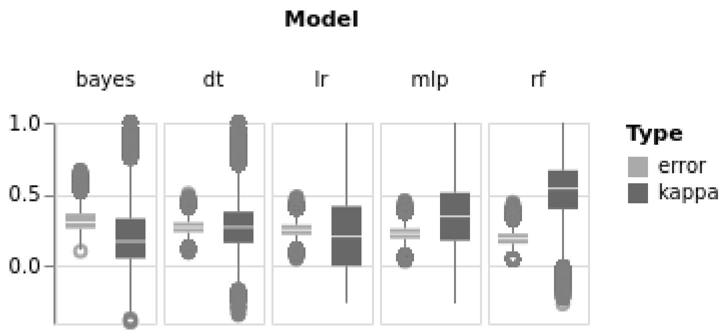

Supplement: Supplementary file 1 — Additional file 1. [file 13040_2022_317_MOESM1_ESM.zip › supplements/acp_mlacpR1.pdf]
