# Supplementary material for: Unsupervised encoding selection through ensemble pruning for biomedical classification
Source: BioData Min. 2023 Mar 16;16:10. doi: 10.1186/s13040-022-00317-7 (PMC10018861; doi:10.1186/s13040-022-00317-7)

List of encodings

Refer to Spänig *et al.* (2021) for more details (https://doi.org/10.1093/nargab/lqab039).

**encoding**	**params_1**	**params_2**	**params_3**	**params_4**
aac				
aaindex	WOLS870102; ROBB760111; RICJ880104; GEOR030106; FASG760103; ZIMJ680104; KHAG800101; VASM830101; QIAN880101; BUNA790103; AURR980115; QIAN880102; RACS820107; GEOR030103; BUNA790102; QIAN880103; AURR980118; RACS820102; KUMS000103; FINA910104; QIAN880117			
apaac	lambda	6; 4; 3; 1; 2; 5		
asa				
binary				
blomap				
blosum62				
cgr	res	20; 10; 100; 200	sf	0.8632713; 0.5
cksaagp	gap	4; 3; 1; 2; 5		
cksaap	gap	4; 3; 1; 2; 5		
ctdc				
ctdd				
ctdt				

**encoding**	**params_1**	**params_2**	**params_3**	**params_4**
ctriad				
dde				
delaunay	total; cartesian; average; frequency; number	product; distance; instances		
disorderb				
disorderc				
dist_freq	dn	10; 50; 100; 20; 5	dc	10; 50; 100; 20; 5
distance	distribution			
dpc				
eaac	window	6; 4; 3; 1; 2; 5		
egaac	window	8; 7; 6; 4; 3; 1; 2; 5		
electrostatic_hull		9; 12; 6; 3; 0		
fft	aaindex	WOLS870102; ROBB760111; RICJ880104; GEOR030106; FASG760103; ZIMJ680104; KHAG800101; VASM830101; QIAN880101; BUNA790103; AURR980115; QIAN880102; RACS820107; GEOR030103; BUNA790102; QIAN880103; AURR980118; RACS820102; KUMS000103; FINA910104; QIAN880117		

encoding	params_1	params_2	params_3	params_4
fldpc	aaindex	WOLS870102; ROBB760111; RICJ880104; GEOR030106; FASG760103; ZIMJ680104; KHAG800101; VASM830101; QIAN880101; BUNA790103; AURR980115; QIAN880102; RACS820107; GEOR030103; BUNA790102; QIAN880103; AURR980118; RACS820102; KUMS000103; FINA910104; QIAN880117		
flgc	aaindex	WOLS870102; ROBB760111; RICJ880104; GEOR030106; FASG760103; ZIMJ680104; KHAG800101; VASM830101; QIAN880101; BUNA790103; AURR980115; QIAN880102; RACS820107; GEOR030103; BUNA790102; QIAN880103; AURR980118; RACS820102; KUMS000103; FINA910104; QIAN880117		

encoding	params_1	params_2	params_3	params_4
gaac				
gdpc				
geary	nlag	6; 4; 3; 1; 2; 5		
gtpc				
ksctriad	gap	1; 2		
moran	nlag	6; 4; 3; 1; 2; 5		
ngram	e3; a2; s2; s3; a3; e2	200; 50; 1; 100; 20; 5; 300		
nmbroto	nlag	6; 4; 3; 1; 2; 5		
paac	lambda	6; 4; 3; 1; 2; 5		
qsar				
qsorder	nlag	6; 4; 3; 1; 2; 5		
socnumber	nlag	6; 4; 3; 1; 2; 5		
sseb				
ssec				
psekraac t1	st-g-gap	rt-7	ktu-1	la-2
psekraac t10	st-lambda-correlation	rt-18	ktu-1	la-2
psekraac t11	st-g-gap	rt-8	ktu-1	la-3
psekraac t12	st-lambda-correlation	rt-8	ktu-1	la-2
psekraac t13	st-lambda-correlation	rt-20	ktu-1	la-3
psekraac t14	st-g-gap	rt-10	ktu-2	la-1
psekraac t15	st-lambda-correlation	rt-15	ktu-2	la-2
psekraac t16	st-g-gap	rt-8	ktu-1	la-2
psekraac t2	st-lambda-correlation	rt-8	ktu-1	la-2
psekraac t3A	st-g-gap	rt-13	ktu-2	la-3
psekraac t3B	st-g-gap	rt-11	ktu-2	la-2

encoding	params_1	params_2	params_3	params_4
psekraac t4	st-g-gap	rt-9	ktu-1	la-1
psekraac t5	st-lambda-correlation	rt-15	ktu-3	la-1
psekraac t6A	st-lambda-correlation	rt-20	ktu-1	la-1
psekraac t6B	st-lambda-correlation	rt-5	ktu-3	la-3
psekraac t6C	st-lambda-correlation	rt-5	ktu-1	la-1
psekraac t7	st-lambda-correlation	rt-10	ktu-2	la-3
psekraac t8	st-lambda-correlation	rt-12	ktu-3	la-3
psekraac t9	st-g-gap	rt-13	ktu-2	la-2
ta				
tpc				
waac	aaindex	WOLS870102; ROBB760111; RICJ880104; GEOR030106; FASG760103; ZIMJ680104; KHAG800101; VASM830101; QIAN880101; BUNA790103; AURR980115; QIAN880102; RACS820107; GEOR030103; BUNA790102; QIAN880103; AURR980118; RACS820102; KUMS000103; FINA910104; QIAN880117		
zscale				

Statistics

anova_summary_aov

	term	df	sumsq	meansq	statistic	p.value	experiment
**1**	model	3	12.642499	4.214166	1207.763901	0.0	anova_summary_aov
**2**	Residuals	396	1.381735	0.003489	-	-	anova_summary_aov

anova_tukey_hsd

	term	contrast	null.value	estimate	conf.low	conf.high	adj.p.value	experiment
**1**	model	dt-bayes	0	-0.337852	-0.359404	-0.316299	0.000000e+00	anova_tukey_hsd
**2**	model	lr-bayes	0	-0.288467	-0.310019	-0.266914	0.000000e+00	anova_tukey_hsd
**3**	model	rf-bayes	0	-0.491206	-0.512758	-0.469654	0.000000e+00	anova_tukey_hsd
**4**	model	lr-dt	0	0.049385	0.027833	0.070938	4.378093e-08	anova_tukey_hsd
**5**	model	rf-dt	0	-0.153354	-0.174907	-0.131802	0.000000e+00	anova_tukey_hsd
**6**	model	rf-lr	0	-0.202740	-0.224292	-0.181187	0.000000e+00	anova_tukey_hsd

anova_error_summary_aov

	term	df	sumsq	meansq	statistic	p.value	experiment
**1**	model	4	256.474181	64.118545	49177.384337	0.0	anova_error_summary_aov
**2**	Residuals	500917	653.106499	0.001304	-	-	anova_error_summary_aov

anova_error_tukey_hsd

	term	contrast	null.value	estimate	conf.low	conf.high	adj.p.value	experiment
**1**	model	dt-bayes	0	-0.004087	-0.004527	-0.003647	0	anova_error_tukey_hsd
**2**	model	lr-bayes	0	-0.025613	-0.026053	-0.025173	0	anova_error_tukey_hsd
**3**	model	mlp-bayes	0	-0.036678	-0.037118	-0.036238	0	anova_error_tukey_hsd
**4**	model	rf-bayes	0	-0.061923	-0.062363	-0.061483	0	anova_error_tukey_hsd
**5**	model	lr-dt	0	-0.021526	-0.021966	-0.021086	0	anova_error_tukey_hsd
**6**	model	mlp-dt	0	-0.032591	-0.033031	-0.032151	0	anova_error_tukey_hsd
**7**	model	rf-dt	0	-0.057836	-0.058276	-0.057396	0	anova_error_tukey_hsd
**8**	model	mlp-lr	0	-0.011065	-0.011505	-0.010625	0	anova_error_tukey_hsd
**9**	model	rf-lr	0	-0.036310	-0.036750	-0.035870	0	anova_error_tukey_hsd
**10**	model	rf-mlp	0	-0.025244	-0.025685	-0.024804	0	anova_error_tukey_hsd

anova_kappa_summary_aov

	term	df	sumsq	meansq	statistic	p.value	experiment
**1**	model	4	1054.130066	263.532516	9952.23633	0.0	anova_kappa_summary_aov
**2**	Residuals	500917	13264.146186	0.026480	-	-	anova_kappa_summary_aov

anova_kappa_tukey_hsd

	term	contrast	null.value	estimate	conf.low	conf.high	adj.p.value	experiment
**1**	model	dt-bayes	0	-0.050541	-0.052525	-0.048558	0.000000	anova_kappa_tukey_hsd
**2**	model	lr-bayes	0	-0.000921	-0.002904	0.001062	0.711737	anova_kappa_tukey_hsd
**3**	model	mlp-bayes	0	0.009702	0.007719	0.011685	0.000000	anova_kappa_tukey_hsd
**4**	model	rf-bayes	0	0.091479	0.089495	0.093462	0.000000	anova_kappa_tukey_hsd
**5**	model	lr-dt	0	0.049620	0.047637	0.051604	0.000000	anova_kappa_tukey_hsd
**6**	model	mlp-dt	0	0.060243	0.058260	0.062227	0.000000	anova_kappa_tukey_hsd
**7**	model	rf-dt	0	0.142020	0.140037	0.144003	0.000000	anova_kappa_tukey_hsd
**8**	model	mlp-lr	0	0.010623	0.008640	0.012606	0.000000	anova_kappa_tukey_hsd
**9**	model	rf-lr	0	0.092400	0.090416	0.094383	0.000000	anova_kappa_tukey_hsd
**10**	model	rf-mlp	0	0.081777	0.079793	0.083760	0.000000	anova_kappa_tukey_hsd

manova_summary

	term	df	pillai	statistic	num.df	den.df	p.value	experiment
**1**	model	4	0.265087	20227.538544	8.0	1059066.0	0.0	manova_summary
**2**	Residuals	529533	-	-	-	-	-	manova_summary

manova_summary_aov

	Df	Sum.Sq	Mean.Sq	F.value	Pr..F.	response	experiment
**model**	4	1082.229526	270.557381	8904.276366	0.0	Response 1	manova_summary_aov
**Residuals**	529533	16089.916352	0.030385	-	-	Response 1	manova_summary_aov
**model 1**	4	268.059924	67.014981	43190.894647	0.0	Response 2	manova_summary_aov
**Residuals 1**	529533	821.623268	0.001552	-	-	Response 2	manova_summary_aov

Plots

Refer to main manuscript for more details.

Suppl. Fig. 1. MVO fitness vs. generations.

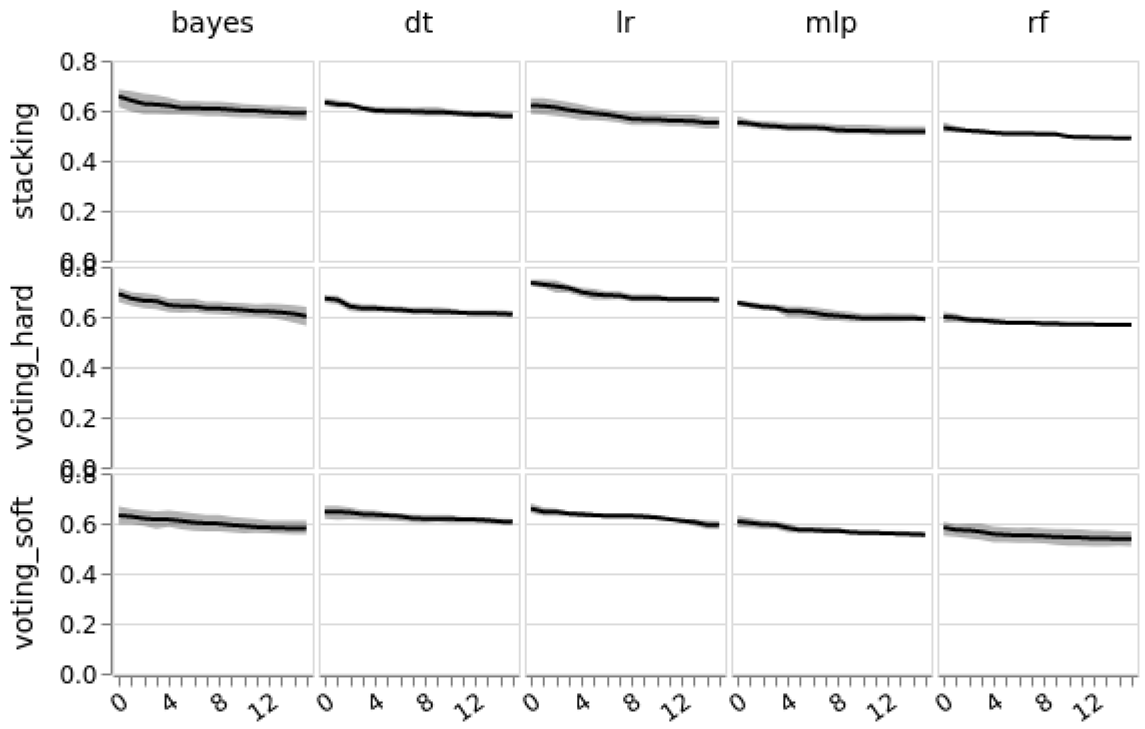

Suppl. Fig. 2. XCD chart

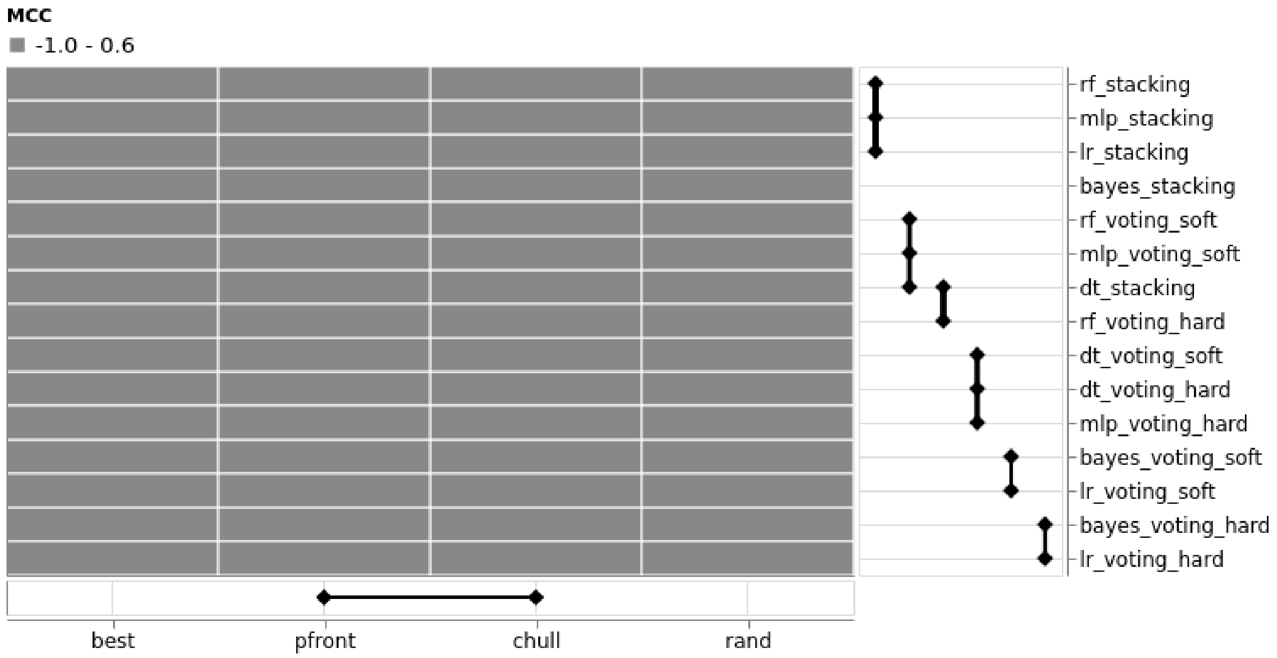

Suppl. Fig. 3. Boxplot

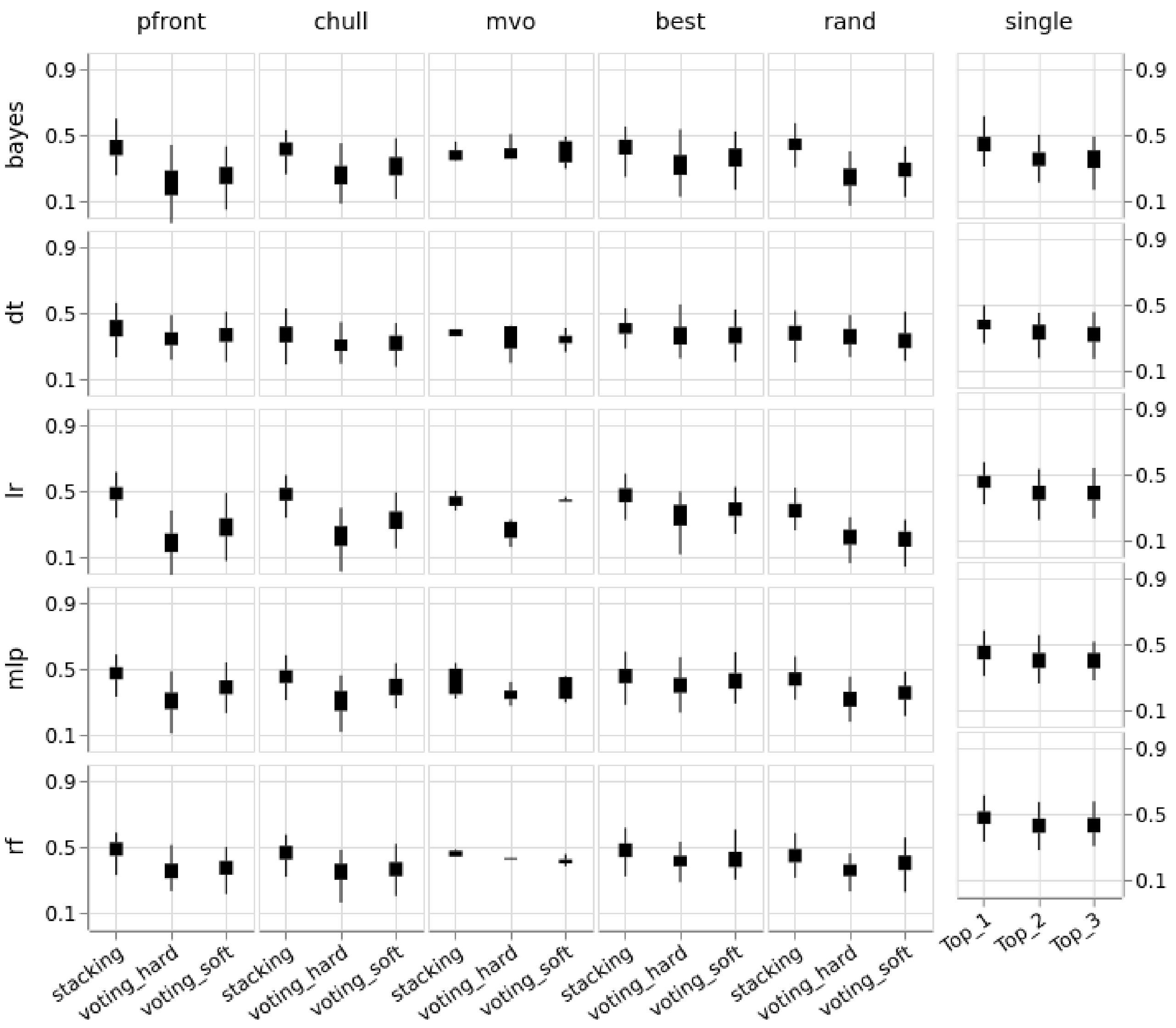

Suppl. Fig. 4. Kappa-error plot

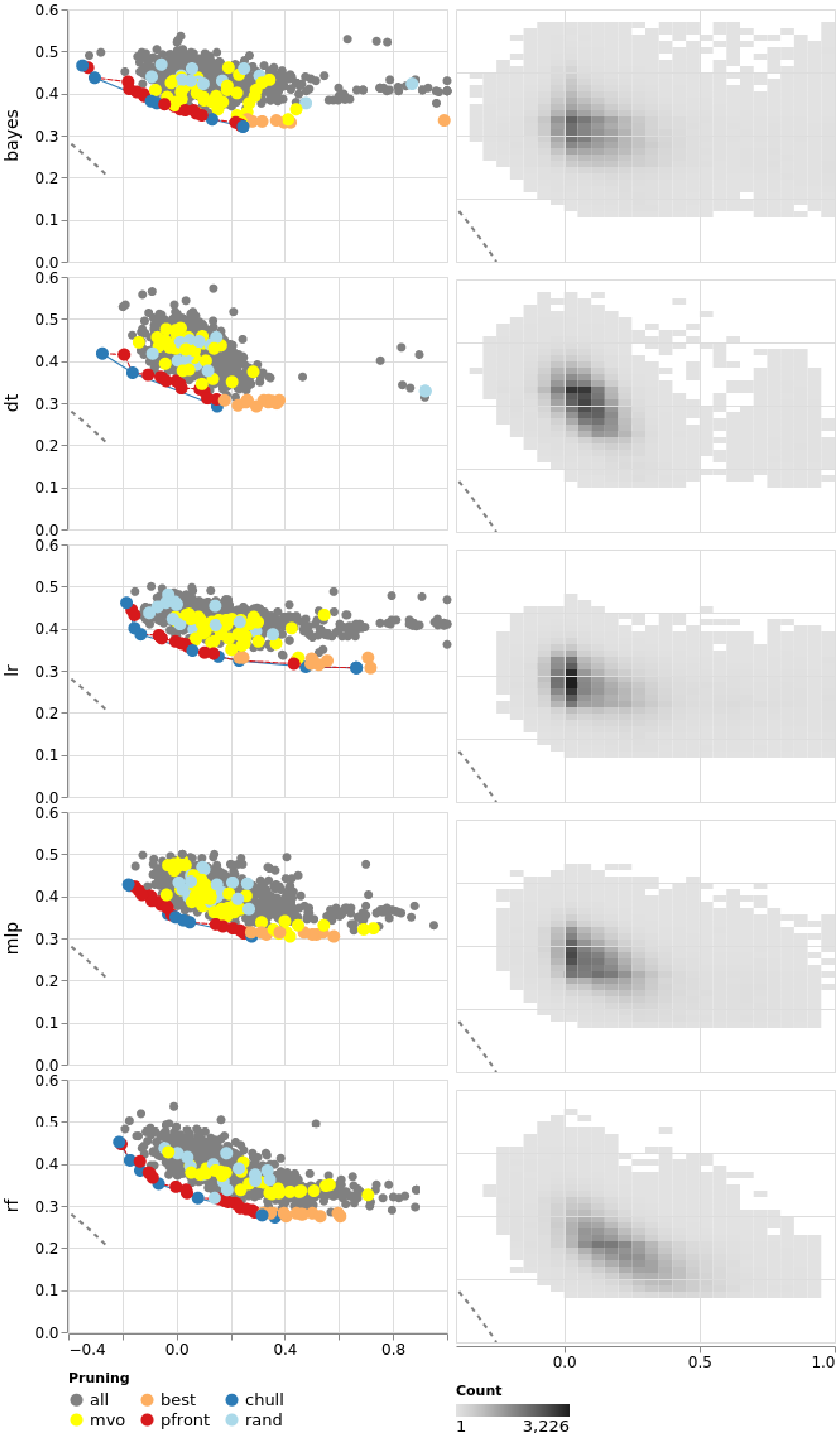
bayes
dt
lr
mlp
rf
0.0
0.1
0.2
0.3
0.4
0.5
0.6
−0.4
0.0
0.4
0.8
0.5
1.0
Pruning
all
best
chull
mvo
pfront
rand
Count
1
3,226

Suppl. Fig. 5. Boxplot MANOVA

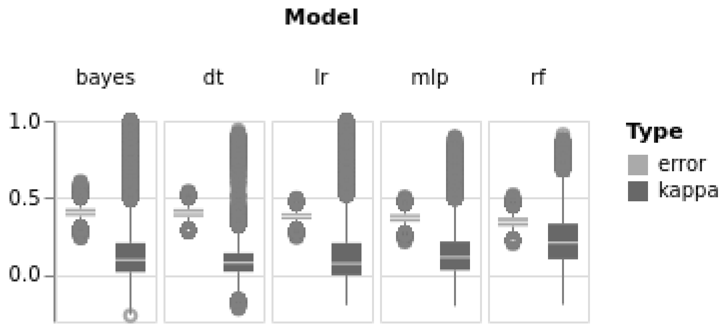

Supplement: Supplementary file 1 — Additional file 1. [file 13040_2022_317_MOESM1_ESM.zip › supplements/aip_antiinflamR1.pdf]
