# Supplementary material for: Unsupervised encoding selection through ensemble pruning for biomedical classification
Source: BioData Min. 2023 Mar 16;16:10. doi: 10.1186/s13040-022-00317-7 (PMC10018861; doi:10.1186/s13040-022-00317-7)

List of encodings

Refer to Spänig *et al.* (2021) for more details (https://doi.org/10.1093/nargab/lqab039).

encoding	params_1	params_2	params_3	params_4
aac				
aaindex	QIAN880102; WOLS870102; AURR980118; RACS820107; RICJ880104; FASG760103; QIAN880103; RACS820102; BUNA790102; KUMS000103; AURR980115; QIAN880101; ROBB760111; GEOR030106; KHAG800101; ZIMJ680104; FINA910104; QIAN880117; VASM830101; BUNA790103; GEOR030103			
apaac	lambda	2; 3; 1; 5; 4		
asa				
binary				
blomap				
blosum62				
cgr	res	100; 20; 200; 10	sf	0.8632713; 0.5
cksaagp	gap	1; 3; 4; 2		
cksaap	gap	1; 3; 4; 2		
ctdc				
ctdd				
ctdt				

encoding	params_1	params_2	params_3	params_4
ctriad				
dde				
delaunay	cartesian; total; number; average; frequency	distance; instances; product		
disorderb				
disorderc				
dist_freq	dn	100; 10; 50; 5; 20	dc	100; 10; 50; 5; 20
distance	distribution			
dpc				
eaac	window	2; 3; 1; 5; 4		
egaac	window	8; 2; 3; 7; 6; 1; 5; 4		
electrostatic_hull		9; 6; 3; 0; 12		
fft	aaindex	QIAN880102; WOLS870102; AURR980118; RACS820107; RICJ880104; FASG760103; QIAN880103; RACS820102; BUNA790102; KUMS000103; AURR980115; QIAN880101; ROBB760111; GEOR030106; KHAG800101; ZIMJ680104; FINA910104; QIAN880117; VASM830101; BUNA790103; GEOR030103		

encoding	params_1	params_2	params_3	params_4
fldpc	aaindex	QIAN880102; WOLS870102; AURR980118; RACS820107; RICJ880104; FASG760103; QIAN880103; RACS820102; BUNA790102; KUMS000103; AURR980115; QIAN880101; ROBB760111; GEOR030106; KHAG800101; ZIMJ680104; FINA910104; QIAN880117; VASM830101; BUNA790103; GEOR030103		
flgc	aaindex	QIAN880102; WOLS870102; AURR980118; RACS820107; RICJ880104; FASG760103; QIAN880103; RACS820102; BUNA790102; KUMS000103; AURR980115; QIAN880101; ROBB760111; GEOR030106; KHAG800101; ZIMJ680104; FINA910104; QIAN880117; VASM830101; BUNA790103; GEOR030103		

encoding	params_1	params_2	params_3	params_4
gaac				
gdpc				
geary	nlag	2; 3; 1; 5; 4		
gtpc				
ksctriad	gap	1		
moran	nlag	2; 3; 1; 5; 4		
ngram	a2; a3; e2; e3; s3; s2	100; 300; 200; 50; 1; 5; 20		
nmbroto	nlag	2; 3; 1; 5; 4		
paac	lambda	2; 3; 1; 5; 4		
qsar				
qsorder	nlag	2; 3; 1; 5; 4		
socnumber	nlag	2; 3; 1; 5; 4		
sseb				
ssec				
psekraac t1	st-lambda-correlation	rt-10	ktu-3	la-3
psekraac t10	st-lambda-correlation	rt-8	ktu-1	la-2
psekraac t11	st-g-gap	rt-9	ktu-1	la-2
psekraac t12	st-lambda-correlation	rt-8	ktu-1	la-3
psekraac t13	st-g-gap	rt-12	ktu-1	la-3
psekraac t14	st-g-gap	rt-10	ktu-1	la-1
psekraac t15	st-g-gap	rt-13	ktu-1	la-2
psekraac t16	st-g-gap	rt-11	ktu-2	la-3
psekraac t2	st-g-gap	rt-2	ktu-1	la-2
psekraac t3A	st-g-gap	rt-13	ktu-3	la-2
psekraac t3B	st-lambda-correlation	rt-11	ktu-3	la-2
psekraac t4	st-g-gap	rt-9	ktu-1	la-2

**encoding**	**params_1**	**params_2**	**params_3**	**params_4**
psekraac t5	st-g-gap	rt-20	ktu-1	la-3
psekraac t6A	st-g-gap	rt-20	ktu-1	la-3
psekraac t6B	st-lambda-correlation	rt-5	ktu-1	la-2
psekraac t6C	st-g-gap	rt-5	ktu-3	la-3
psekraac t7	st-lambda-correlation	rt-10	ktu-1	la-3
psekraac t8	st-g-gap	rt-2	ktu-3	la-3
psekraac t9	st-g-gap	rt-11	ktu-2	la-3
ta				
tpc				
waac	aaindex	QIAN880102; WOLS870102; AURR980118; RACS820107; RICJ880104; FASG760103; QIAN880103; RACS820102; BUNA790102; KUMS000103; AURR980115; QIAN880101; ROBB760111; GEOR030106; KHAG800101; ZIMJ680104; FINA910104; QIAN880117; VASM830101; BUNA790103; GEOR030103		
zscale				

Statistics

anova_summary_aov

	term	df	sumsq	meansq	statistic	p.value	experiment
**1**	model	3	14.210452	4.736817	492.746477	0.0	anova_summary_aov
**2**	Residuals	396	3.806784	0.009613	-	-	anova_summary_aov

anova_tukey_hsd

	term	contrast	null.value	estimate	conf.low	conf.high	adj.p.value	experiment
**1**	model	dt-bayes	0	-0.389398	-0.425171	-0.353624	0.000000	anova_tukey_hsd
**2**	model	lr-bayes	0	0.064970	0.029196	0.100743	0.000023	anova_tukey_hsd
**3**	model	rf-bayes	0	-0.276814	-0.312587	-0.241040	0.000000	anova_tukey_hsd
**4**	model	lr-dt	0	0.454367	0.418594	0.490141	0.000000	anova_tukey_hsd
**5**	model	rf-dt	0	0.112584	0.076810	0.148357	0.000000	anova_tukey_hsd
**6**	model	rf-lr	0	-0.341783	-0.377557	-0.306010	0.000000	anova_tukey_hsd

anova_error_summary_aov

	term	df	sumsq	meansq	statistic	p.value	experiment
**1**	model	4	720.557905	180.139476	32821.142607	0.0	anova_error_summary_aov
**2**	Residuals	500942	2749.429858	0.005489	-	-	anova_error_summary_aov

anova_error_tukey_hsd

	term	contrast	null.value	estimate	conf.low	conf.high	adj.p.value	experiment
**1**	model	dt-bayes	0	-0.024618	-0.025521	-0.023715	0	anova_error_tukey_hsd
**2**	model	lr-bayes	0	-0.035425	-0.036329	-0.034522	0	anova_error_tukey_hsd
**3**	model	mlp-bayes	0	-0.074667	-0.075570	-0.073764	0	anova_error_tukey_hsd
**4**	model	rf-bayes	0	-0.106915	-0.107817	-0.106012	0	anova_error_tukey_hsd
**5**	model	lr-dt	0	-0.010807	-0.011710	-0.009904	0	anova_error_tukey_hsd
**6**	model	mlp-dt	0	-0.050049	-0.050952	-0.049146	0	anova_error_tukey_hsd
**7**	model	rf-dt	0	-0.082297	-0.083199	-0.081394	0	anova_error_tukey_hsd
**8**	model	mlp-lr	0	-0.039242	-0.040145	-0.038339	0	anova_error_tukey_hsd
**9**	model	rf-lr	0	-0.071489	-0.072392	-0.070586	0	anova_error_tukey_hsd
**10**	model	rf-mlp	0	-0.032248	-0.033150	-0.031345	0	anova_error_tukey_hsd

anova_kappa_summary_aov

	term	df	sumsq	meansq	statistic	p.value	experiment
**1**	model	4	3287.209131	821.802283	18925.651386	0.0	anova_kappa_summary_aov
**2**	Residuals	500942	21752.238309	0.043423	-	-	anova_kappa_summary_aov

anova_kappa_tukey_hsd

	term	contrast	null.value	estimate	conf.low	conf.high	adj.p.value	experiment
**1**	model	dt-bayes	0	0.022282	0.019743	0.024821	0	anova_kappa_tukey_hsd
**2**	model	lr-bayes	0	0.080924	0.078384	0.083464	0	anova_kappa_tukey_hsd
**3**	model	mlp-bayes	0	0.136849	0.134309	0.139388	0	anova_kappa_tukey_hsd
**4**	model	rf-bayes	0	0.223703	0.221163	0.226242	0	anova_kappa_tukey_hsd
**5**	model	lr-dt	0	0.058642	0.056102	0.061182	0	anova_kappa_tukey_hsd
**6**	model	mlp-dt	0	0.114567	0.112028	0.117106	0	anova_kappa_tukey_hsd
**7**	model	rf-dt	0	0.201421	0.198882	0.203960	0	anova_kappa_tukey_hsd
**8**	model	mlp-lr	0	0.055925	0.053385	0.058465	0	anova_kappa_tukey_hsd
**9**	model	rf-lr	0	0.142779	0.140239	0.145319	0	anova_kappa_tukey_hsd
**10**	model	rf-mlp	0	0.086854	0.084315	0.089394	0	anova_kappa_tukey_hsd

manova_summary

	term	df	pillai	statistic	num.df	den.df	p.value	experiment
**1**	model	4	0.194732	14707.450615	8.0	1090768.0	0.0	manova_summary
**2**	Residuals	545384	-	-	-	-	-	manova_summary

manova_summary_aov

	Df	Sum.Sq	Mean.Sq	F.value	Pr..F.	response	experiment
**model**	4	3423.815265	855.953816	17016.042121	0.0	Response 1	manova_summary_aov
**Residuals**	545384	27434.318323	0.050303	-	-	Response 1	manova_summary_aov
**model 1**	4	757.666970	189.416742	30181.148919	0.0	Response 2	manova_summary_aov
**Residuals 1**	545384	3422.827307	0.006276	-	-	Response 2	manova_summary_aov

Plots

Refer to main manuscript for more details.

Suppl. Fig. 1. MVO fitness vs. generations.

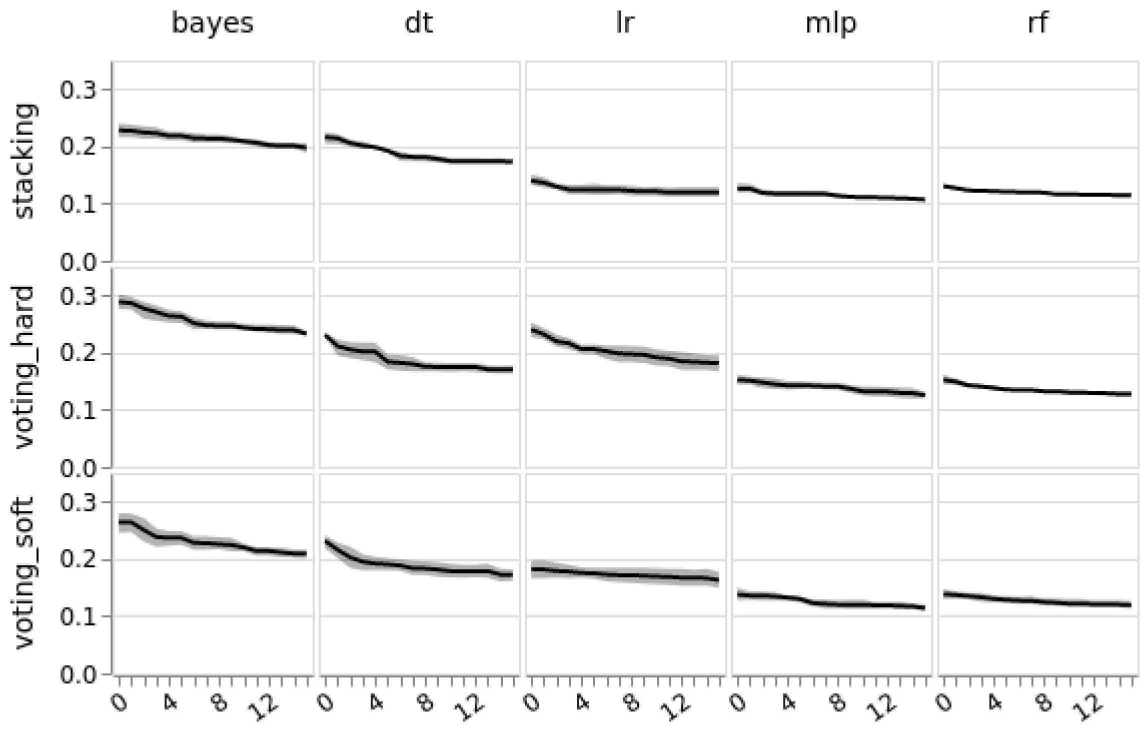

Suppl. Fig. 2. XCD chart

Suppl. Fig. 3. Boxplot

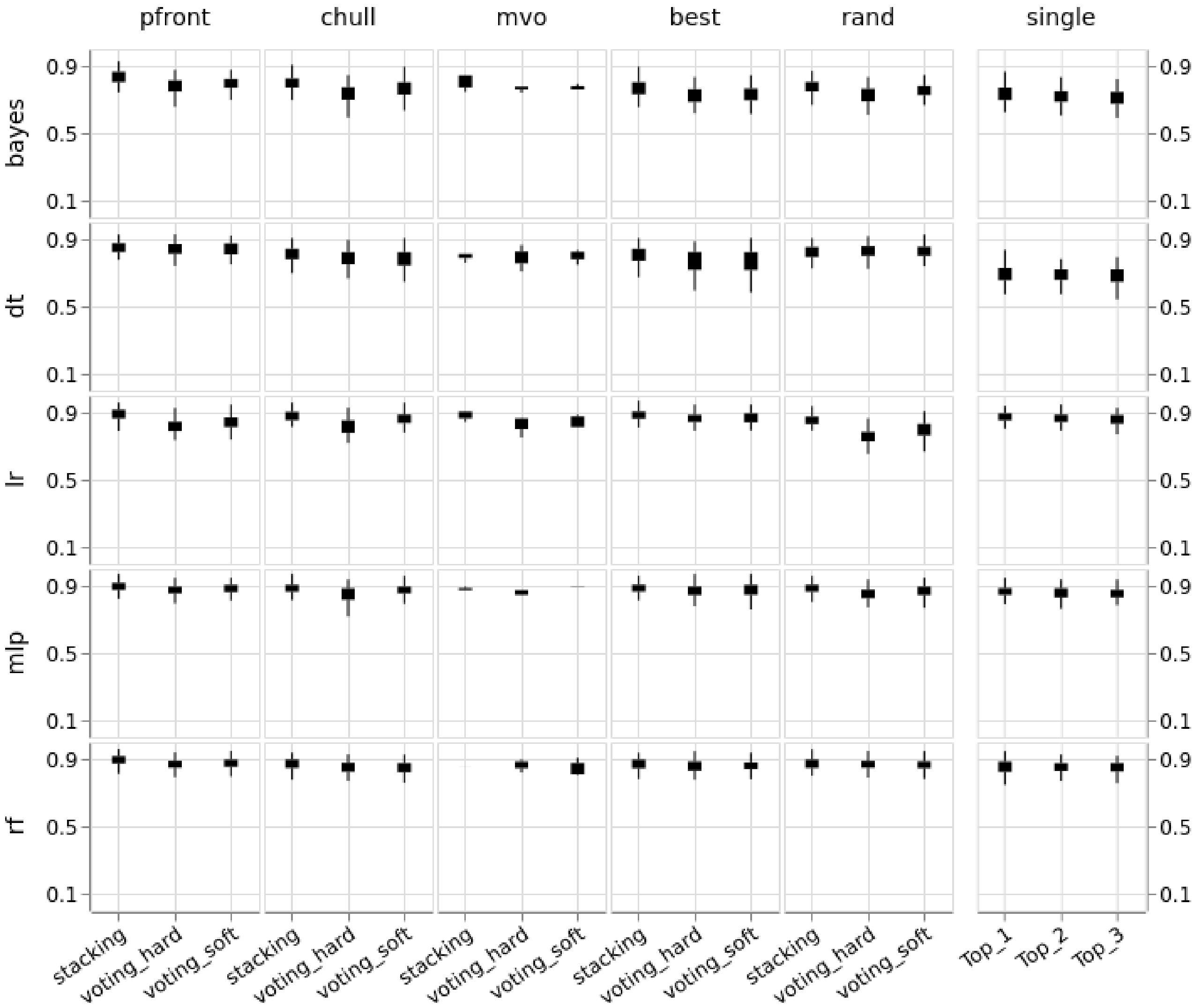

Suppl. Fig. 4. Kappa-error plot

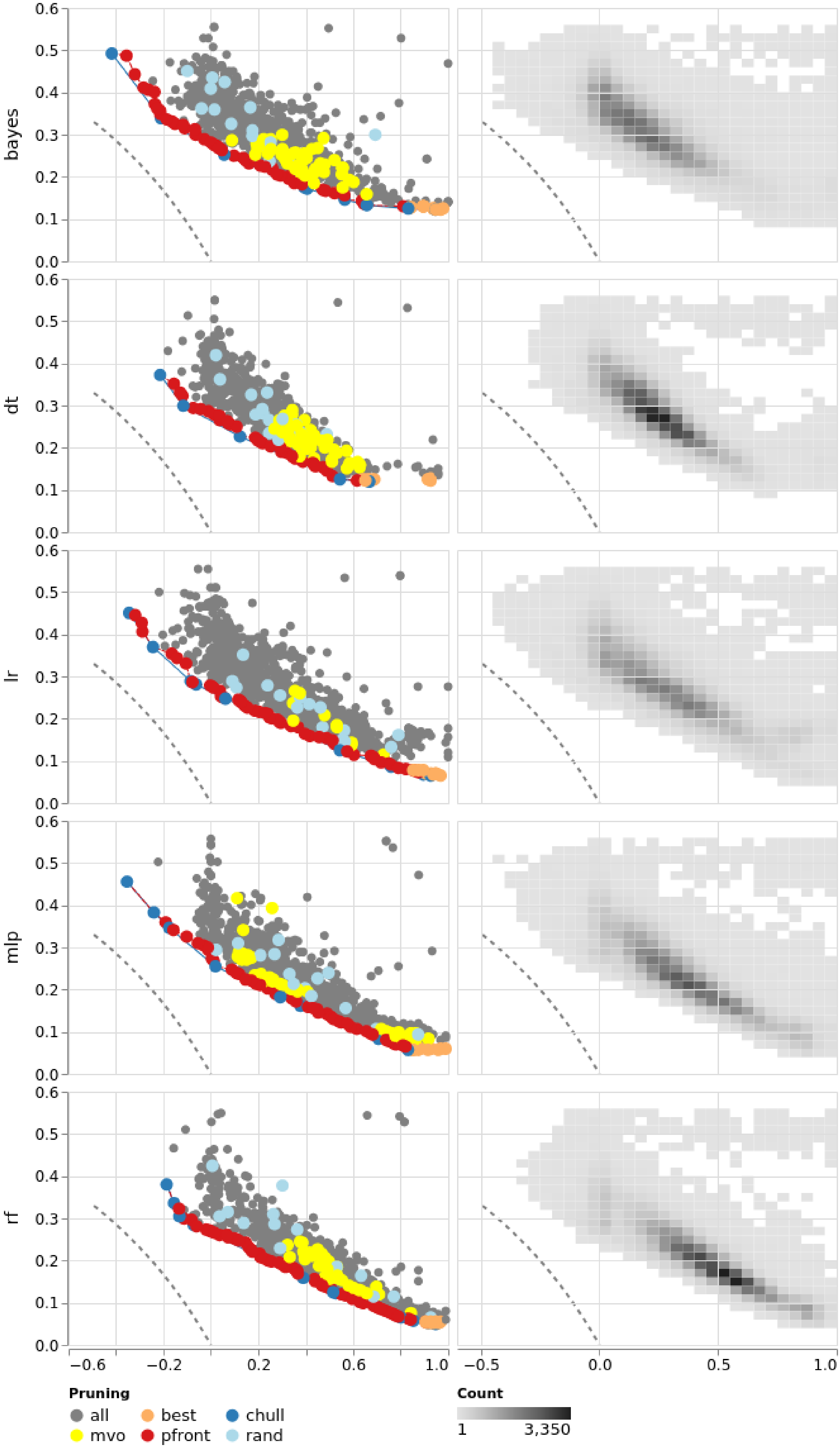

bayes
dt
lr
mlp
rf
0.0
0.1
0.2
0.3
0.4
0.5
0.6
−0.6
−0.2
0.2
0.6
1.0
−0.5
0.0
0.5
1.0
Pruning
all
best
chull
mvo
pfront
rand
Count
1
3,350

Suppl. Fig. 5. Boxplot MANOVA

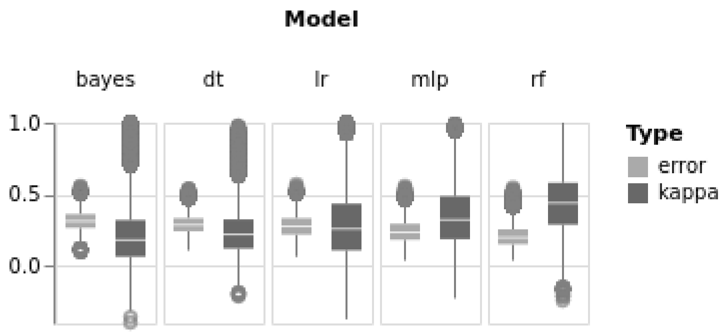

Supplement: Supplementary file 1 — Additional file 1. [file 13040_2022_317_MOESM1_ESM.zip › supplements/amp_antibp2R1.pdf]
