# Supplementary material for: Unsupervised encoding selection through ensemble pruning for biomedical classification
Source: BioData Min. 2023 Mar 16;16:10. doi: 10.1186/s13040-022-00317-7 (PMC10018861; doi:10.1186/s13040-022-00317-7)
Supplement: Supplementary file 1 — Additional file 1. [file 13040_2022_317_MOESM1_ESM.zip › supplements/atb_antitbpR1.pdf]

List of encodings

Refer to Spänig *et al.* (2021) for more details (https://doi.org/10.1093/nargab/lqab039).

**encoding**	**params_1**	**params_2**	**params_3**	**params_4**
aac				
aaindex	QIAN880101; QIAN880103; GEOR030103; GEOR030106; KHAG800101; ZIMJ680104; RICJ880104; RACS820107; RACS820102; BUNA790102; AURR980115; AURR980118; WOLS870102; KUMS000103; VASM830101; QIAN880117; FASG760103; FINA910104; BUNA790103; ROBB760111; QIAN880102			
apaac	lambda	2; 4; 1; 3		
asa				
binary				
blomap				
blosum62				
cgr	res	10; 200; 20; 100	sf	0.5; 0.8632713
cksaagp	gap	2; 1; 3		
cksaap	gap	2; 1; 3		
ctdc				
ctdd				
ctdt				

encoding	params_1	params_2	params_3	params_4
ctriad				
dde				
delaunay	average; number; frequency; total; cartesian	distance; product; instances		
disorderb				
disorderc				
dist_freq	dn	20; 100; 5; 10; 50	dc	20; 100; 5; 10; 50
distance	distribution			
dpc				
eaac	window	2; 4; 1; 3		
egaac	window	3; 4; 8; 1; 6; 7; 5; 2		
electrostatic_hull		3; 6; 0; 12; 9		
fft	aaindex	QIAN880101; QIAN880103; GEOR030103; GEOR030106; KHAG800101; ZIMJ680104; RICJ880104; RACS820107; RACS820102; BUNA790102; AURR980115; AURR980118; WOLS870102; KUMS000103; VASM830101; QIAN880117; FASG760103; FINA910104; BUNA790103; ROBB760111; QIAN880102		

encoding	params_1	params_2	params_3	params_4
fldpc	aaindex	QIAN880101; QIAN880103; GEOR030103; GEOR030106; KHAG800101; ZIMJ680104; RICJ880104; RACS820107; RACS820102; BUNA790102; AURR980115; AURR980118; WOLS870102; KUMS000103; VASM830101; QIAN880117; FASG760103; FINA910104; BUNA790103; ROBB760111; QIAN880102		
flgc	aaindex	QIAN880101; QIAN880103; GEOR030103; GEOR030106; KHAG800101; ZIMJ680104; RICJ880104; RACS820107; RACS820102; BUNA790102; AURR980115; AURR980118; WOLS870102; KUMS000103; VASM830101; QIAN880117; FASG760103; FINA910104; BUNA790103; ROBB760111; QIAN880102		

**encoding**	**params_1**	**params_2**	**params_3**	**params_4**
gaac				
gdpc				
geary	nlag	2; 4; 1; 3		
gtpc				
ksctriad	gap	1		
moran	nlag	2; 4; 1; 3		
ngram	a2; s3; e2; s2; e3; a3	20; 1; 300; 200; 100; 5; 50		
nmbroto	nlag	2; 4; 1; 3		
paac	lambda	2; 4; 1; 3		
qsar				
qsorder	nlag	2; 4; 1; 3		
socnumber	nlag	2; 4; 1; 3		
sseb				
ssec				
psekraac t1	st-g-gap	rt-10	ktu-2	la-3
psekraac t10	st-lambda-correlation	rt-10	ktu-1	la-3
psekraac t11	st-g-gap	rt-12	ktu-1	la-3
psekraac t12	st-lambda-correlation	rt-8	ktu-1	la-2
psekraac t13	st-g-gap	rt-17	ktu-1	la-1
psekraac t14	st-g-gap	rt-10	ktu-1	la-1
psekraac t15	st-lambda-correlation	rt-10	ktu-3	la-3
psekraac t16	st-lambda-correlation	rt-4	ktu-3	la-2
psekraac t2	st-lambda-correlation	rt-5	ktu-1	la-1
psekraac t3A	st-g-gap	rt-12	ktu-2	la-2
psekraac t3B	st-g-gap	rt-12	ktu-1	la-3

**encoding**	**params_1**	**params_2**	**params_3**	**params_4**
psekraac t4	st-lambda-correlation	rt-9	ktu-1	la-1
psekraac t5	st-g-gap	rt-15	ktu-1	la-2
psekraac t6A	st-lambda-correlation	rt-4	ktu-1	la-1
psekraac t6B	st-lambda-correlation	rt-5	ktu-2	la-1
psekraac t6C	st-lambda-correlation	rt-5	ktu-2	la-2
psekraac t7	st-lambda-correlation	rt-10	ktu-1	la-1
psekraac t8	st-g-gap	rt-12	ktu-1	la-3
psekraac t9	st-g-gap	rt-12	ktu-3	la-2
ta				
tpc				
waac	aaindex	QIAN880101; QIAN880103; GEOR030103; GEOR030106; KHAG800101; ZIMJ680104; RICJ880104; RACS820107; RACS820102; BUNA790102; AURR980115; AURR980118; WOLS870102; KUMS000103; VASM830101; QIAN880117; FASG760103; FINA910104; BUNA790103; ROBB760111; QIAN880102		
zscale				

Statistics

anova_summary_aov

	term	df	sumsq	meansq	statistic	p.value	experiment
**1**	model	3	8.936894	2.978965	262.420585	0.0	anova_summary_aov
**2**	Residuals	396	4.495341	0.011352	-	-	anova_summary_aov

anova_tukey_hsd

	term	contrast	null.value	estimate	conf.low	conf.high	adj.p.value	experiment
**1**	model	dt-bayes	0	-0.224144	-0.263019	-0.185270	0.000000	anova_tukey_hsd
**2**	model	lr-bayes	0	0.017693	-0.021182	0.056567	0.643506	anova_tukey_hsd
**3**	model	rf-bayes	0	-0.334706	-0.373580	-0.295832	0.000000	anova_tukey_hsd
**4**	model	lr-dt	0	0.241837	0.202963	0.280711	0.000000	anova_tukey_hsd
**5**	model	rf-dt	0	-0.110562	-0.149436	-0.071687	0.000000	anova_tukey_hsd
**6**	model	rf-lr	0	-0.352399	-0.391273	-0.313524	0.000000	anova_tukey_hsd

anova_error_summary_aov

	term	df	sumsq	meansq	statistic	p.value	experiment
**1**	model	4	292.083124	73.020781	12547.00129	0.0	anova_error_summary_aov
**2**	Residuals	500889	2915.063538	0.005820	-	-	anova_error_summary_aov

anova_error_tukey_hsd

	term	contrast	null.value	estimate	conf.low	conf.high	adj.p.value	experiment
**1**	model	dt-bayes	0	-0.007717	-0.008646	-0.006787	0	anova_error_tukey_hsd
**2**	model	lr-bayes	0	-0.019202	-0.020131	-0.018272	0	anova_error_tukey_hsd
**3**	model	mlp-bayes	0	-0.031162	-0.032091	-0.030232	0	anova_error_tukey_hsd
**4**	model	rf-bayes	0	-0.068841	-0.069771	-0.067911	0	anova_error_tukey_hsd
**5**	model	lr-dt	0	-0.011485	-0.012415	-0.010555	0	anova_error_tukey_hsd
**6**	model	mlp-dt	0	-0.023445	-0.024375	-0.022515	0	anova_error_tukey_hsd
**7**	model	rf-dt	0	-0.061124	-0.062054	-0.060194	0	anova_error_tukey_hsd
**8**	model	mlp-lr	0	-0.011960	-0.012890	-0.011030	0	anova_error_tukey_hsd
**9**	model	rf-lr	0	-0.049639	-0.050569	-0.048709	0	anova_error_tukey_hsd
**10**	model	rf-mlp	0	-0.037679	-0.038609	-0.036749	0	anova_error_tukey_hsd

anova_kappa_summary_aov

	term	df	sumsq	meansq	statistic	p.value	experiment
**1**	model	4	1785.513900	446.378475	8400.732492	0.0	anova_kappa_summary_aov
**2**	Residuals	500889	26615.068167	0.053136	-	-	anova_kappa_summary_aov

anova_kappa_tukey_hsd

	term	contrast	null.value	estimate	conf.low	conf.high	adj.p.value	experiment
**1**	model	dt-bayes	0	-0.024848	-0.027657	-0.022039	0	anova_kappa_tukey_hsd
**2**	model	lr-bayes	0	0.055541	0.052732	0.058350	0	anova_kappa_tukey_hsd
**3**	model	mlp-bayes	0	0.071390	0.068581	0.074199	0	anova_kappa_tukey_hsd
**4**	model	rf-bayes	0	0.146115	0.143305	0.148924	0	anova_kappa_tukey_hsd
**5**	model	lr-dt	0	0.080389	0.077580	0.083198	0	anova_kappa_tukey_hsd
**6**	model	mlp-dt	0	0.096238	0.093429	0.099048	0	anova_kappa_tukey_hsd
**7**	model	rf-dt	0	0.170963	0.168153	0.173773	0	anova_kappa_tukey_hsd
**8**	model	mlp-lr	0	0.015849	0.013040	0.018659	0	anova_kappa_tukey_hsd
**9**	model	rf-lr	0	0.090574	0.087764	0.093384	0	anova_kappa_tukey_hsd
**10**	model	rf-mlp	0	0.074725	0.071915	0.077535	0	anova_kappa_tukey_hsd

manova_summary

	term	df	pillai	statistic	num.df	den.df	p.value	experiment
**1**	model	4	0.090357	6277.0477	8.0	1061294.0	0.0	manova_summary
**2**	Residuals	530647	-	-	-	-	-	manova_summary

manova_summary_aov

	Df	Sum.Sq	Mean.Sq	F.value	Pr..F.	response	experiment
**model**	4	1810.256348	452.564087	7561.1941	0.0	Response 1	manova_summary_aov
**Residuals**	530647	31761.091152	0.059854	-	-	Response 1	manova_summary_aov
**model 1**	4	297.295193	74.323798	11301.886714	0.0	Response 2	manova_summary_aov
**Residuals 1**	530647	3489.656337	0.006576	-	-	Response 2	manova_summary_aov

Plots

Refer to main manuscript for more details.

Suppl. Fig. 1. MVO fitness vs. generations.

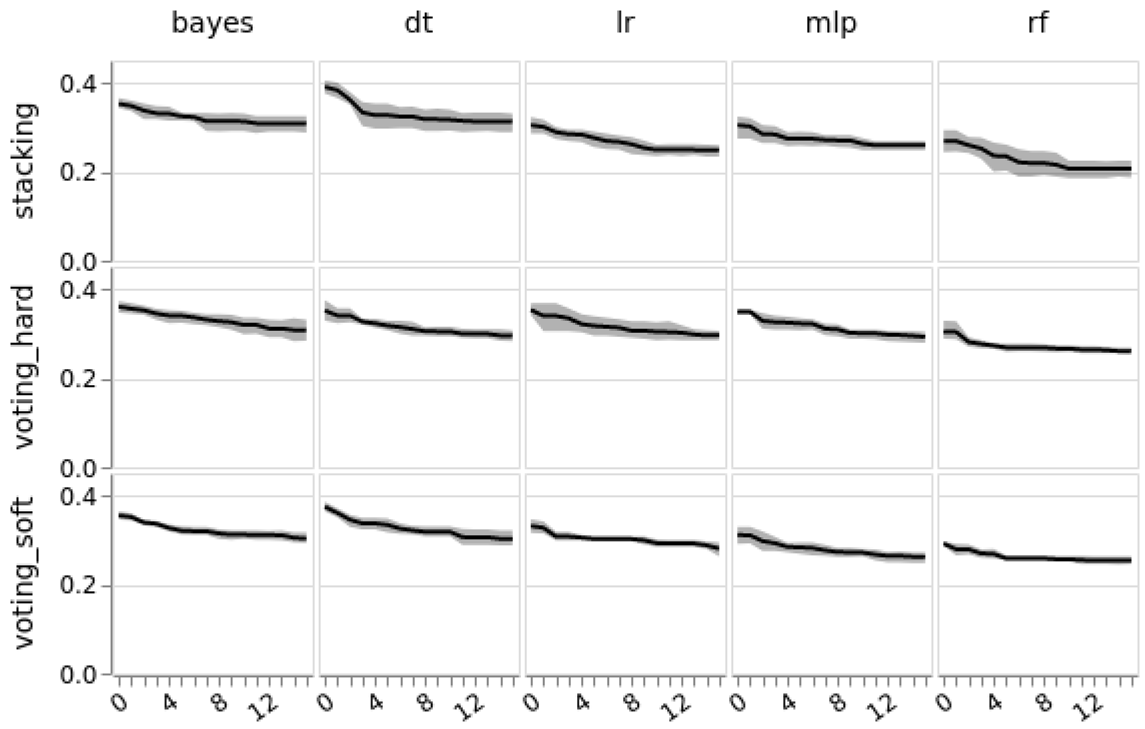

Suppl. Fig. 2. XCD chart

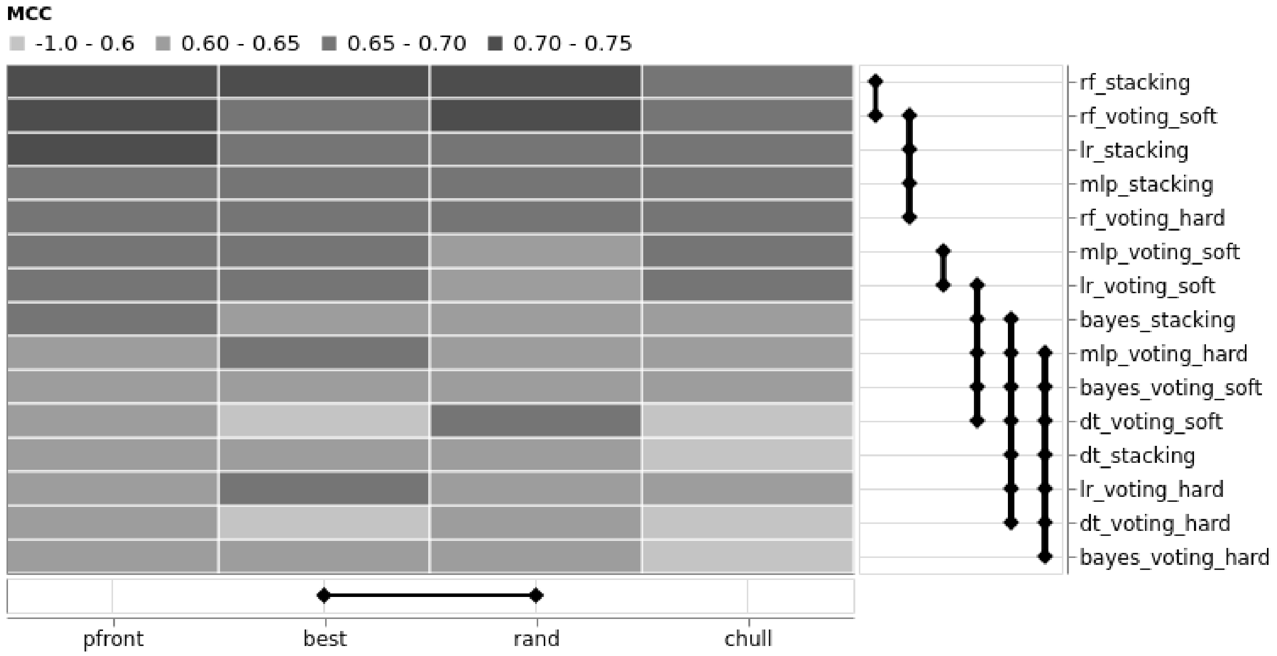

Suppl. Fig. 3. Boxplot

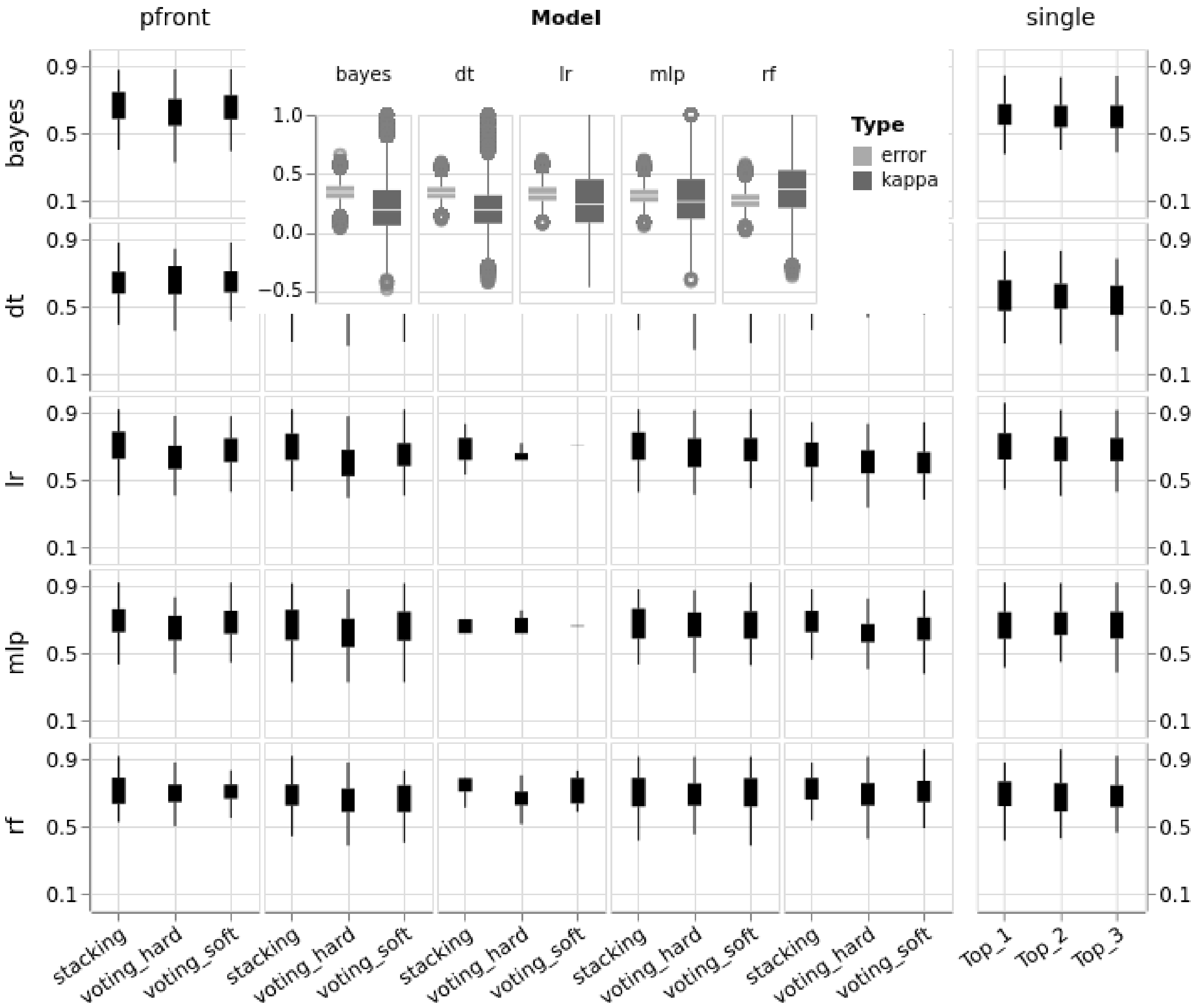

Suppl. Fig. 4. Kappa-error plot

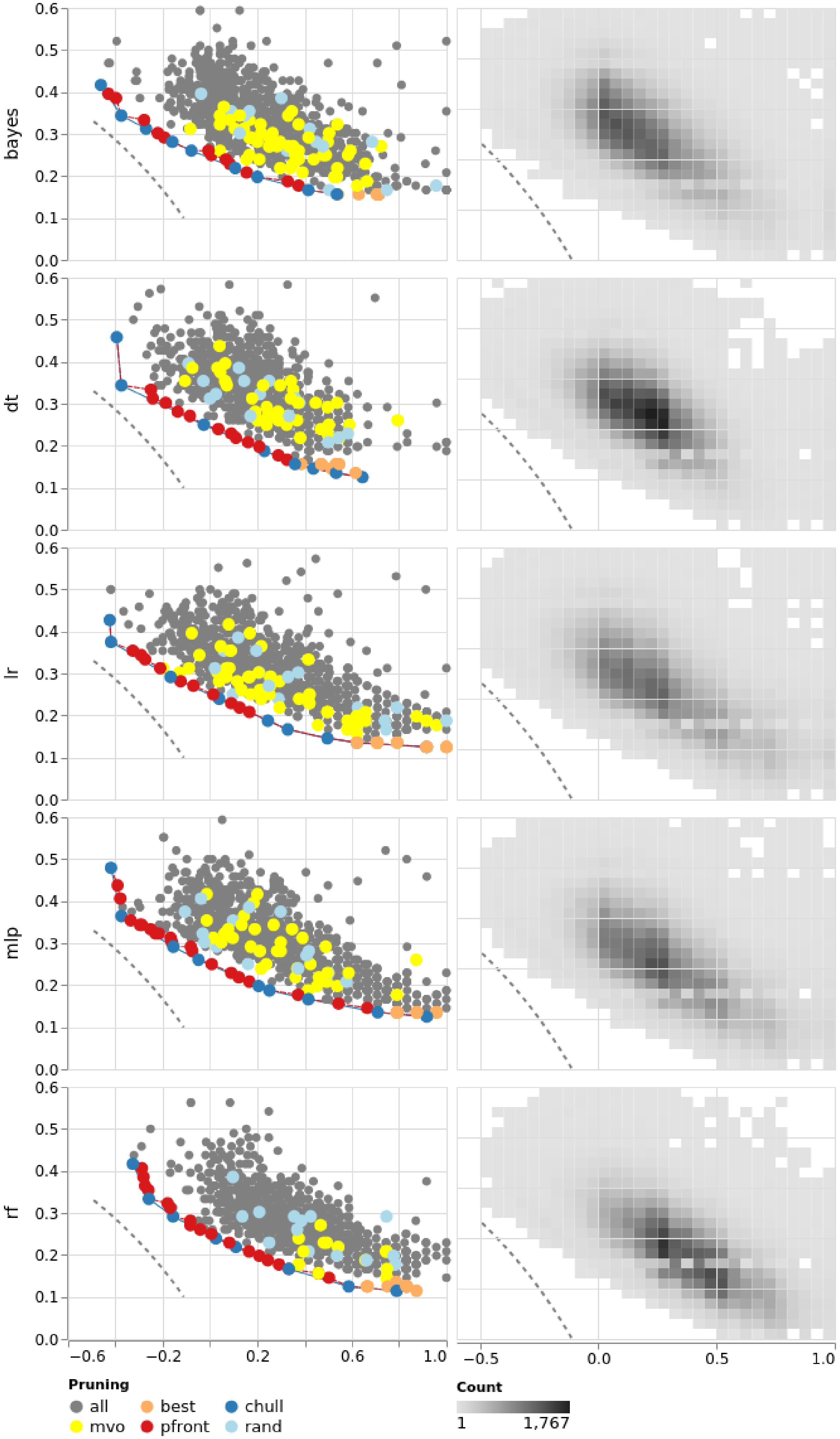

bayes
dt
lr
mlp
rf
0.0
0.1
0.2
0.3
0.4
0.5
0.6
−0.6
−0.2
0.2
0.6
1.0
−0.5
0.0
0.5
1.0
Pruning
all
best
chull
mvo
pfront
rand
Count
1
1,767

Suppl. Fig. 5. Boxplot MANOVA
