# Supplementary material for: Unsupervised encoding selection through ensemble pruning for biomedical classification
Source: BioData Min. 2023 Mar 16;16:10. doi: 10.1186/s13040-022-00317-7 (PMC10018861; doi:10.1186/s13040-022-00317-7)

List of encodings

Refer to Spänig *et al.* (2021) for more details (https://doi.org/10.1093/nargab/lqab039).

encoding	params_1	params_2	params_3	params_4
aac				
aaindex	QIAN880101; WOLS870102; RACS820102; RACS820107; VASM830101; RICJ880104; GEOR030103; QIAN880102; FASG760103; AURR980118; GEOR030106; AURR980115; KUMS000103; QIAN880103; ROBB760111; FINA910104; ZIMJ680104; BUNA790102; QIAN880117; KHAG800101; BUNA790103			
apaac	lambda	4; 1; 2; 3; 7; 8; 5; 9; 6		
asa				
binary				
blomap				
blosum62				
cgr	res	20; 100; 200; 10	sf	0.5; 0.8632713
cksaagp	gap	4; 1; 2; 3; 7; 8; 5; 6		
cksaap	gap	4; 1; 2; 3; 7; 8; 5; 6		
ctdc				

**encoding**	**params_1**	**params_2**	**params_3**	**params_4**
ctdd				
ctdt				
ctriad				
dde				
delaunay	number; cartesian; average; total; frequency	instances; distance; product		
disorderb				
disorderc				
dist_freq	dn	50; 100; 10; 5; 20	dc	50; 100; 10; 5; 20
distance	distribution			
dpc				
eaac	window	4; 1; 2; 3; 7; 8; 5; 9; 6		
egaac	window	4; 1; 2; 3; 7; 8; 5; 6		
electrostatic_hull		3; 0; 12; 9; 6		
fft	aaindex	QIAN880101; WOLS870102; RACS820102; RACS820107; VASM830101; RICJ880104; GEOR030103; QIAN880102; FASG760103; AURR980118; GEOR030106; AURR980115; KUMS000103; QIAN880103; ROBB760111; FINA910104; ZIMJ680104; BUNA790102;		

encoding	params_1	params_2	params_3	params_4
		QIAN880117; KHAG800101; BUNA790103		
fldpc	aaindex	QIAN880101; WOLS870102; RACS820102; RACS820107; VASM830101; RICJ880104; GEOR030103; QIAN880102; FASG760103; AURR980118; GEOR030106; AURR980115; KUMS000103; QIAN880103; ROBB760111; FINA910104; ZIMJ680104; BUNA790102; QIAN880117; KHAG800101; BUNA790103		
flgc	aaindex	QIAN880101; WOLS870102; RACS820102; RACS820107; VASM830101; RICJ880104; GEOR030103; QIAN880102; FASG760103; AURR980118; GEOR030106; AURR980115; KUMS000103; QIAN880103; ROBB760111; FINA910104; ZIMJ680104; BUNA790102; QIAN880117;		

encoding	params_1	params_2	params_3	params_4
		KHAG800101; BUNA790103		
gaac				
gdpc				
geary	nlag	4; 1; 2; 3; 7; 8; 5; 9; 6		
gtpc				
ksctriad	gap	3; 1; 2		
moran	nlag	4; 1; 2; 3; 7; 8; 5; 9; 6		
ngram	e2; a3; s3; s2; e3; a2	300; 1; 50; 100; 200; 5; 20		
nmbroto	nlag	4; 1; 2; 3; 7; 8; 5; 9; 6		
paac	lambda	4; 1; 2; 3; 7; 8; 5; 9; 6		
qsar				
qsorder	nlag	4; 1; 2; 3; 7; 8; 5; 9; 6		
socnumber	nlag	4; 1; 2; 3; 7; 8; 5; 9; 6		
sseb				
ssec				
psekraac t1	st-lambda-correlation	rt-10	ktu-3	la-5
psekraac t10	st-g-gap	rt-9	ktu-1	la-2
psekraac t11	st-lambda-correlation	rt-11	ktu-3	la-2
psekraac t12	st-lambda-correlation	rt-7	ktu-1	la-3
psekraac t13	st-lambda-correlation	rt-20	ktu-1	la-3
psekraac t14	st-lambda-	rt-14	ktu-3	la-3

encoding	params_1	params_2	params_3	params_4
	correlation			
psekraac t15	st-lambda-correlation	rt-15	ktu-1	la-3
psekraac t16	st-lambda-correlation	rt-8	ktu-1	la-1
psekraac t2	st-lambda-correlation	rt-2	ktu-1	la-2
psekraac t3A	st-g-gap	rt-9	ktu-2	la-2
t3B	st-g-gap	rt-9	ktu-1	la-2
psekraac t4	st-lambda-correlation	rt-9	ktu-1	la-3
psekraac t5	st-lambda-correlation	rt-15	ktu-1	la-1
psekraac t6A	st-lambda-correlation	rt-20	ktu-1	la-3
psekraac t6B	st-g-gap	rt-5	ktu-3	la-2
psekraac t6C	st-g-gap	rt-5	ktu-3	la-1
psekraac t7	st-g-gap	rt-12	ktu-2	la-3
psekraac t8	st-g-gap	rt-11	ktu-1	la-2
psekraac t9	st-g-gap	rt-13	ktu-3	la-2
ta				
tpc				
waac	aaindex	QIAN880101; WOLS870102; RACS820102; RACS820107; VASM830101; RICJ880104; GEOR030103; QIAN880102; FASG760103; AURR980118; GEOR030106; AURR980115; KUMS000103; QIAN880103;		

encoding	params_1	params_2	params_3	params_4
		ROBB760111; FINA910104; ZIMJ680104; BUNA790102; QIAN880117; KHAG800101; BUNA790103		
zscale				

Statistics

anova_summary_aov

	term	df	sumsq	meansq	statistic	p.value	experiment
**1**	model	3	29.933497	9.977832	1568.461458	0.0	anova_summary_aov
**2**	Residuals	396	2.519170	0.006362	-	-	anova_summary_aov

anova_tukey_hsd

	term	contrast	null.value	estimate	conf.low	conf.high	adj.p.value	experiment
**1**	model	dt-bayes	0	-0.467445	-0.496546	-0.438343	0.000000	anova_tukey_hsd
**2**	model	lr-bayes	0	0.146112	0.117011	0.175213	0.000000	anova_tukey_hsd
**3**	model	rf-bayes	0	-0.460947	-0.490048	-0.431846	0.000000	anova_tukey_hsd
**4**	model	lr-dt	0	0.613557	0.584455	0.642658	0.000000	anova_tukey_hsd
**5**	model	rf-dt	0	0.006498	-0.022603	0.035599	0.939211	anova_tukey_hsd
**6**	model	rf-lr	0	-0.607059	-0.636160	-0.577958	0.000000	anova_tukey_hsd

anova_error_summary_aov

	term	df	sumsq	meansq	statistic	p.value	experiment
**1**	model	4	1311.686823	327.921706	96633.815534	0.0	anova_error_summary_aov
**2**	Residuals	500987	1700.072699	0.003393	-	-	anova_error_summary_aov

anova_error_tukey_hsd

	term	contrast	null.value	estimate	conf.low	conf.high	adj.p.value	experiment
**1**	model	dt-bayes	0	-0.070730	-0.071440	-0.070020	0	anova_error_tukey_hsd
**2**	model	lr-bayes	0	-0.044169	-0.044878	-0.043459	0	anova_error_tukey_hsd
**3**	model	mlp-bayes	0	-0.092753	-0.093463	-0.092043	0	anova_error_tukey_hsd
**4**	model	rf-bayes	0	-0.153858	-0.154568	-0.153148	0	anova_error_tukey_hsd
**5**	model	lr-dt	0	0.026561	0.025851	0.027271	0	anova_error_tuke

	term	contrast	null.value	estimate	conf.low	conf.high	adj.p.value	experiment
								y_hsd
**6**	model	mlp-dt	0	-0.022023	-0.022733	-0.021314	0	anova_error_tukey_hsd
**7**	model	rf-dt	0	-0.083128	-0.083838	-0.082418	0	anova_error_tukey_hsd
**8**	model	mlp-lr	0	-0.048585	-0.049295	-0.047875	0	anova_error_tukey_hsd
**9**	model	rf-lr	0	-0.109689	-0.110399	-0.108979	0	anova_error_tukey_hsd
**10**	model	rf-mlp	0	-0.061105	-0.061815	-0.060395	0	anova_error_tukey_hsd

anova_kappa_summary_aov

	term	df	sumsq	meansq	statistic	p.value	experiment
**1**	model	4	6461.607566	1615.401891	49340.382944	0.0	anova_kappa_summary_aov
**2**	Residuals	500987	16402.291573	0.032740	-	-	anova_kappa_summary_aov

anova_kappa_tukey_hsd

	term	contrast	null.value	estimate	conf.low	conf.high	adj.p.value	experiment
**1**	model	dt-bayes	0	0.101087	0.098882	0.103292	0	anova_kappa_tukey_hsd
**2**	model	lr-bayes	0	0.090764	0.088559	0.092969	0	anova_kappa_tukey_hsd
**3**	model	mlp-bayes	0	0.172824	0.170619	0.175029	0	anova_kappa_tukey_hsd
**4**	model	rf-bayes	0	0.339699	0.337494	0.341904	0	anova_kappa_tukey_hsd
**5**	model	lr-dt	0	-0.010323	-0.012528	-0.008118	0	anova_kappa_tukey_hsd
**6**	model	mlp-dt	0	0.071737	0.069532	0.073942	0	anova_kappa_tukey_hsd
**7**	model	rf-dt	0	0.238612	0.236407	0.240817	0	anova_kappa_tukey_hsd
**8**	model	mlp-lr	0	0.082060	0.079855	0.084265	0	anova_kappa_tukey_hsd
**9**	model	rf-lr	0	0.248935	0.246730	0.251141	0	anova_kappa_tukey_hsd
**10**	model	rf-mlp	0	0.166875	0.164670	0.169080	0	anova_kappa_tukey_hsd

manova_summary

	term	df	pillai	statistic	num.df	den.df	p.value	experiment
**1**	model	4	0.399578	33638.377615	8.0	1077848.0	0.0	manova_summary
**2**	Residuals	538924	-	-	-	-	-	manova_summary

manova_summary_aov

	Df	Sum.Sq	Mean.Sq	F.value	Pr..F.	response	experiment
**model**	4	6482.977465	1620.744366	40454.698437	0.0	Response 1	manova_summary_aov
**Residuals**	538924	21591.015890	0.040063	-	-	Response 1	manova_summary_aov
**model 1**	4	1333.528477	333.382119	81996.319322	0.0	Response 2	manova_summary_aov
**Residuals 1**	538924	2191.166954	0.004066	-	-	Response 2	manova_summary_aov

Plots

Refer to main manuscript for more details.

Suppl. Fig. 1. MVO fitness vs. generations.

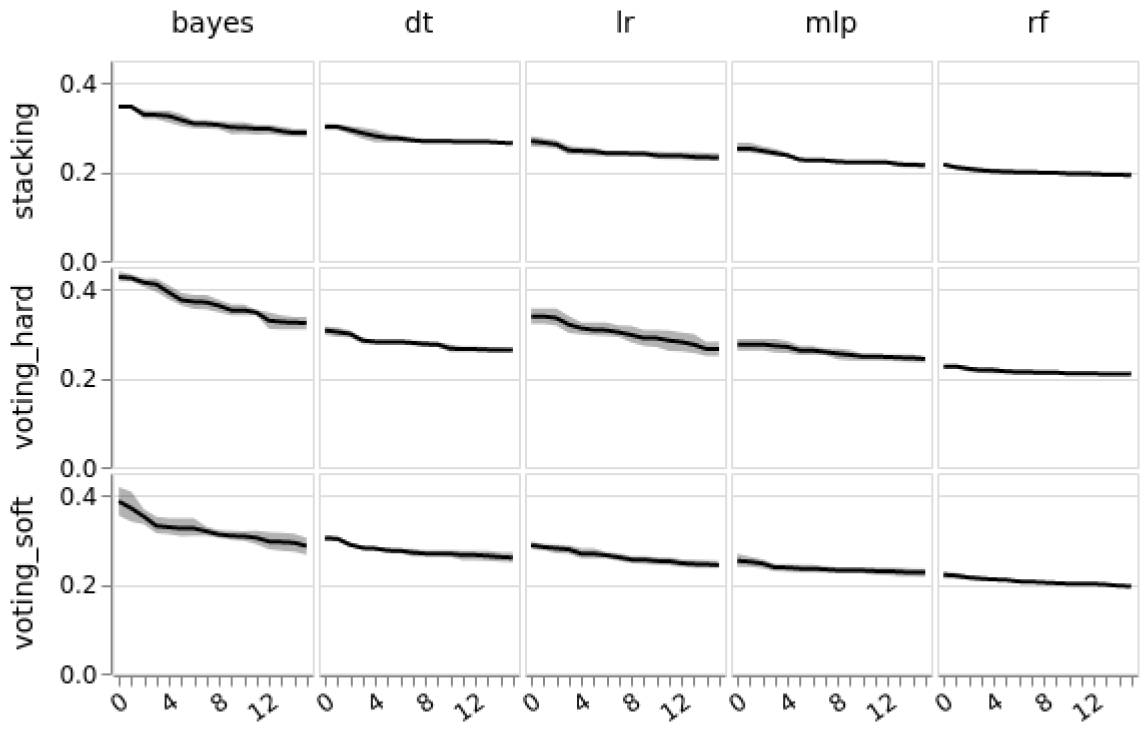

Suppl. Fig. 2. XCD chart

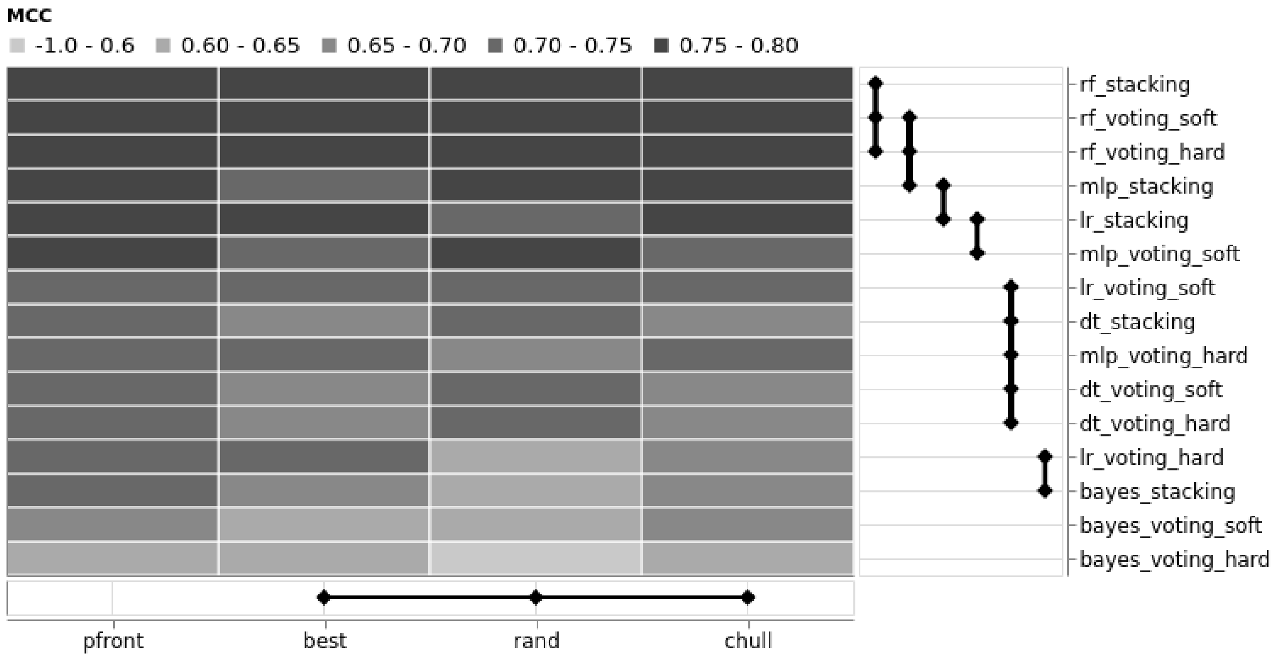

Suppl. Fig. 3. Boxplot

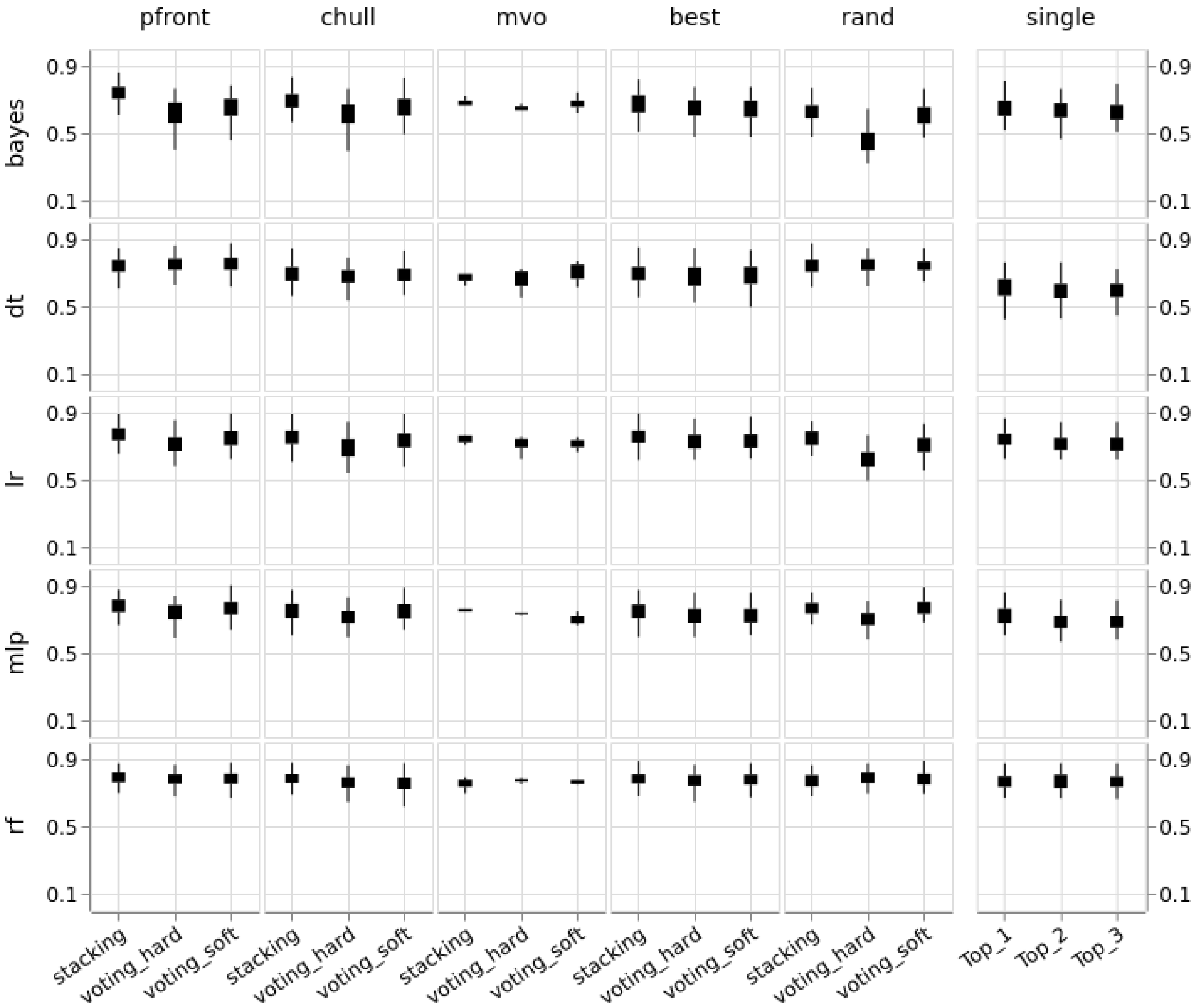

Suppl. Fig. 4. Kappa-error plot

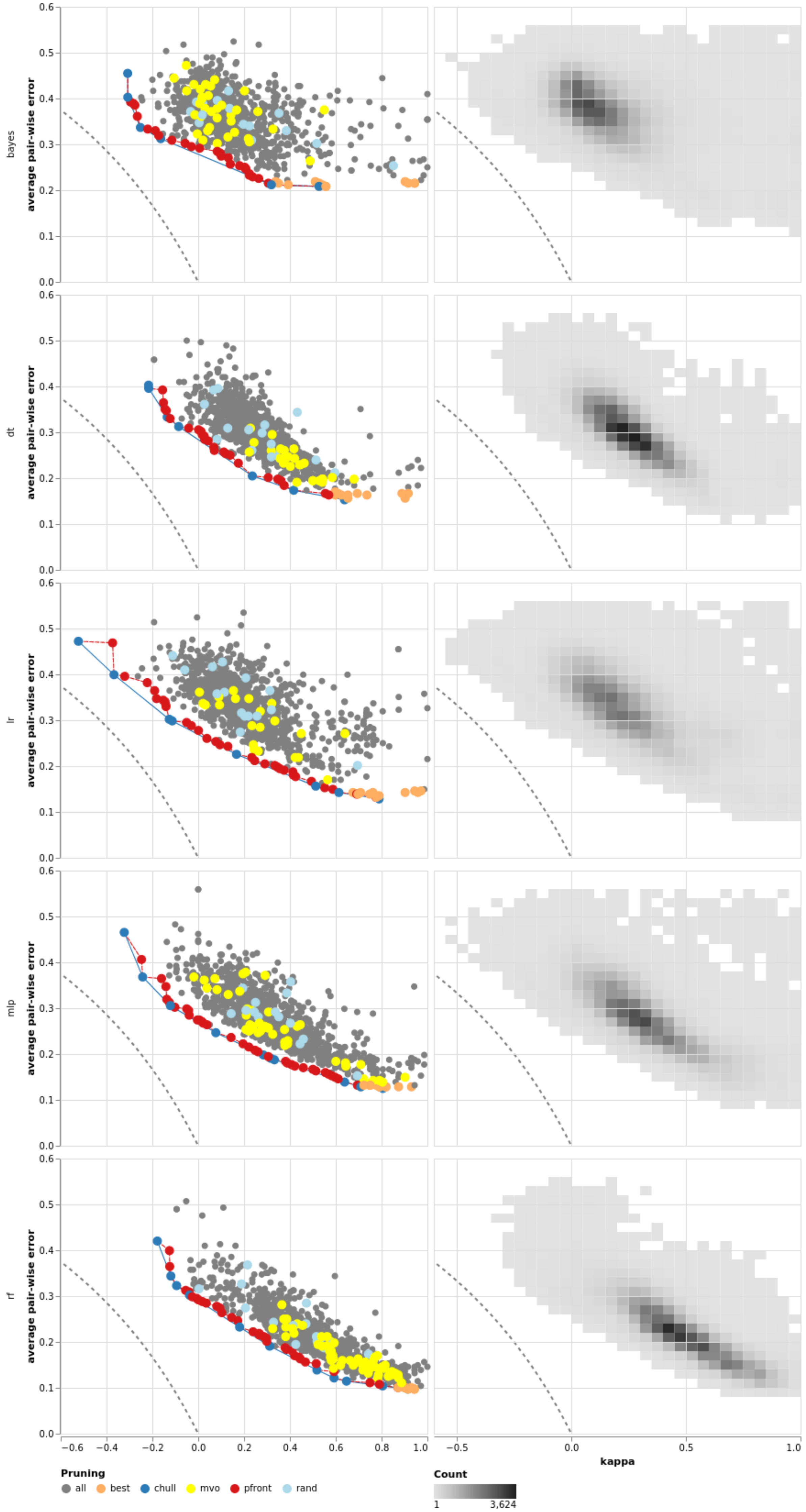

average pair-wise error
bayes
dt
lr
mlp
rf
kappa
−0.6 −0.4 −0.2 0.0 0.2 0.4 0.6 0.8 1.0
−0.5 0.0 0.5 1.0
0.0 0.1 0.2 0.3 0.4 0.5 0.6
Pruning
all best chull mvo pfront rand
Count
1 3,624

Suppl. Fig. 5. Boxplot MANOVA

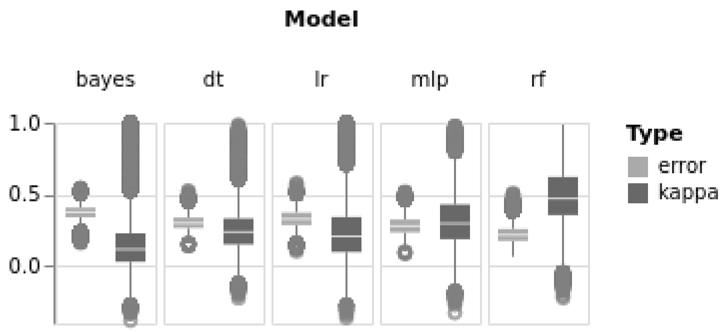

Supplement: Supplementary file 1 — Additional file 1. [file 13040_2022_317_MOESM1_ESM.zip › supplements/avp_amppredR1.pdf]
