# Supplementary material for: Unsupervised encoding selection through ensemble pruning for biomedical classification
Source: BioData Min. 2023 Mar 16;16:10. doi: 10.1186/s13040-022-00317-7 (PMC10018861; doi:10.1186/s13040-022-00317-7)

List of encodings

Refer to Spänig *et al.* (2021) for more details (https://doi.org/10.1093/nargab/lqab039).

encoding	params_1	params_2	params_3	params_4
aac				
aaindex	QIAN880102; QIAN880117; QIAN880101; GEOR030103; RACS820102; KUMS000103; GEOR030106; BUNA790102; AURR980118; ROBB760111; FASG760103; WOLS870102; AURR980115; QIAN880103; KHAG800101; RICJ880104; VASM830101; RACS820107; ZIMJ680104; FINA910104; BUNA790103			
apaac	lambda	4; 2; 3; 1		
asa				
binary				
blomap				
blosum62				
cgr	res	200; 100; 10; 20	sf	0.5; 0.8632713
cksaagp	gap	2; 3; 1		
cksaap	gap	2; 3; 1		
ctdc				
ctdd				
ctdt				

encoding	params_1	params_2	params_3	params_4
ctriad				
dde				
delaunay	total; frequency; cartesian; average; number	instances; distance; product		
disorderb				
disorderc				
dist_freq	dn	100; 5; 20; 50; 10	dc	100; 5; 20; 50; 10
distance	distribution			
dpc				
eaac	window	4; 2; 3; 1		
egaac	window	4; 7; 5; 8; 6; 2; 3; 1		
electrostatic_hull		12; 9; 6; 0; 3		
fft	aaindex	QIAN880102; QIAN880117; QIAN880101; GEOR030103; RACS820102; KUMS000103; GEOR030106; BUNA790102; AURR980118; ROBB760111; FASG760103; WOLS870102; AURR980115; QIAN880103; KHAG800101; RICJ880104; VASM830101; RACS820107; ZIMJ680104; FINA910104; BUNA790103		

encoding	params_1	params_2	params_3	params_4
fldpc	aaindex	QIAN880102; QIAN880117; QIAN880101; GEOR030103; RACS820102; KUMS000103; GEOR030106; BUNA790102; AURR980118; ROBB760111; FASG760103; WOLS870102; AURR980115; QIAN880103; KHAG800101; RICJ880104; VASM830101; RACS820107; ZIMJ680104; FINA910104; BUNA790103		
flgc	aaindex	QIAN880102; QIAN880117; QIAN880101; GEOR030103; RACS820102; KUMS000103; GEOR030106; BUNA790102; AURR980118; ROBB760111; FASG760103; WOLS870102; AURR980115; QIAN880103; KHAG800101; RICJ880104; VASM830101; RACS820107; ZIMJ680104; FINA910104; BUNA790103		

**encoding**	**params_1**	**params_2**	**params_3**	**params_4**
gaac				
gdpc				
geary	nlag	4; 2; 3; 1		
gtpc				
ksctriad	gap	1		
moran	nlag	4; 2; 3; 1		
ngram	a3; e3; s3; s2; a2; e2	100; 5; 20; 200; 300; 50; 1		
nmbroto	nlag	4; 2; 3; 1		
paac	lambda	4; 2; 3; 1		
qsar				
qsorder	nlag	4; 2; 3; 1		
socnumber	nlag	4; 2; 3; 1		
sseb				
ssec				
psekraac t1	st-g-gap	rt-10	ktu-1	la-5
psekraac t10	st-g-gap	rt-8	ktu-1	la-1
psekraac t11	st-g-gap	rt-11	ktu-1	la-1
psekraac t12	st-lambda-correlation	rt-12	ktu-1	la-3
psekraac t13	st-g-gap	rt-20	ktu-1	la-3
psekraac t14	st-g-gap	rt-10	ktu-1	la-1
psekraac t15	st-g-gap	rt-10	ktu-1	la-1
psekraac t16	st-g-gap	rt-9	ktu-1	la-3
psekraac t2	st-g-gap	rt-5	ktu-1	la-2
psekraac t3A	st-g-gap	rt-12	ktu-3	la-3
psekraac t3B	st-lambda-correlation	rt-9	ktu-3	la-3
psekraac t4	st-g-gap	rt-5	ktu-1	la-3
psekraac t5	st-g-gap	rt-8	ktu-1	la-3

encoding	params_1	params_2	params_3	params_4
psekraac t6A	st-g-gap	rt-5	ktu-1	la-3
psekraac t6B	st-lambda-correlation	rt-5	ktu-1	la-3
psekraac t6C	st-g-gap	rt-5	ktu-2	la-1
psekraac t7	st-lambda-correlation	rt-10	ktu-1	la-1
psekraac t8	st-g-gap	rt-2	ktu-2	la-1
psekraac t9	st-g-gap	rt-13	ktu-3	la-3
ta				
tpc				
waac	aaindex	QIAN880102; QIAN880117; QIAN880101; GEOR030103; RACS820102; KUMS000103; GEOR030106; BUNA790102; AURR980118; ROBB760111; FASG760103; WOLS870102; AURR980115; QIAN880103; KHAG800101; RICJ880104; VASM830101; RACS820107; ZIMJ680104; FINA910104; BUNA790103		
zscale				

Statistics

anova_summary_aov

	term	df	sumsq	meansq	statistic	p.value	experiment
**1**	model	3	13.556544	4.518848	606.44333	0.0	anova_summary_aov
**2**	Residuals	396	2.950752	0.007451	-	-	anova_summary_aov

anova_tukey_hsd

	term	contrast	null.value	estimate	conf.low	conf.high	adj.p.value	experiment
**1**	model	dt-bayes	0	-0.443736	-0.475232	-0.412241	0.000000	anova_tukey_hsd
**2**	model	lr-bayes	0	-0.423930	-0.455426	-0.392435	0.000000	anova_tukey_hsd
**3**	model	rf-bayes	0	-0.404085	-0.435580	-0.372589	0.000000	anova_tukey_hsd
**4**	model	lr-dt	0	0.019806	-0.011690	0.051301	0.367140	anova_tukey_hsd
**5**	model	rf-dt	0	0.039651	0.008156	0.071147	0.006885	anova_tukey_hsd
**6**	model	rf-lr	0	0.019846	-0.011650	0.051341	0.365325	anova_tukey_hsd

anova_error_summary_aov

	term	df	sumsq	meansq	statistic	p.value	experiment
**1**	model	4	461.191596	115.297899	36960.388758	0.0	anova_error_summary_aov
**2**	Residuals	500995	1562.853447	0.003119	-	-	anova_error_summary_aov

anova_error_tukey_hsd

	term	contrast	null.value	estimate	conf.low	conf.high	adj.p.value	experiment
**1**	model	dt-bayes	0	0.000582	-0.000099	0.001263	0.134694	anova_error_tukey_hsd
**2**	model	lr-bayes	0	-0.031516	-0.032196	-0.030835	0.000000	anova_error_tukey_hsd
**3**	model	mlp-bayes	0	-0.051940	-0.052621	-0.051259	0.000000	anova_error_tukey_hsd
**4**	model	rf-bayes	0	-0.078042	-0.078723	-0.077362	0.000000	anova_error_tukey_hsd
**5**	model	lr-dt	0	-0.032097	-0.032778	-0.031417	0.000000	anova_error_tukey_hsd
**6**	model	mlp-dt	0	-0.052522	-0.053203	-0.051841	0.000000	anova_error_tukey_hsd
**7**	model	rf-dt	0	-0.078624	-0.079305	-0.077944	0.000000	anova_error_tukey_hsd
**8**	model	mlp-lr	0	-0.020425	-0.021105	-0.019744	0.000000	anova_error_tukey_hsd
**9**	model	rf-lr	0	-0.046527	-0.047208	-0.045846	0.000000	anova_error_tukey_hsd
**10**	model	rf-mlp	0	-0.026102	-0.026783	-0.025422	0.000000	anova_error_tukey_hsd

anova_kappa_summary_aov

	term	df	sumsq	meansq	statistic	p.value	experiment
**1**	model	4	3195.780821	798.945205	17167.446254	0.0	anova_kappa_summary_aov
**2**	Residuals	500995	23315.497672	0.046538	-	-	anova_kappa_summary_aov

anova_kappa_tukey_hsd

	term	contrast	null.value	estimate	conf.low	conf.high	adj.p.value	experiment
**1**	model	dt-bayes	0	-0.042441	-0.045070	-0.039812	0	anova_kappa_tukey_hsd
**2**	model	lr-bayes	0	0.048334	0.045705	0.050962	0	anova_kappa_tukey_hsd
**3**	model	mlp-bayes	0	0.106551	0.103922	0.109181	0	anova_kappa_tukey_hsd
**4**	model	rf-bayes	0	0.184504	0.181875	0.187134	0	anova_kappa_tukey_hsd
**5**	model	lr-dt	0	0.090774	0.088146	0.093403	0	anova_kappa_tukey_hsd
**6**	model	mlp-dt	0	0.148992	0.146363	0.151621	0	anova_kappa_tukey_hsd
**7**	model	rf-dt	0	0.226945	0.224316	0.229574	0	anova_kappa_tukey_hsd
**8**	model	mlp-lr	0	0.058218	0.055589	0.060847	0	anova_kappa_tukey_hsd
**9**	model	rf-lr	0	0.136171	0.133542	0.138800	0	anova_kappa_tukey_hsd
**10**	model	rf-mlp	0	0.077953	0.075324	0.080582	0	anova_kappa_tukey_hsd

manova_summary

	term	df	pillai	statistic	num.df	den.df	p.value	experiment
**1**	model	4	0.217148	16394.804684	8.0	1076852.0	0.0	manova_summary
**2**	Residuals	538426	-	-	-	-	-	manova_summary

manova_summary_aov

	Df	Sum.Sq	Mean.Sq	F.value	Pr..F.	response	experiment
**model**	4	3243.561546	810.890387	15685.572677	0.0	Response 1	manova_summary_aov
**Residuals**	538426	27834.780167	0.051697	-	-	Response 1	manova_summary_aov
**model 1**	4	479.677592	119.919398	34195.893764	0.0	Response 2	manova_summary_aov
**Residuals 1**	538426	1888.171786	0.003507	-	-	Response 2	manova_summary_aov

Plots

Refer to main manuscript for more details.

Suppl. Fig. 1. MVO fitness vs. generations.

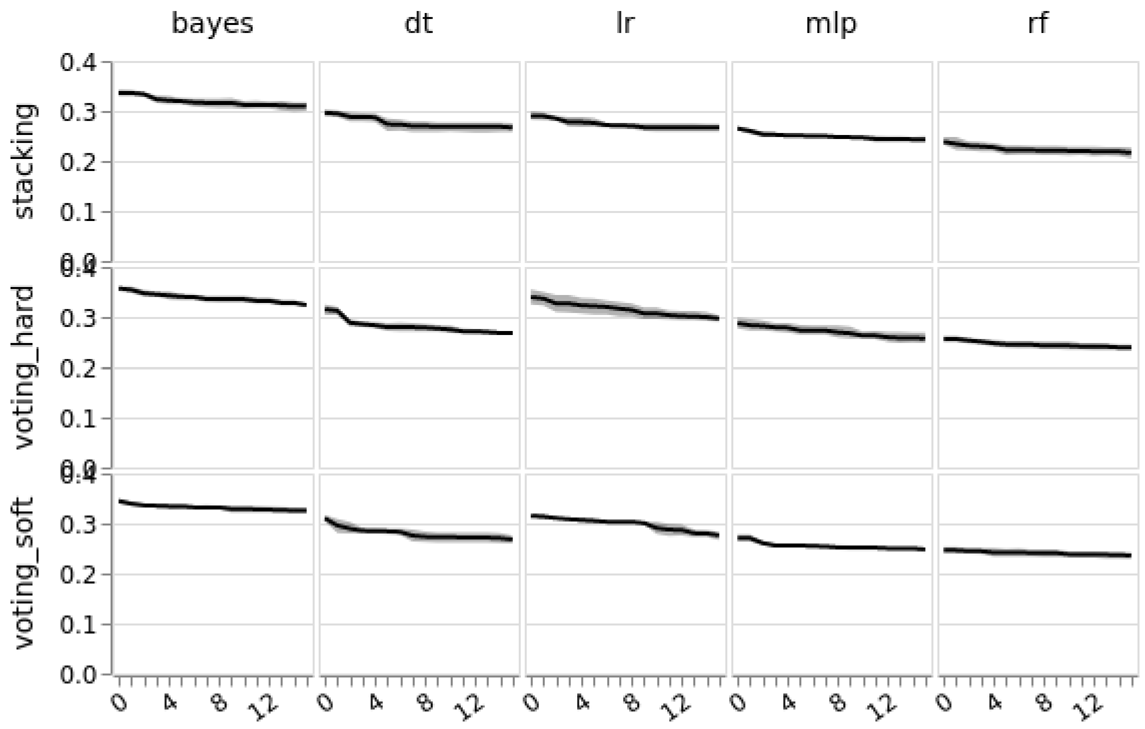

Suppl. Fig. 2. XCD chart

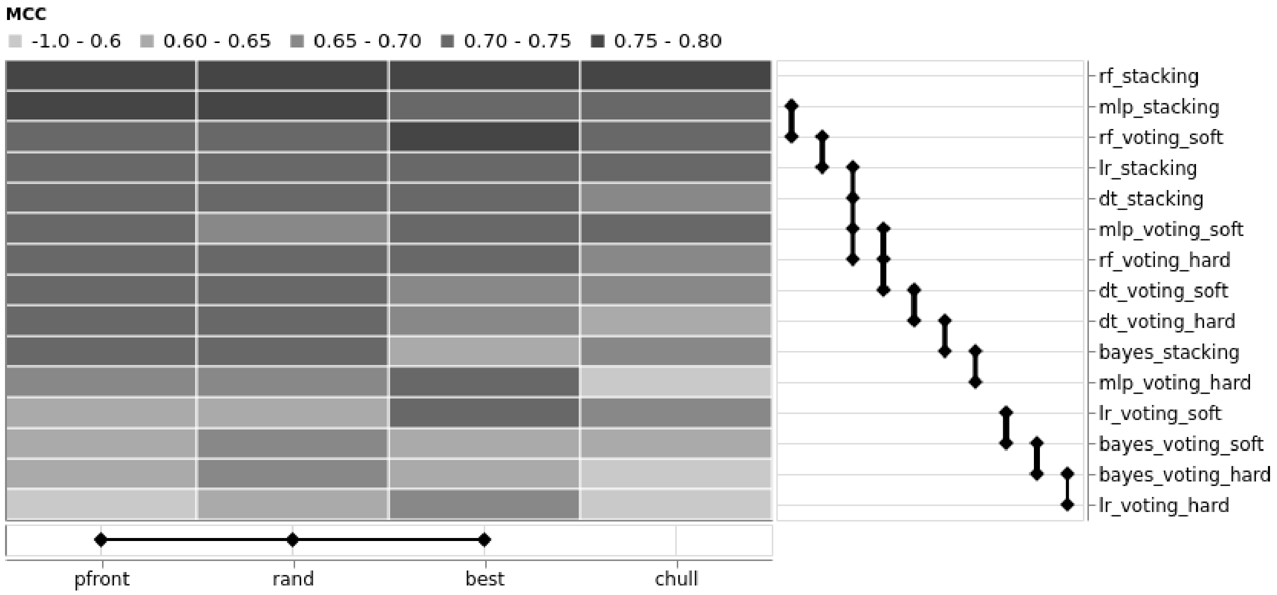

Suppl. Fig. 3. Boxplot

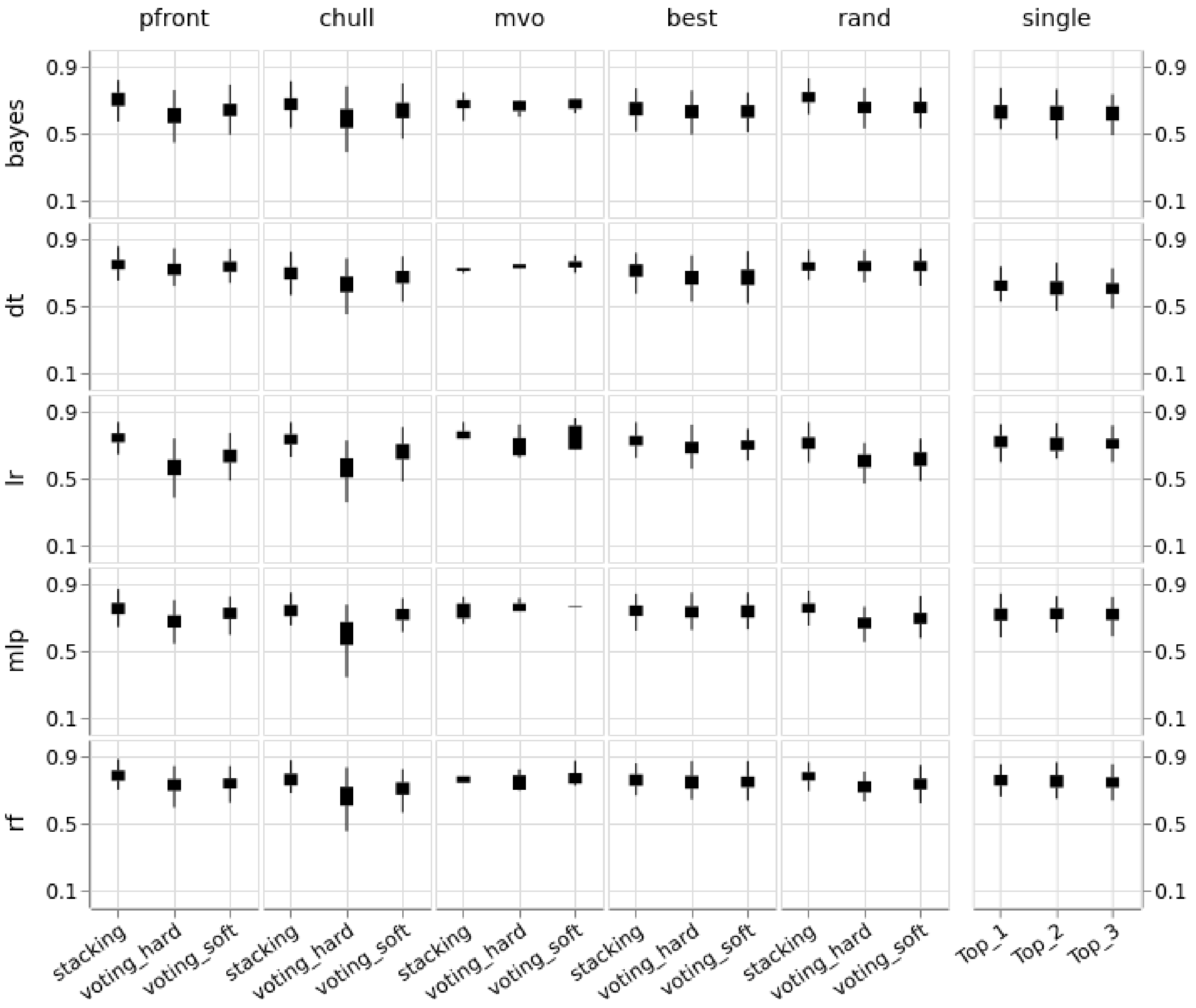

Suppl. Fig. 4. Kappa-error plot

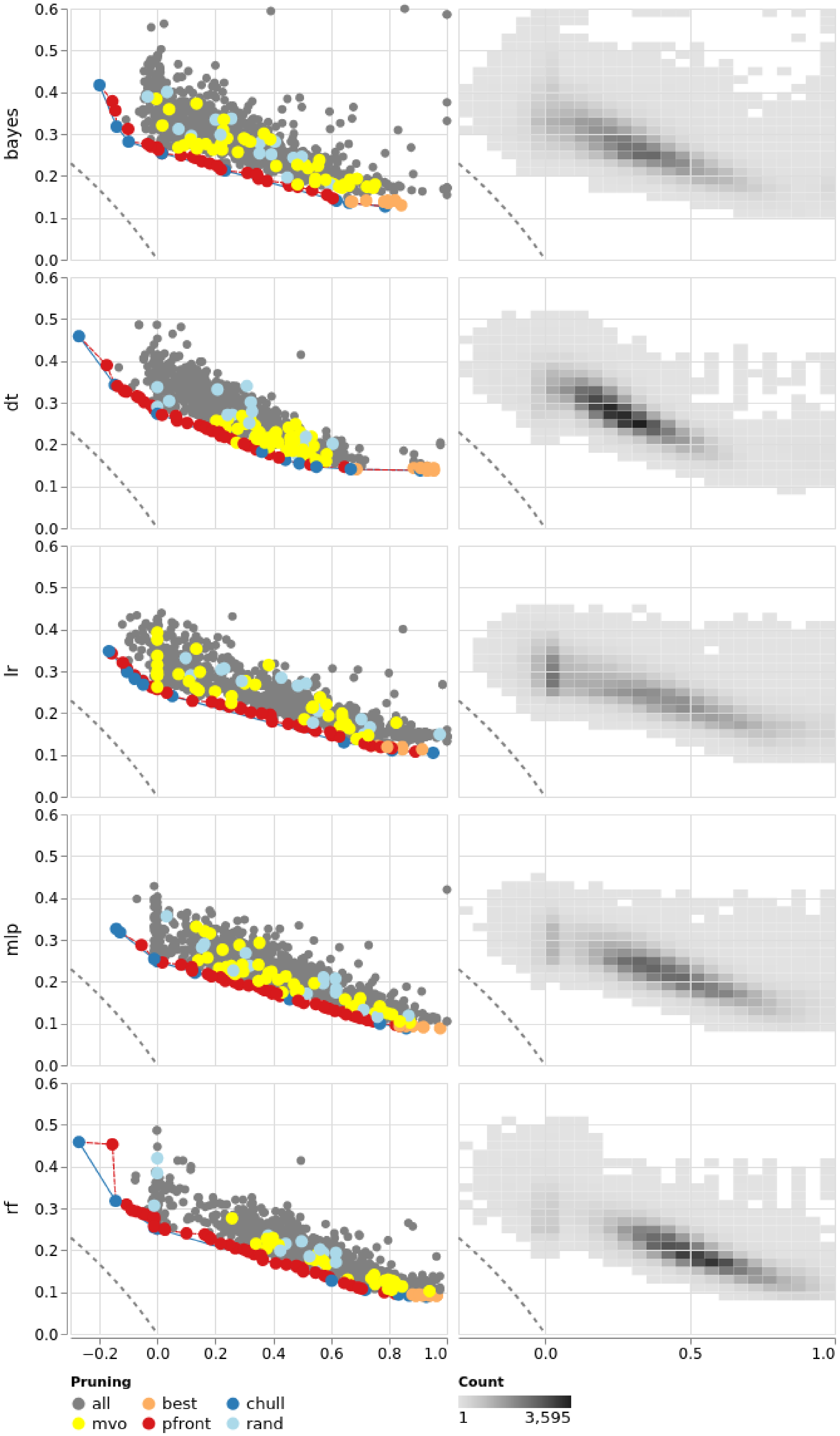

bayes
dt
lr
mlp
rf
0.0
0.1
0.2
0.3
0.4
0.5
0.6
−0.2
0.0
0.2
0.4
0.6
0.8
1.0
0.5
Pruning
all
best
chull
mvo
pfront
rand
Count
1
3,595

Suppl. Fig. 5. Boxplot MANOVA

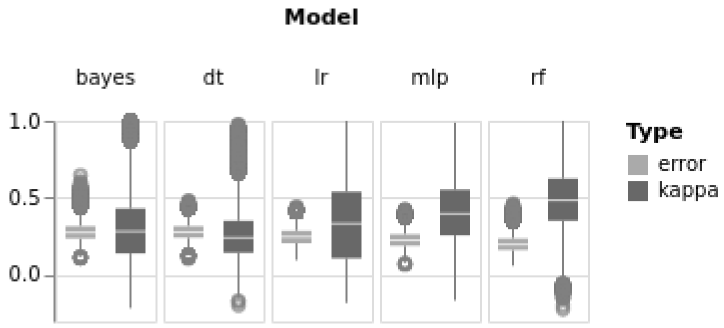

Supplement: Supplementary file 1 — Additional file 1. [file 13040_2022_317_MOESM1_ESM.zip › supplements/cpp_mlcppR1.pdf]
