# Supplementary material for: Unsupervised encoding selection through ensemble pruning for biomedical classification
Source: BioData Min. 2023 Mar 16;16:10. doi: 10.1186/s13040-022-00317-7 (PMC10018861; doi:10.1186/s13040-022-00317-7)

List of encodings

Refer to Spänig *et al.* (2021) for more details (https://doi.org/10.1093/nargab/lqab039).

**encoding**	**params_1**	**params_2**	**params_3**	**params_4**
aac				
aaindex	ZIMJ680104; QIAN880101; FASG760103; QIAN880102; GEOR030106; RACS820107; ROBB760111; QIAN880103; GEOR030103; RICJ880104; BUNA790102; QIAN880117; VASM830101; WOLS870102; KUMS000103; AURR980115; BUNA790103; KHAG800101; FINA910104; AURR980118; RACS820102			
apaac	lambda	3; 2; 1		
asa				
binary				
blomap				
blosum62				
cgr	res	100; 200; 20; 10	sf	0.8632713; 0.5
cksaagp	gap	2; 1		
cksaap	gap	2; 1		
ctdc				
ctdd				
ctdt				
ctriad				
dde				
delaunay	average; frequency; total; cartesian; number	distance; instances; product		
disorderb				
disorderc				
dist_freq	dn	100; 20; 5; 50;	dc	100; 20; 5; 50;

**encoding**	**params_1**	**params_2**	**params_3**	**params_4**
		10		10
distance	distribution			
dpc				
eaac	window	3; 2; 1		
egaac	window	2; 1; 8; 3; 6; 5; 4; 7		
electrostatic_hull		3; 6; 9; 0; 12		
fft	aaindex	ZIMJ680104; QIAN880101; FASG760103; QIAN880102; GEOR030106; RACS820107; ROBB760111; QIAN880103; GEOR030103; RICJ880104; BUNA790102; QIAN880117; VASM830101; WOLS870102; KUMS000103; AURR980115; BUNA790103; KHAG800101; FINA910104; AURR980118; RACS820102		
fldpc	aaindex	ZIMJ680104; QIAN880101; FASG760103; QIAN880102; GEOR030106; RACS820107; ROBB760111; QIAN880103; GEOR030103; RICJ880104; BUNA790102; QIAN880117; VASM830101; WOLS870102; KUMS000103; AURR980115; BUNA790103; KHAG800101; FINA910104; AURR980118; RACS820102		

encoding	params_1	params_2	params_3	params_4
flgc	aaindex	ZIMJ680104; QIAN880101; FASG760103; QIAN880102; GEOR030106; RACS820107; ROBB760111; QIAN880103; GEOR030103; RICJ880104; BUNA790102; QIAN880117; VASM830101; WOLS870102; KUMS000103; AURR980115; BUNA790103; KHAG800101; FINA910104; AURR980118; RACS820102		
gaac				
gdpc				
geary	nlag	3; 2; 1		
gtpc				
moran	nlag	3; 2; 1		
ngram	e3; s3; s2; a2; a3; e2	100; 20; 1; 5; 300; 50; 200		
nmbroto	nlag	3; 2; 1		
paac	lambda	3; 2; 1		
qsar				
qsorder	nlag	3; 2; 1		
socnumber	nlag	3; 2; 1		
sseb				
ssec				
psekraac t1	st-lambda-correlation	rt-7	ktu-3	la-6
psekraac t10	st-g-gap	rt-9	ktu-1	la-1
psekraac t11	st-lambda-correlation	rt-7	ktu-3	la-3
psekraac t12	st-lambda-correlation	rt-8	ktu-1	la-1
psekraac t13	st-lambda-correlation	rt-12	ktu-1	la-3
psekraac t14	st-g-gap	rt-18	ktu-3	la-3
psekraac t15	st-lambda-correlation	rt-15	ktu-1	la-2

**encoding**	**params_1**	**params_2**	**params_3**	**params_4**
psekraac t16	st-g-gap	rt-10	ktu-1	la-1
psekraac t2	st-lambda-correlation	rt-2	ktu-1	la-1
psekraac t3A	st-lambda-correlation	rt-15	ktu-3	la-3
psekraac t3B	st-g-gap	rt-2	ktu-1	la-3
psekraac t4	st-g-gap	rt-9	ktu-1	la-1
psekraac t5	st-g-gap	rt-10	ktu-1	la-3
psekraac t6A	st-lambda-correlation	rt-4	ktu-1	la-2
psekraac t6B	st-g-gap	rt-5	ktu-1	la-3
psekraac t6C	st-g-gap	rt-5	ktu-1	la-1
psekraac t7	st-lambda-correlation	rt-10	ktu-1	la-3
psekraac t8	st-g-gap	rt-18	ktu-3	la-3
psekraac t9	st-lambda-correlation	rt-6	ktu-2	la-2
ta				
tpc				
waac	aaindex	ZIMJ680104; QIAN880101; FASG760103; QIAN880102; GEOR030106; RACS820107; ROBB760111; QIAN880103; GEOR030103; RICJ880104; BUNA790102; QIAN880117; VASM830101; WOLS870102; KUMS000103; AURR980115; BUNA790103; KHAG800101; FINA910104; AURR980118; RACS820102		
zscale				

Statistics

anova_summary_aov

	term	df	sumsq	meansq	statistic	p.value	experiment
**1**	model	3	14.826214	4.942071	320.394194	0.0	anova_summary_aov
**2**	Residuals	396	6.108289	0.015425	-	-	anova_summary_aov

anova_tukey_hsd

	term	contrast	null.value	estimate	conf.low	conf.high	adj.p.value	experiment
**1**	model	dt-bayes	0	-0.443834	-0.489149	-0.398519	0.000000	anova_tukey_hsd
**2**	model	lr-bayes	0	-0.140196	-0.185511	-0.094881	0.000000	anova_tukey_hsd
**3**	model	rf-bayes	0	-0.440483	-0.485798	-0.395168	0.000000	anova_tukey_hsd
**4**	model	lr-dt	0	0.303638	0.258323	0.348953	0.000000	anova_tukey_hsd
**5**	model	rf-dt	0	0.003352	-0.041963	0.048667	0.997534	anova_tukey_hsd
**6**	model	rf-lr	0	-0.300287	-0.345602	-0.254972	0.000000	anova_tukey_hsd

anova_error_summary_aov

	term	df	sumsq	meansq	statistic	p.value	experiment
**1**	model	4	830.464557	207.616139	30247.205993	0.0	anova_error_summary_aov
**2**	Residuals	500943	3438.461444	0.006864	-	-	anova_error_summary_aov

anova_error_tukey_hsd

	term	contrast	null.value	estimate	conf.low	conf.high	adj.p.value	experiment
**1**	model	dt-bayes	0	-0.055168	-0.056177	-0.054158	0	anova_error_tukey_hsd
**2**	model	lr-bayes	0	-0.050514	-0.051524	-0.049505	0	anova_error_tukey_hsd
**3**	model	mlp-bayes	0	-0.081826	-0.082836	-0.080816	0	anova_error_tukey_hsd
**4**	model	rf-bayes	0	-0.124231	-0.125240	-0.123221	0	anova_error_tukey_hsd
**5**	model	lr-dt	0	0.004653	0.003644	0.005663	0	anova_error_tukey_hsd
**6**	model	mlp-dt	0	-0.026658	-0.027668	-0.025648	0	anova_error_tukey_hsd
**7**	model	rf-dt	0	-0.069063	-0.070073	-0.068053	0	anova_error_tukey_hsd
**8**	model	mlp-lr	0	-0.031311	-0.032321	-0.030302	0	anova_error_tukey_hsd
**9**	model	rf-lr	0	-0.073716	-0.074726	-0.072707	0	anova_error_tukey_hsd
**10**	model	rf-mlp	0	-0.042405	-0.043415	-0.041395	0	anova_error_tukey_hsd

anova_kappa_summary_aov

	term	df	sumsq	meansq	statistic	p.value	experiment
**1**	model	4	4908.932738	1227.233185	21821.10192	0.0	anova_kappa_summary_aov
**2**	Residuals	500943	28173.365188	0.056241	-	-	anova_kappa_summary_aov

anova_kappa_tukey_hsd

	term	contrast	null.value	estimate	conf.low	conf.high	adj.p.value	experiment
**1**	model	dt-bayes	0	0.109056	0.106166	0.111946	0	anova_kappa_tukey_hsd
**2**	model	lr-bayes	0	0.091623	0.088732	0.094513	0	anova_kappa_tukey_hsd
**3**	model	mlp-bayes	0	0.167191	0.164301	0.170082	0	anova_kappa_tukey_hsd
**4**	model	rf-bayes	0	0.299872	0.296982	0.302762	0	anova_kappa_tukey_hsd
**5**	model	lr-dt	0	-0.017433	-0.020323	-0.014543	0	anova_kappa_tukey_hsd
**6**	model	mlp-dt	0	0.058136	0.055245	0.061026	0	anova_kappa_tukey_hsd
**7**	model	rf-dt	0	0.190816	0.187926	0.193706	0	anova_kappa_tukey_hsd
**8**	model	mlp-lr	0	0.075569	0.072678	0.078459	0	anova_kappa_tukey_hsd
**9**	model	rf-lr	0	0.208249	0.205359	0.211139	0	anova_kappa_tukey_hsd
**10**	model	rf-mlp	0	0.132680	0.129790	0.135571	0	anova_kappa_tukey_hsd

manova_summary

	term	df	pillai	statistic	num.df	den.df	p.value	experiment
**1**	model	4	0.179006	13304.336589	8.0	1082742.0	0.0	manova_summary
**2**	Residuals	541371	-	-	-	-	-	manova_summary

manova_summary_aov

	Df	Sum.Sq	Mean.Sq	F.value	Pr..F.	response	experiment
**model**	4	4907.154017	1226.788504	19395.897198	0.0	Response 1	manova_summary_aov
**Residuals**	541371	34241.660110	0.063250	-	-	Response 1	manova_summary_aov
**model 1**	4	830.497219	207.624305	27441.903908	0.0	Response 2	manova_summary_aov
**Residuals 1**	541371	4095.990492	0.007566	-	-	Response 2	manova_summary_aov

Plots

Refer to main manuscript for more details.

Suppl. Fig. 1. MVO fitness vs. generations.

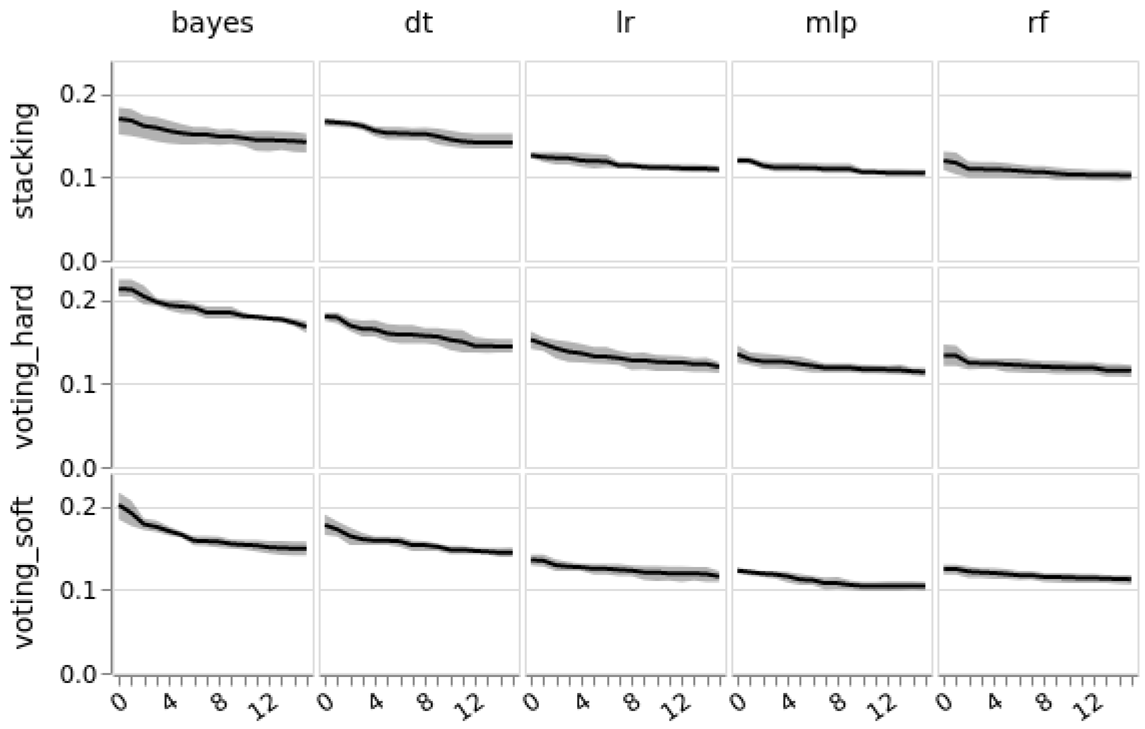

Suppl. Fig. 2. XCD chart

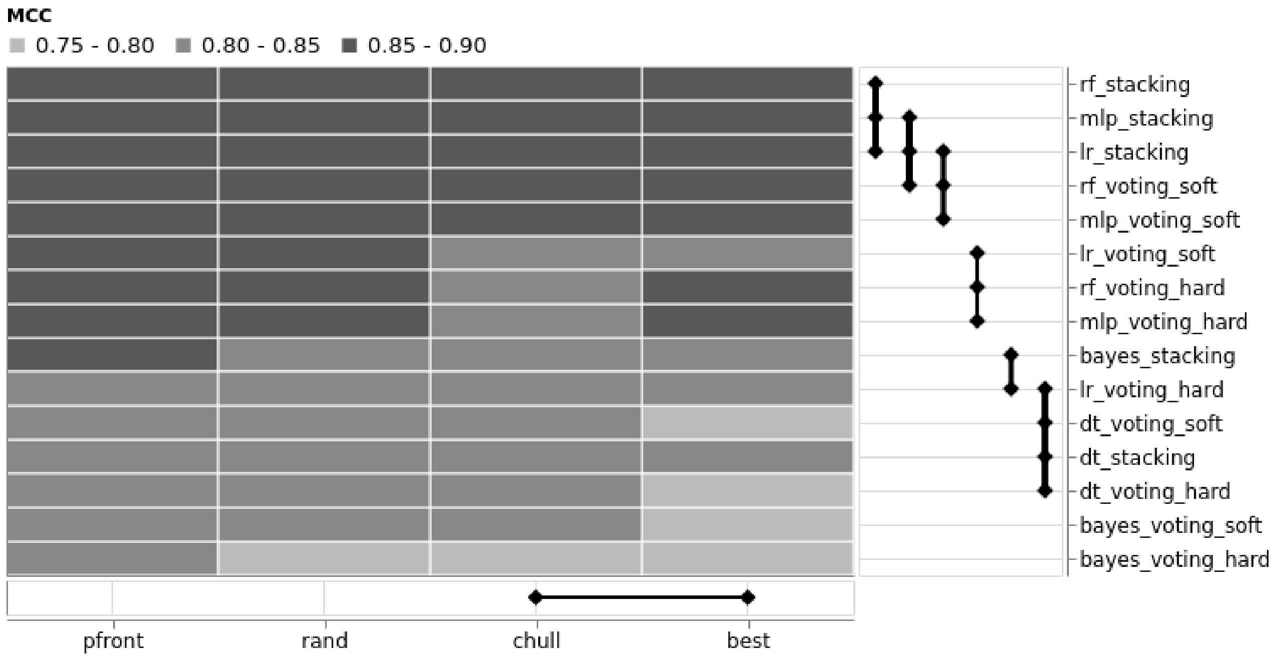

Suppl. Fig. 3. Boxplot

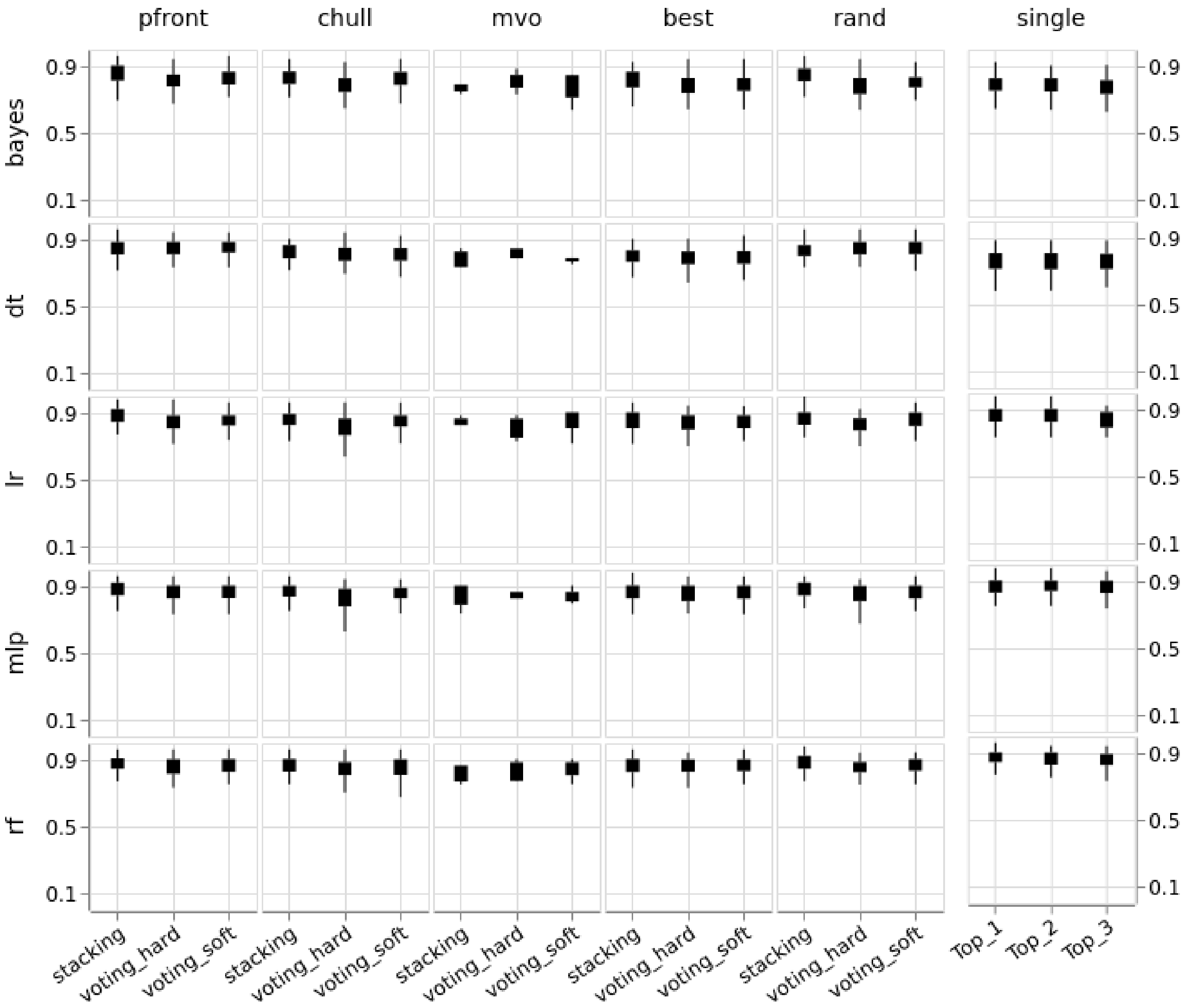

Suppl. Fig. 4. Kappa-error plot

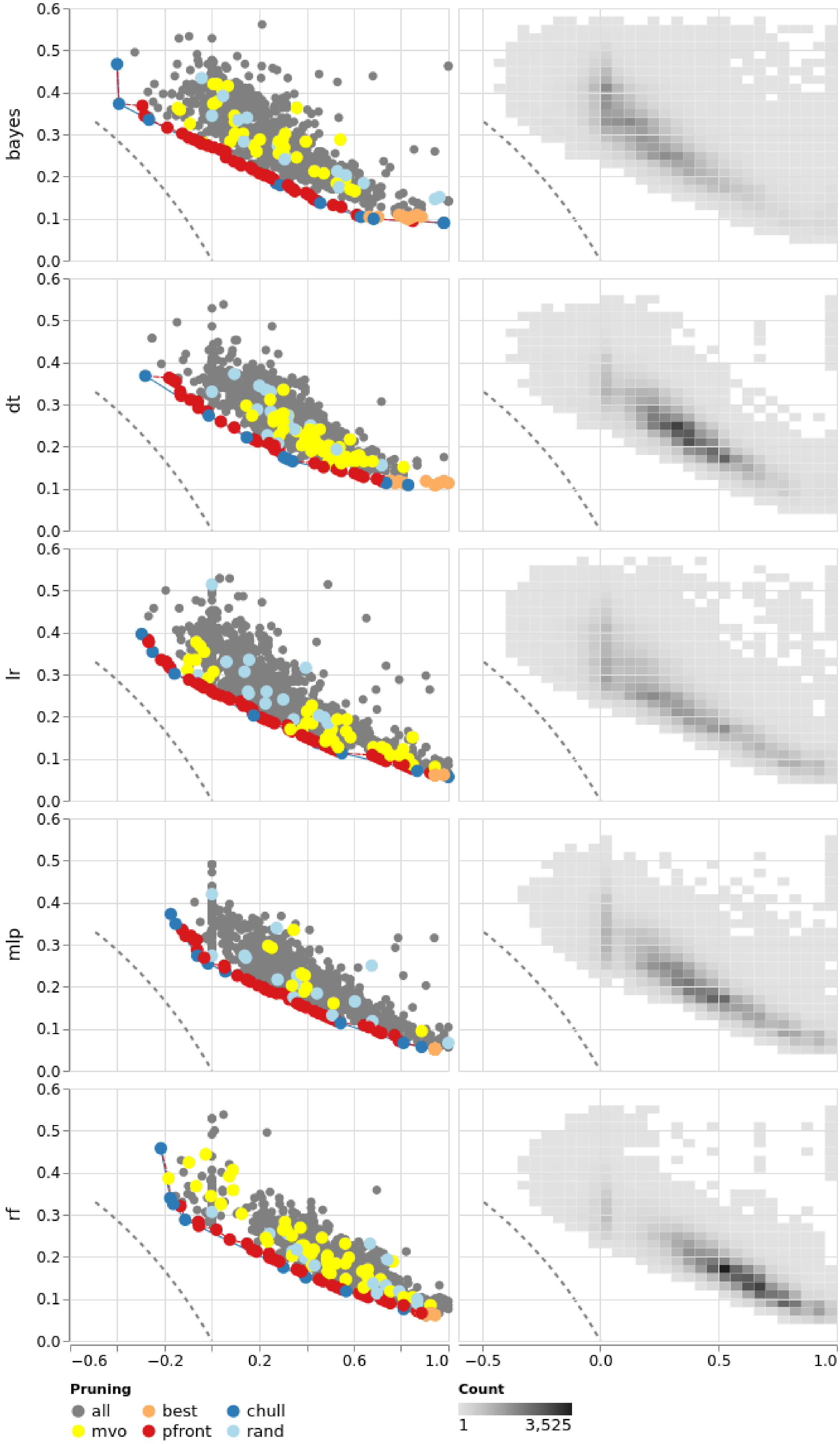
bayes
dt
lr
mlp
rf
0.0
0.1
0.2
0.3
0.4
0.5
0.6
−0.6
−0.2
0.2
0.6
1.0
−0.5
0.0
0.5
1.0
Pruning
all
best
chull
mvo
pfront
rand
Count
1
3,525

Suppl. Fig. 5. Boxplot MANOVA

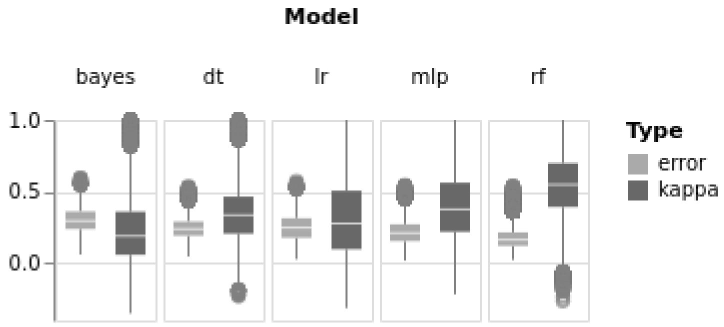

Supplement: Supplementary file 1 — Additional file 1. [file 13040_2022_317_MOESM1_ESM.zip › supplements/hem_hemopiR1.pdf]
