# Supplementary material for: Unsupervised encoding selection through ensemble pruning for biomedical classification
Source: BioData Min. 2023 Mar 16;16:10. doi: 10.1186/s13040-022-00317-7 (PMC10018861; doi:10.1186/s13040-022-00317-7)

List of encodings

Refer to Spänig *et al.* (2021) for more details (https://doi.org/10.1093/nargab/lqab039).

**encoding**	**params_1**	**params_2**	**params_3**	**params_4**
aac				
aaindex	AURR980118; WOLS870102; RICJ880104; KUMS000103; AURR980115; QIAN880117; FASG760103; GEOR030103; FINA910104; KHAG800101; GEOR030106; RACS820102; QIAN880103; QIAN880101; BUNA790103; BUNA790102; QIAN880102; VASM830101; ROBB760111; ZIMJ680104; RACS820107			
apaac	lambda	4; 2; 6; 7; 3; 5; 1		
asa				
binary				
blomap				
blosum62				
cgr	res	200; 10; 20; 100	sf	0.5; 0.8632713
cksaagp	gap	4; 2; 6; 3; 5; 1		
cksaap	gap	4; 2; 6; 3; 5; 1		
ctdc				
ctdd				

encoding	params_1	params_2	params_3	params_4
ctdt				
ctriad				
dde				
delaunay	total; average; cartesian; number; frequency	instances; product; distance		
disorderb				
disorderc				
dist_freq	dn	50; 10; 20; 5; 100	dc	50; 10; 20; 5; 100
distance	distribution			
dpc				
eaac	window	4; 2; 6; 7; 3; 5; 1		
egaac	window	4; 8; 2; 6; 7; 3; 5; 1		
electrostatic_hull		9; 6; 3; 0; 12		
fft	aaindex	AURR980118; WOLS870102; RICJ880104; KUMS000103; AURR980115; QIAN880117; FASG760103; GEOR030103; FINA910104; KHAG800101; GEOR030106; RACS820102; QIAN880103; QIAN880101; BUNA790103; BUNA790102; QIAN880102; VASM830101; ROBB760111; ZIMJ680104;		

encoding	params_1	params_2	params_3	params_4
		RACS820107		
fldpc	aaindex	AURR980118; WOLS870102; RICJ880104; KUMS000103; AURR980115; QIAN880117; FASG760103; GEOR030103; FINA910104; KHAG800101; GEOR030106; RACS820102; QIAN880103; QIAN880101; BUNA790103; BUNA790102; QIAN880102; VASM830101; ROBB760111; ZIMJ680104; RACS820107		
flgc	aaindex	AURR980118; WOLS870102; RICJ880104; KUMS000103; AURR980115; QIAN880117; FASG760103; GEOR030103; FINA910104; KHAG800101; GEOR030106; RACS820102; QIAN880103; QIAN880101; BUNA790103; BUNA790102; QIAN880102; VASM830101; ROBB760111; ZIMJ680104;		

encoding	params_1	params_2	params_3	params_4
		RACS820107		
gaac				
gdpc				
geary	nlag	4; 2; 6; 7; 3; 5; 1		
gtpc				
ksctriad	gap	2; 1		
moran	nlag	4; 2; 6; 7; 3; 5; 1		
ngram	s3; e3; e2; s2; a2; a3	50; 20; 5; 200; 1; 100; 300		
nmbroto	nlag	4; 2; 6; 7; 3; 5; 1		
paac	lambda	4; 2; 6; 7; 3; 5; 1		
qsar				
qsorder	nlag	4; 2; 6; 7; 3; 5; 1		
socnumber	nlag	4; 2; 6; 7; 3; 5; 1		
sseb				
ssec				
psekraac t1	st-lambda-correlation	rt-10	ktu-3	la-2
psekraac t10	st-lambda-correlation	rt-12	ktu-1	la-3
psekraac t11	st-lambda-correlation	rt-9	ktu-3	la-2
psekraac t12	st-lambda-correlation	rt-8	ktu-1	la-1
psekraac t13	st-lambda-correlation	rt-12	ktu-1	la-2
psekraac t14	st-g-gap	rt-10	ktu-1	la-1

encoding	params_1	params_2	params_3	params_4
psekraac t15	st-lambda-correlation	rt-13	ktu-1	la-3
psekraac t16	st-g-gap	rt-10	ktu-1	la-3
psekraac t2	st-lambda-correlation	rt-8	ktu-1	la-1
psekraac t3A	st-g-gap	rt-20	ktu-2	la-1
psekraac t3B	st-g-gap	rt-11	ktu-3	la-3
psekraac t4	st-g-gap	rt-11	ktu-1	la-1
psekraac t5	st-lambda-correlation	rt-15	ktu-2	la-3
psekraac t6A	st-g-gap	rt-20	ktu-1	la-3
psekraac t6B	st-g-gap	rt-5	ktu-3	la-3
psekraac t6C	st-g-gap	rt-5	ktu-2	la-3
psekraac t7	st-lambda-correlation	rt-10	ktu-1	la-2
psekraac t8	st-lambda-correlation	rt-11	ktu-1	la-3
psekraac t9	st-g-gap	rt-12	ktu-2	la-3
ta				
tpc				
waac	aaindex	AURR980118; WOLS870102; RICJ880104; KUMS000103; AURR980115; QIAN880117; FASG760103; GEOR030103; FINA910104; KHAG800101; GEOR030106; RACS820102; QIAN880103; QIAN880101; BUNA790103; BUNA790102;		

encoding	params_1	params_2	params_3	params_4
		QIAN880102; VASM830101; ROBB760111; ZIMJ680104; RACS820107		
zscale				

Statistics

anova_summary_aov

	term	df	sumsq	meansq	statistic	p.value	experiment
**1**	model	3	19.329082	6.443027	1489.093637	0.0	anova_summary_aov
**2**	Residuals	396	1.713417	0.004327	-	-	anova_summary_aov

anova_tukey_hsd

	term	contrast	null.value	estimate	conf.low	conf.high	adj.p.value	experiment
**1**	model	dt-bayes	0	-0.366387	-0.390387	-0.342387	0	anova_tukey_hsd
**2**	model	lr-bayes	0	-0.586519	-0.610519	-0.562518	0	anova_tukey_hsd
**3**	model	rf-bayes	0	-0.471361	-0.495361	-0.447361	0	anova_tukey_hsd
**4**	model	lr-dt	0	-0.220131	-0.244132	-0.196131	0	anova_tukey_hsd
**5**	model	rf-dt	0	-0.104974	-0.128974	-0.080973	0	anova_tukey_hsd
**6**	model	rf-lr	0	0.115158	0.091158	0.139158	0	anova_tukey_hsd

anova_error_summary_aov

	term	df	sumsq	meansq	statistic	p.value	experiment
**1**	model	4	428.419104	107.104776	57211.583267	0.0	anova_error_summary_aov
**2**	Residuals	500787	937.514335	0.001872	-	-	anova_error_summary_aov

anova_error_tukey_hsd

	term	contrast	null.value	estimate	conf.low	conf.high	adj.p.value	experiment
**1**	model	dt-bayes	0	-0.009988	-0.010515	-0.009461	0	anova_error_tukey_hsd
**2**	model	lr-bayes	0	-0.046650	-0.047178	-0.046123	0	anova_error_tukey_hsd
**3**	model	mlp-bayes	0	-0.053125	-0.053653	-0.052598	0	anova_error_tukey_hsd
**4**	model	rf-bayes	0	-0.079785	-0.080312	-0.079257	0	anova_error_tukey_hsd
**5**	model	lr-dt	0	-0.036662	-0.037190	-0.036135	0	anova_error_tukey_hsd
**6**	model	mlp-dt	0	-0.043137	-0.043664	-0.042610	0	anova_error_tukey_hsd
**7**	model	rf-dt	0	-0.069797	-0.070324	-0.069269	0	anova_error_tuke

	term	contrast	null.value	estimate	conf.low	conf.high	adj.p.value	experiment
								y_hsd
8	model	mlp-lr	0	-0.006475	-0.007002	-0.005947	0	anova_error_tukey_hsd
9	model	rf-lr	0	-0.033134	-0.033662	-0.032607	0	anova_error_tukey_hsd
10	model	rf-mlp	0	-0.026660	-0.027187	-0.026132	0	anova_error_tukey_hsd

anova_kappa_summary_aov

	term	df	sumsq	meansq	statistic	p.value	experiment
1	model	4	1999.050157	499.762539	16557.087179	0.0	anova_kappa_summary_aov
2	Residuals	500787	15115.858249	0.030184	-	-	anova_kappa_summary_aov

anova_kappa_tukey_hsd

	term	contrast	null.value	estimate	conf.low	conf.high	adj.p.value	experiment
1	model	dt-bayes	0	0.000152	-0.001966	0.002270	0.999672	anova_kappa_tukey_hsd
2	model	lr-bayes	0	-0.021451	-0.023569	-0.019333	0.000000	anova_kappa_tukey_hsd
3	model	mlp-bayes	0	0.009586	0.007468	0.011703	0.000000	anova_kappa_tukey_hsd
4	model	rf-bayes	0	0.152979	0.150861	0.155097	0.000000	anova_kappa_tukey_hsd
5	model	lr-dt	0	-0.021603	-0.023721	-0.019486	0.000000	anova_kappa_tukey_hsd
6	model	mlp-dt	0	0.009434	0.007316	0.011551	0.000000	anova_kappa_tukey_hsd
7	model	rf-dt	0	0.152827	0.150710	0.154945	0.000000	anova_kappa_tukey_hsd
8	model	mlp-lr	0	0.031037	0.028920	0.033155	0.000000	anova_kappa_tukey_hsd
9	model	rf-lr	0	0.174431	0.172313	0.176549	0.000000	anova_kappa_tukey_hsd
10	model	rf-mlp	0	0.143394	0.141276	0.145511	0.000000	anova_kappa_tukey_hsd

manova_summary

	term	df	pillai	statistic	num.df	den.df	p.value	experiment
1	model	4	0.334092	26498.942255	8.0	1057068.0	0.0	manova_summary
2	Residuals	528534	-	-	-	-	-	manova_summary

manova_summary_aov

	Df	Sum.Sq	Mean.Sq	F.value	Pr..F.	response	experiment
**model**	4	2050.807974	512.701993	14445.933073	0.0	Response 1	manova_summary_aov
**Residuals**	528534	18758.250784	0.035491	-	-	Response 1	manova_summary_aov
**model 1**	4	445.427157	111.356789	49705.434981	0.0	Response 2	manova_summary_aov
**Residuals 1**	528534	1184.092830	0.002240	-	-	Response 2	manova_summary_aov

Plots

Refer to main manuscript for more details.

Suppl. Fig. 1. MVO fitness vs. generations.

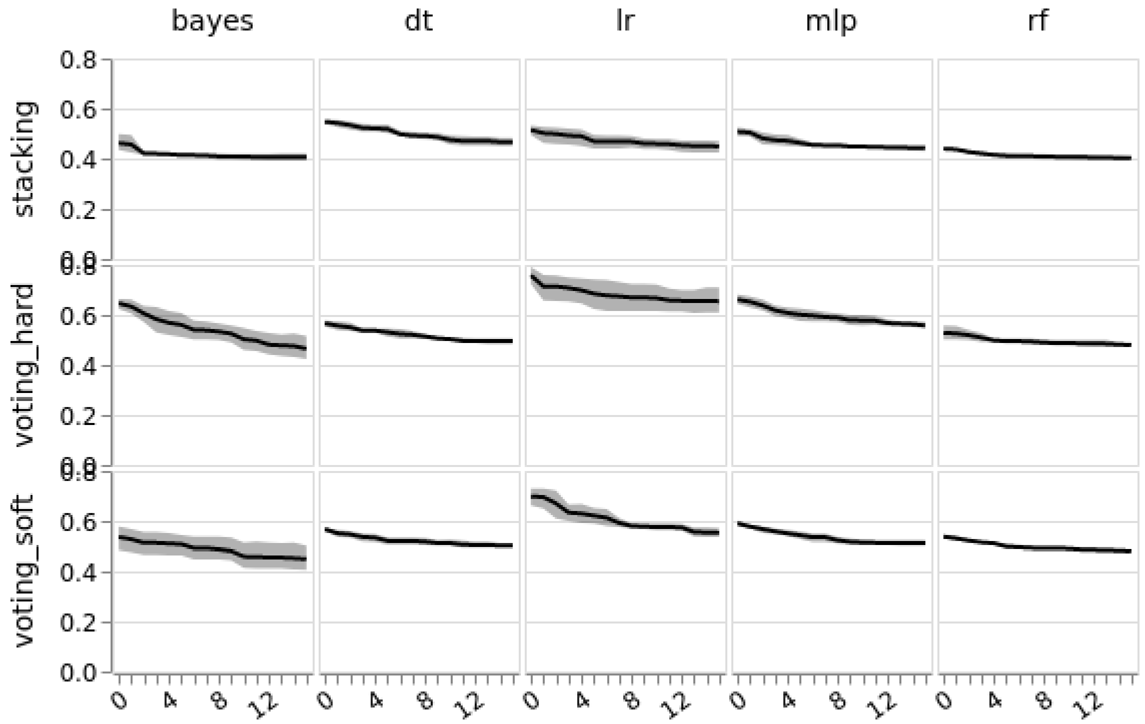

Suppl. Fig. 2. XCD chart

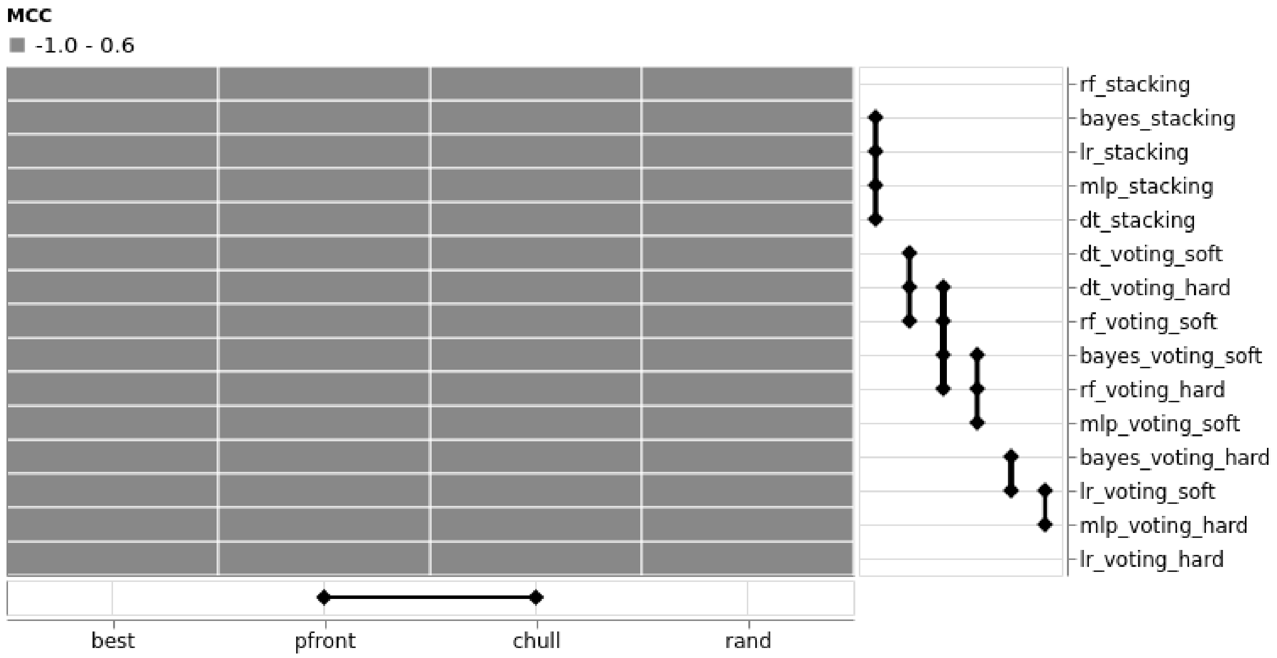

Suppl. Fig. 3. Boxplot

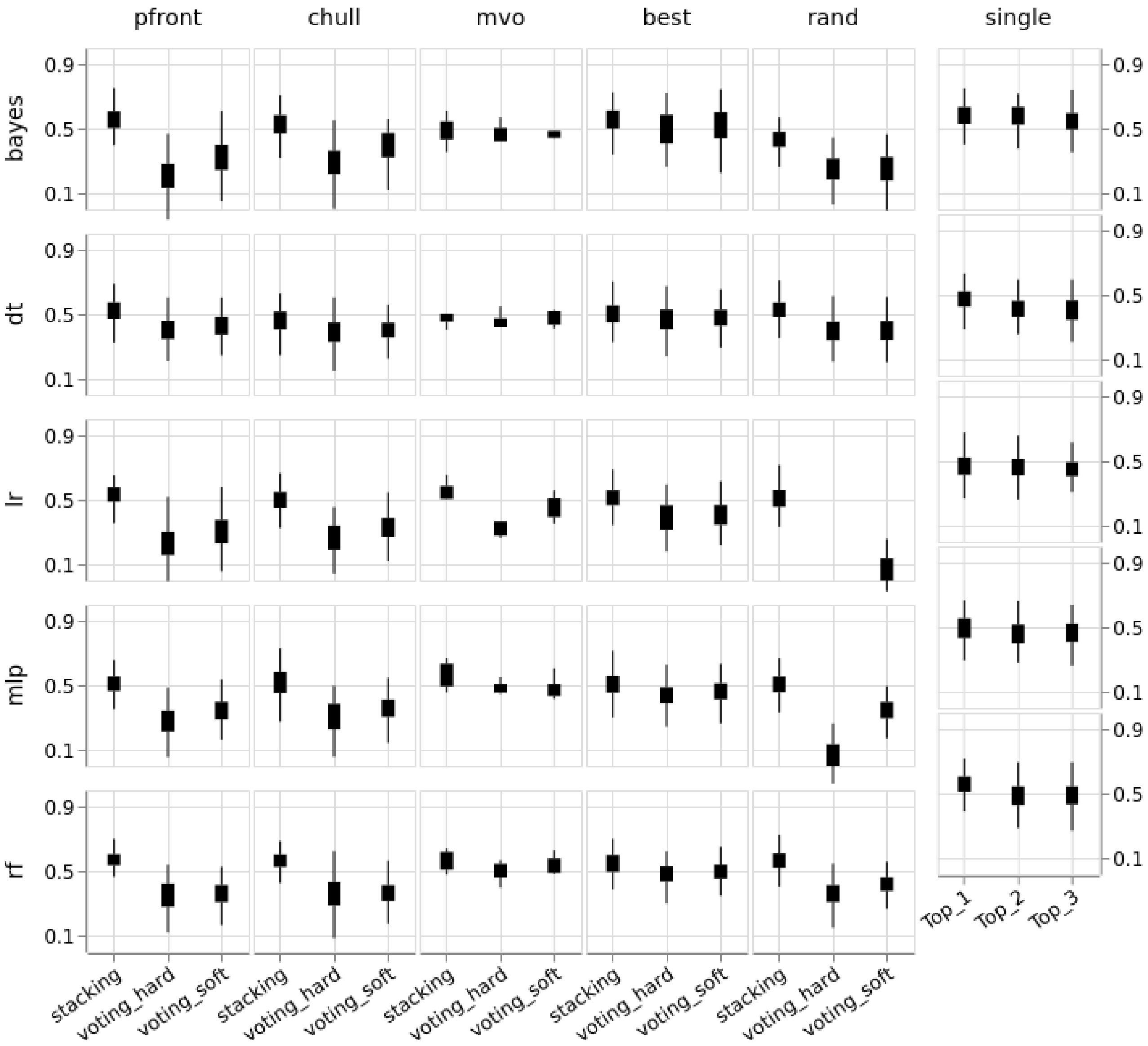

Suppl. Fig. 4. Kappa-error plot

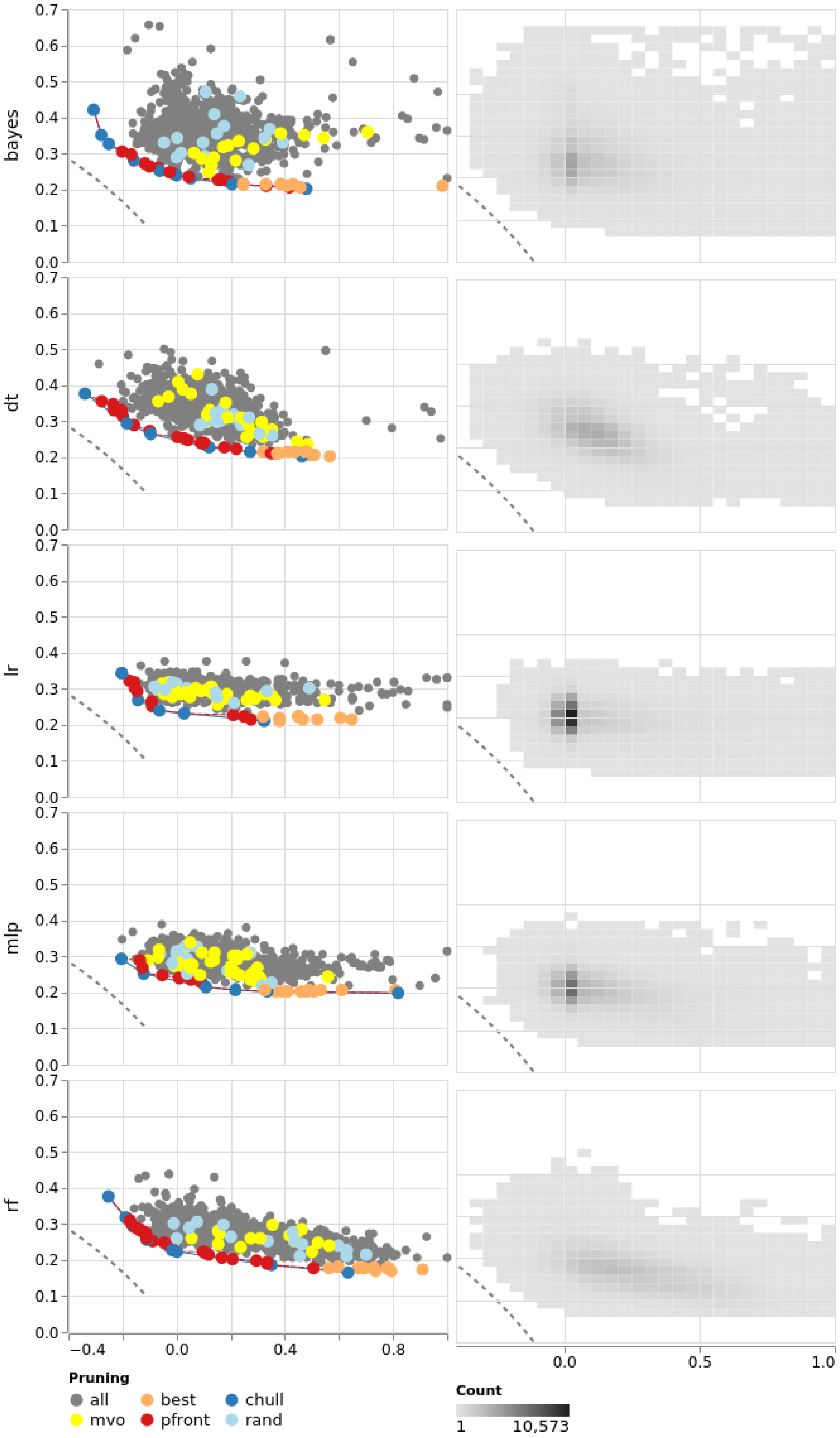
bayes
dt
lr
mlp
rf
0.0
0.1
0.2
0.3
0.4
0.5
0.6
0.7
−0.4
0.0
0.4
0.8
0.0
0.5
1.0
Pruning
all
best
chull
mvo
pfront
rand
Count
1
10,573

Suppl. Fig. 5. Boxplot MANOVA

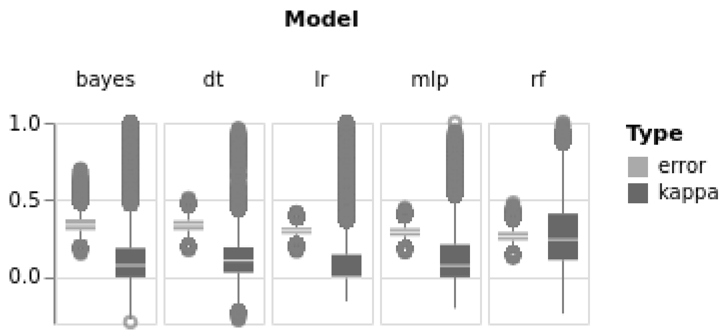

Supplement: Supplementary file 1 — Additional file 1. [file 13040_2022_317_MOESM1_ESM.zip › supplements/isp_il10predR1.pdf]
