# Supplementary material for: Unsupervised encoding selection through ensemble pruning for biomedical classification
Source: BioData Min. 2023 Mar 16;16:10. doi: 10.1186/s13040-022-00317-7 (PMC10018861; doi:10.1186/s13040-022-00317-7)

List of encodings

Refer to Spänig *et al.* (2021) for more details (https://doi.org/10.1093/nargab/lqab039).

encoding	params_1	params_2	params_3	params_4
aac				
aaindex	AURR980118; FASG760103; RACS820102; GEOR030103; FINA910104; QIAN880103; WOLS870102; KUMS000103; VASM830101; RACS820107; QIAN880101; AURR980115; RICJ880104; GEOR030106; ZIMJ680104; QIAN880102; QIAN880117; ROBB760111; KHAG800101; BUNA790103; BUNA790102			
apaac	lambda	1; 2; 3		
asa				
binary				
blomap				
blosum62				
cgr	res	20; 100; 10; 200	sf	0.5; 0.8632713
cksaagp	gap	1; 2		
cksaap	gap	1; 2		
ctdc				
ctdd				
ctdt				

encoding	params_1	params_2	params_3	params_4
ctriad				
dde				
delaunay	frequency; number; total; average; cartesian	product; distance; instances		
disorderb				
disorderc				
dist_freq	dn	100; 10; 5; 50; 20	dc	100; 10; 5; 50; 20
distance	distribution			
dpc				
eaac	window	1; 2; 3		
egaac	window	4; 2; 6; 5; 3; 8; 7; 1		
electrostatic_hull		6; 0; 3; 9; 12		
fft	aaindex	AURR980118; FASG760103; RACS820102; GEOR030103; FINA910104; QIAN880103; WOLS870102; KUMS000103; VASM830101; RACS820107; QIAN880101; AURR980115; RICJ880104; GEOR030106; ZIMJ680104; QIAN880102; QIAN880117; ROBB760111; KHAG800101; BUNA790103; BUNA790102		

encoding	params_1	params_2	params_3	params_4
fldpc	aaindex	AURR980118; FASG760103; RACS820102; GEOR030103; FINA910104; QIAN880103; WOLS870102; KUMS000103; VASM830101; RACS820107; QIAN880101; AURR980115; RICJ880104; GEOR030106; ZIMJ680104; QIAN880102; QIAN880117; ROBB760111; KHAG800101; BUNA790103; BUNA790102		
flgc	aaindex	AURR980118; FASG760103; RACS820102; GEOR030103; FINA910104; QIAN880103; WOLS870102; KUMS000103; VASM830101; RACS820107; QIAN880101; AURR980115; RICJ880104; GEOR030106; ZIMJ680104; QIAN880102; QIAN880117; ROBB760111; KHAG800101; BUNA790103; BUNA790102		

encoding	params_1	params_2	params_3	params_4
gaac				
gdpc				
geary	nlag	1; 2; 3		
gtpc				
moran	nlag	1; 2; 3		
ngram	e2; a3; e3; s2; a2; s3	100; 20; 200; 5; 50; 300; 1		
nmbroto	nlag	1; 2; 3		
paac	lambda	1; 2; 3		
qsar				
qsorder	nlag	1; 2; 3		
socnumber	nlag	1; 2; 3		
sseb				
ssec				
psekraac t1	st-lambda-correlation	rt-7	ktu-3	la-6
psekraac t10	st-g-gap	rt-9	ktu-1	la-1
psekraac t11	st-lambda-correlation	rt-7	ktu-3	la-3
psekraac t12	st-lambda-correlation	rt-8	ktu-1	la-1
psekraac t13	st-lambda-correlation	rt-12	ktu-1	la-3
psekraac t14	st-g-gap	rt-18	ktu-3	la-3
psekraac t15	st-lambda-correlation	rt-15	ktu-1	la-2
psekraac t16	st-g-gap	rt-10	ktu-1	la-1
psekraac t2	st-lambda-correlation	rt-2	ktu-1	la-1
psekraac t3A	st-lambda-correlation	rt-15	ktu-3	la-3
psekraac t3B	st-g-gap	rt-2	ktu-1	la-3

encoding	params_1	params_2	params_3	params_4
psekraac t4	st-g-gap	rt-9	ktu-1	la-1
psekraac t5	st-g-gap	rt-10	ktu-1	la-3
psekraac t6A	st-lambda-correlation	rt-4	ktu-1	la-2
psekraac t6B	st-g-gap	rt-5	ktu-1	la-3
psekraac t6C	st-g-gap	rt-5	ktu-1	la-1
psekraac t7	st-lambda-correlation	rt-10	ktu-1	la-3
psekraac t8	st-g-gap	rt-18	ktu-3	la-3
psekraac t9	st-lambda-correlation	rt-6	ktu-2	la-2
ta				
tpc				
waac	aaindex	AURR980118; FASG760103; RACS820102; GEOR030103; FINA910104; QIAN880103; WOLS870102; KUMS000103; VASM830101; RACS820107; QIAN880101; AURR980115; RICJ880104; GEOR030106; ZIMJ680104; QIAN880102; QIAN880117; ROBB760111; KHAG800101; BUNA790103; BUNA790102		
zscale				

Statistics

anova_summary_aov

	term	df	sumsq	meansq	statistic	p.value	experiment
**1**	model	3	19.853033	6.617678	202.888456	0.0	anova_summary_aov
**2**	Residuals	396	12.916459	0.032617	-	-	anova_summary_aov

anova_tukey_hsd

	term	contrast	null.value	estimate	conf.low	conf.high	adj.p.value	experiment
**1**	model	dt-bayes	0	-0.474202	-0.540098	-0.408307	0.000000	anova_tukey_hsd
**2**	model	lr-bayes	0	-0.028331	-0.094226	0.037564	0.684056	anova_tukey_hsd
**3**	model	rf-bayes	0	-0.443288	-0.509183	-0.377393	0.000000	anova_tukey_hsd
**4**	model	lr-dt	0	0.445871	0.379976	0.511767	0.000000	anova_tukey_hsd
**5**	model	rf-dt	0	0.030914	-0.034981	0.096810	0.620615	anova_tukey_hsd
**6**	model	rf-lr	0	-0.414957	-0.480852	-0.349062	0.000000	anova_tukey_hsd

anova_error_summary_aov

	term	df	sumsq	meansq	statistic	p.value	experiment
**1**	model	4	548.672122	137.168030	28063.033911	0.0	anova_error_summary_aov
**2**	Residuals	500991	2448.771182	0.004888	-	-	anova_error_summary_aov

anova_error_tukey_hsd

	term	contrast	null.value	estimate	conf.low	conf.high	adj.p.value	experiment
**1**	model	dt-bayes	0	-0.029547	-0.030399	-0.028695	0	anova_error_tukey_hsd
**2**	model	lr-bayes	0	-0.045355	-0.046207	-0.044503	0	anova_error_tukey_hsd
**3**	model	mlp-bayes	0	-0.067864	-0.068716	-0.067012	0	anova_error_tukey_hsd
**4**	model	rf-bayes	0	-0.097283	-0.098135	-0.096431	0	anova_error_tukey_hsd
**5**	model	lr-dt	0	-0.015807	-0.016659	-0.014955	0	anova_error_tukey_hsd
**6**	model	mlp-dt	0	-0.038316	-0.039168	-0.037464	0	anova_error_tukey_hsd
**7**	model	rf-dt	0	-0.067735	-0.068587	-0.066884	0	anova_error_tukey_hsd
**8**	model	mlp-lr	0	-0.022509	-0.023361	-0.021657	0	anova_error_tukey_hsd
**9**	model	rf-lr	0	-0.051928	-0.052780	-0.051076	0	anova_error_tukey_hsd
**10**	model	rf-mlp	0	-0.029419	-0.030271	-0.028567	0	anova_error_tukey_hsd

anova_kappa_summary_aov

	term	df	sumsq	meansq	statistic	p.value	experiment
**1**	model	4	2686.214025	671.553506	16243.301179	0.0	anova_kappa_summary_aov
**2**	Residuals	500991	20712.677733	0.041343	-	-	anova_kappa_summary_aov

anova_kappa_tukey_hsd

	term	contrast	null.value	estimate	conf.low	conf.high	adj.p.value	experiment
**1**	model	dt-bayes	0	0.030726	0.028248	0.033203	0	anova_kappa_tukey_hsd
**2**	model	lr-bayes	0	0.098667	0.096188	0.101145	0	anova_kappa_tukey_hsd
**3**	model	mlp-bayes	0	0.132210	0.129732	0.134688	0	anova_kappa_tukey_hsd
**4**	model	rf-bayes	0	0.205730	0.203252	0.208207	0	anova_kappa_tukey_hsd
**5**	model	lr-dt	0	0.067941	0.065463	0.070419	0	anova_kappa_tukey_hsd
**6**	model	mlp-dt	0	0.101485	0.099007	0.103962	0	anova_kappa_tukey_hsd
**7**	model	rf-dt	0	0.175004	0.172526	0.177481	0	anova_kappa_tukey_hsd
**8**	model	mlp-lr	0	0.033544	0.031065	0.036022	0	anova_kappa_tukey_hsd
**9**	model	rf-lr	0	0.107063	0.104585	0.109541	0	anova_kappa_tukey_hsd
**10**	model	rf-mlp	0	0.073519	0.071041	0.075997	0	anova_kappa_tukey_hsd

manova_summary

	term	df	pillai	statistic	num.df	den.df	p.value	experiment
**1**	model	4	0.172484	12704.606346	8.0	1076868.0	0.0	manova_summary
**2**	Residuals	538434	-	-	-	-	-	manova_summary

manova_summary_aov

	Df	Sum.Sq	Mean.Sq	F.value	Pr..F.	response	experiment
**model**	4	2840.368312	710.092078	14878.97939	0.0	Response 1	manova_summary_aov
**Residuals**	538434	25696.501612	0.047725	-	-	Response 1	manova_summary_aov
**model 1**	4	582.852371	145.713093	26543.696569	0.0	Response 2	manova_summary_aov
**Residuals 1**	538434	2955.763272	0.005490	-	-	Response 2	manova_summary_aov

Plots

Refer to main manuscript for more details.

Suppl. Fig. 1. MVO fitness vs. generations.

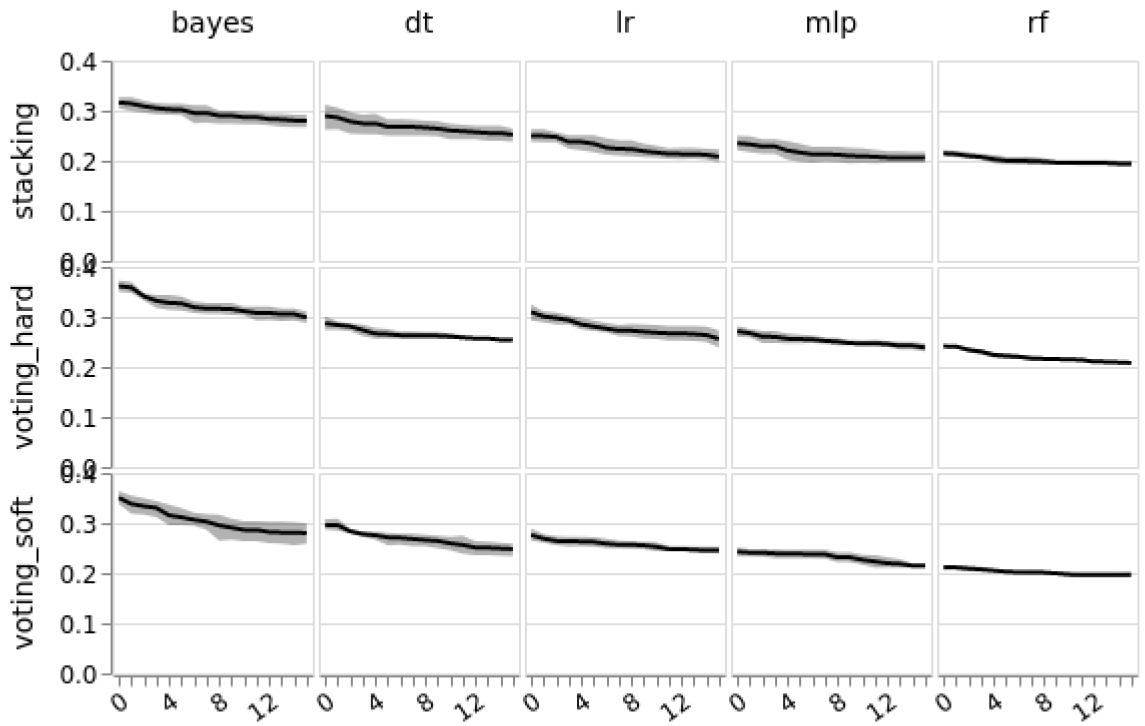

Suppl. Fig. 2. XCD chart

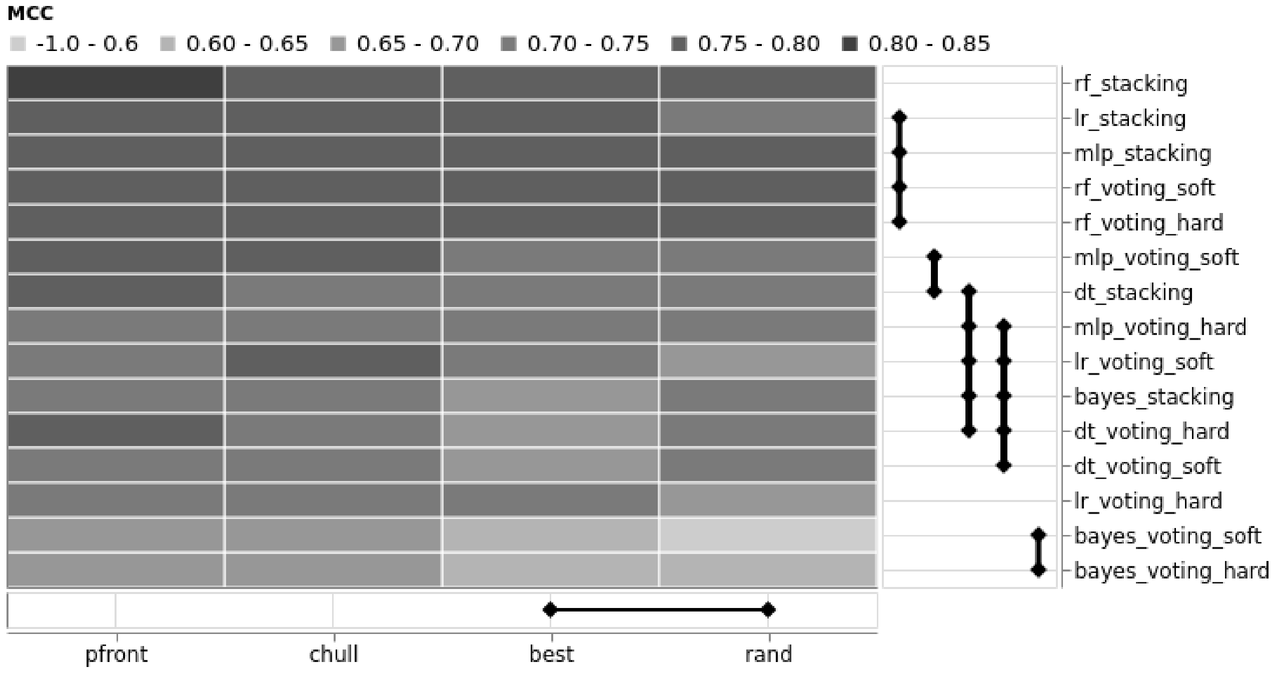

Suppl. Fig. 3. Boxplot

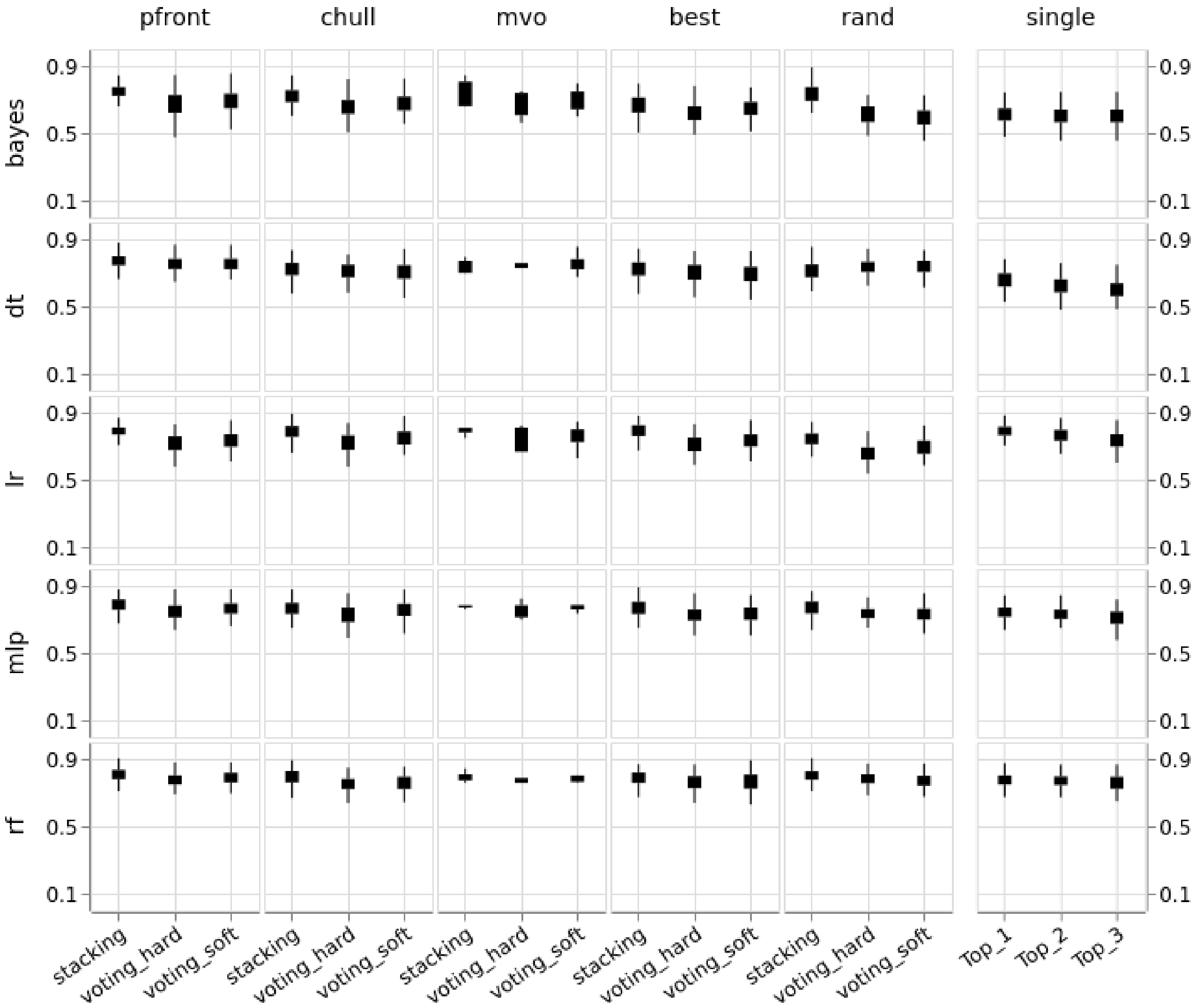

Suppl. Fig. 4. Kappa-error plot

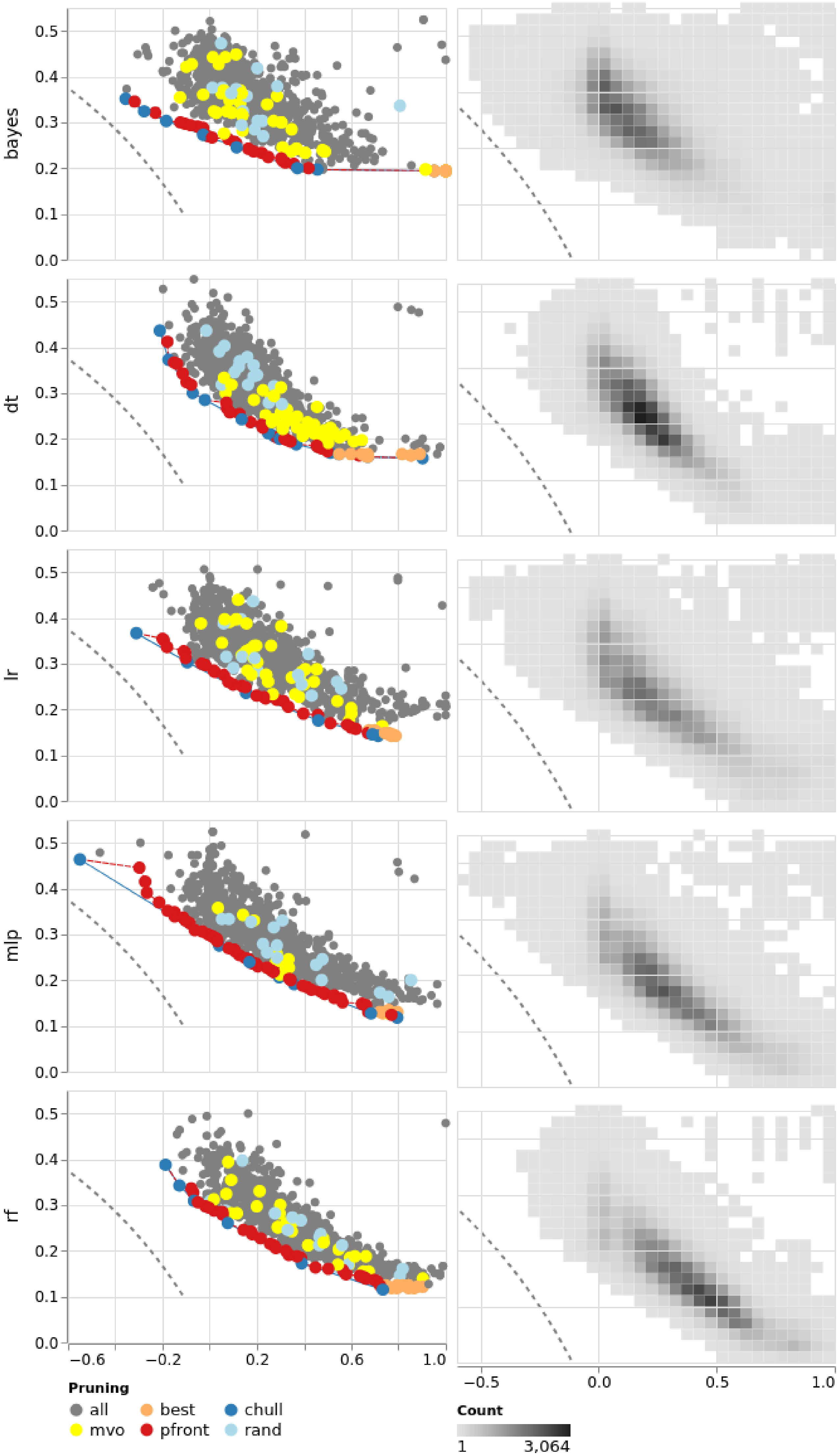

bayes
dt
lr
mlp
rf
0.0
0.1
0.2
0.3
0.4
0.5
−0.6
−0.2
0.2
0.6
1.0
−0.5
0.0
0.5
1.0
Pruning
all
best
chull
mvo
pfront
rand
Count
1
3,064

Suppl. Fig. 5. Boxplot MANOVA

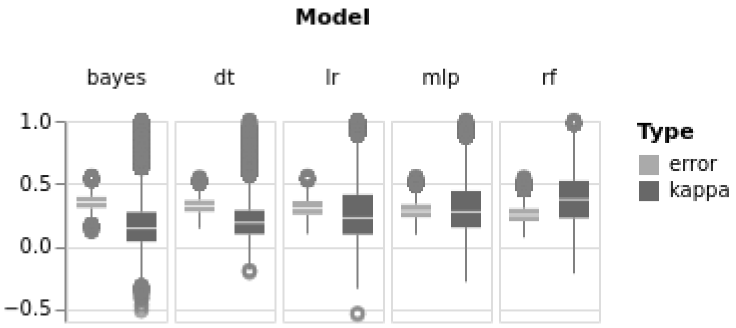

Supplement: Supplementary file 1 — Additional file 1. [file 13040_2022_317_MOESM1_ESM.zip › supplements/nep_neuropipredR1.pdf]
