# Supplementary material for: Unsupervised encoding selection through ensemble pruning for biomedical classification
Source: BioData Min. 2023 Mar 16;16:10. doi: 10.1186/s13040-022-00317-7 (PMC10018861; doi:10.1186/s13040-022-00317-7)

List of encodings

Refer to Spänig *et al.* (2021) for more details (https://doi.org/10.1093/nargab/lqab039).

encoding	params_1	params_2	params_3	params_4
aac				
aaindex	GEOR030103; RICJ880104; GEOR030106; ROBB760111; AURR980115; FASG760103; KUMS000103; QIAN880117; KHAG800101; ZIMJ680104; RACS820107; BUNA790102; AURR980118; QIAN880101; VASM830101; QIAN880103; BUNA790103; WOLS870102; FINA910104; QIAN880102; RACS820102			
apaac	lambda	9; 10; 5; 7; 3; 2; 4; 1; 6; 8		
asa				
binary				
blomap				
blosum62				
cgr	res	20; 10; 200; 100	sf	0.8632713; 0.5
cksaagp	gap	9; 5; 7; 3; 2; 4; 1; 6; 8		
cksaap	gap	9; 5; 7; 3; 2; 4; 1; 6; 8		
ctdc				

**encoding**	**params_1**	**params_2**	**params_3**	**params_4**
ctdd				
ctdt				
ctriad				
dde				
delaunay	frequency; number; cartesian; total; average	instances; distance; product		
disorderb				
disorderc				
dist_freq	dn	10; 5; 20; 50; 100	dc	10; 5; 20; 50; 100
distance	distribution			
dpc				
eaac	window	9; 10; 5; 7; 3; 2; 4; 1; 6; 8		
egaac	window	5; 7; 3; 2; 4; 1; 6; 8		
electrostatic_hull		9; 3; 0; 6; 12		
fft	aaindex	GEOR030103; RICJ880104; GEOR030106; ROBB760111; AURR980115; FASG760103; KUMS000103; QIAN880117; KHAG800101; ZIMJ680104; RACS820107; BUNA790102; AURR980118; QIAN880101; VASM830101; QIAN880103; BUNA790103; WOLS870102;		

encoding	params_1	params_2	params_3	params_4
		FINA910104; QIAN880102; RACS820102		
fldpc	aaindex	GEOR030103; RICJ880104; GEOR030106; ROBB760111; AURR980115; FASG760103; KUMS000103; QIAN880117; KHAG800101; ZIMJ680104; RACS820107; BUNA790102; AURR980118; QIAN880101; VASM830101; QIAN880103; BUNA790103; WOLS870102; FINA910104; QIAN880102; RACS820102		
flgc	aaindex	GEOR030103; RICJ880104; GEOR030106; ROBB760111; AURR980115; FASG760103; KUMS000103; QIAN880117; KHAG800101; ZIMJ680104; RACS820107; BUNA790102; AURR980118; QIAN880101; VASM830101; QIAN880103; BUNA790103; WOLS870102; FINA910104;		

encoding	params_1	params_2	params_3	params_4
		QIAN880102; RACS820102		
gaac				
gdpc				
geary	nlag	9; 10; 5; 7; 3; 2; 4; 1; 6; 8		
gtpc				
ksctriad	gap	3; 4; 1; 2		
moran	nlag	9; 10; 5; 7; 3; 2; 4; 1; 6; 8		
ngram	a2; a3; e3; s3; e2; s2	5; 300; 20; 1; 50; 200; 100		
nmbroto	nlag	9; 10; 5; 7; 3; 2; 4; 1; 6; 8		
paac	lambda	9; 10; 5; 7; 3; 2; 4; 1; 6; 8		
qsar				
qsorder	nlag	9; 10; 5; 7; 3; 2; 4; 1; 6; 8		
socnumber	nlag	9; 10; 5; 7; 3; 2; 4; 1; 6; 8		
sseb				
ssec				
psekraac t1	st-g-gap	rt-7	ktu-1	la-2
psekraac t10	st-g-gap	rt-10	ktu-1	la-1
psekraac t11	st-g-gap	rt-9	ktu-1	la-1
psekraac t12	st-lambda-correlation	rt-8	ktu-1	la-3
psekraac t13	st-g-gap	rt-17	ktu-1	la-1
psekraac t14	st-g-gap	rt-10	ktu-1	la-3
psekraac t15	st-lambda-correlation	rt-15	ktu-1	la-3

encoding	params_1	params_2	params_3	params_4
psekraac t16	st-g-gap	rt-7	ktu-1	la-2
psekraac t2	st-g-gap	rt-8	ktu-1	la-2
psekraac t3A	st-g-gap	rt-13	ktu-2	la-2
psekraac t3B	st-g-gap	rt-9	ktu-2	la-3
psekraac t4	st-g-gap	rt-9	ktu-1	la-3
psekraac t5	st-lambda-correlation	rt-20	ktu-2	la-2
psekraac t6A	st-lambda-correlation	rt-20	ktu-3	la-2
psekraac t6B	st-lambda-correlation	rt-5	ktu-3	la-2
psekraac t6C	st-lambda-correlation	rt-5	ktu-1	la-1
psekraac t7	st-lambda-correlation	rt-10	ktu-2	la-3
psekraac t8	st-g-gap	rt-11	ktu-1	la-2
psekraac t9	st-g-gap	rt-11	ktu-1	la-3
ta				
tpc				
waac	aaindex	GEOR030103; RICJ880104; GEOR030106; ROBB760111; AURR980115; FASG760103; KUMS000103; QIAN880117; KHAG800101; ZIMJ680104; RACS820107; BUNA790102; AURR980118; QIAN880101; VASM830101; QIAN880103; BUNA790103; WOLS870102;		

encoding	params_1	params_2	params_3	params_4
		FINA910104; QIAN880102; RACS820102		
zscale				

Statistics

anova_summary_aov

	term	df	sumsq	meansq	statistic	p.value	experiment
**1**	model	3	56.188034	18.729345	6357.268167	0.0	anova_summary_aov
**2**	Residuals	396	1.166668	0.002946	-	-	anova_summary_aov

anova_tukey_hsd

	term	contrast	null.value	estimate	conf.low	conf.high	adj.p.value	experiment
**1**	model	dt-bayes	0	-0.848756	-0.868560	-0.828952	0.000000	anova_tukey_hsd
**2**	model	lr-bayes	0	-0.916143	-0.935947	-0.896339	0.000000	anova_tukey_hsd
**3**	model	rf-bayes	0	-0.820579	-0.840383	-0.800774	0.000000	anova_tukey_hsd
**4**	model	lr-dt	0	-0.067386	-0.087191	-0.047582	0.000000	anova_tukey_hsd
**5**	model	rf-dt	0	0.028178	0.008374	0.047982	0.001561	anova_tukey_hsd
**6**	model	rf-lr	0	0.095564	0.075760	0.115368	0.000000	anova_tukey_hsd

anova_error_summary_aov

	term	df	sumsq	meansq	statistic	p.value	experiment
**1**	model	4	884.478277	221.119569	104333.87362	0.0	anova_error_summary_aov
**2**	Residuals	500936	1061.656668	0.002119	-	-	anova_error_summary_aov

anova_error_tukey_hsd

	term	contrast	null.value	estimate	conf.low	conf.high	adj.p.value	experiment
**1**	model	dt-bayes	0	-0.014349	-0.014910	-0.013787	0	anova_error_tukey_hsd
**2**	model	lr-bayes	0	-0.090096	-0.090657	-0.089535	0	anova_error_tukey_hsd
**3**	model	mlp-bayes	0	-0.084370	-0.084932	-0.083809	0	anova_error_tukey_hsd
**4**	model	rf-bayes	0	-0.100817	-0.101378	-0.100256	0	anova_error_tukey_hsd
**5**	model	lr-dt	0	-0.075748	-0.076309	-0.075187	0	anova_error_tukey_hsd
**6**	model	mlp-dt	0	-0.070022	-0.070583	-0.069461	0	anova_error_tukey_hsd
**7**	model	rf-dt	0	-0.086469	-0.087030	-0.085908	0	anova_error_tukey_hsd

	term	contrast	null.value	estimate	conf.low	conf.high	adj.p.value	experiment
**8**	model	mlp-lr	0	0.005726	0.005165	0.006287	0	anova_error_tukey_hsd
**9**	model	rf-lr	0	-0.010721	-0.011282	-0.010160	0	anova_error_tukey_hsd
**10**	model	rf-mlp	0	-0.016447	-0.017008	-0.015886	0	anova_error_tukey_hsd

anova_kappa_summary_aov

	term	df	sumsq	meansq	statistic	p.value	experiment
**1**	model	4	497.845238	124.461310	5649.190122	0.0	anova_kappa_summary_aov
**2**	Residuals	500936	11036.475892	0.022032	-	-	anova_kappa_summary_aov

anova_kappa_tukey_hsd

	term	contrast	null.value	estimate	conf.low	conf.high	adj.p.value	experiment
**1**	model	dt-bayes	0	-0.007112	-0.008921	-0.005303	5.062617e-14	anova_kappa_tukey_hsd
**2**	model	lr-bayes	0	-0.011231	-0.013040	-0.009422	0.000000e+00	anova_kappa_tukey_hsd
**3**	model	mlp-bayes	0	0.003242	0.001433	0.005051	1.008830e-05	anova_kappa_tukey_hsd
**4**	model	rf-bayes	0	0.073995	0.072186	0.075804	0.000000e+00	anova_kappa_tukey_hsd
**5**	model	lr-dt	0	-0.004119	-0.005928	-0.002310	5.238911e-09	anova_kappa_tukey_hsd
**6**	model	mlp-dt	0	0.010354	0.008545	0.012163	0.000000e+00	anova_kappa_tukey_hsd
**7**	model	rf-dt	0	0.081107	0.079298	0.082916	0.000000e+00	anova_kappa_tukey_hsd
**8**	model	mlp-lr	0	0.014473	0.012664	0.016282	0.000000e+00	anova_kappa_tukey_hsd
**9**	model	rf-lr	0	0.085226	0.083417	0.087035	0.000000e+00	anova_kappa_tukey_hsd
**10**	model	rf-mlp	0	0.070753	0.068944	0.072562	0.000000e+00	anova_kappa_tukey_hsd

manova_summary

	term	df	pillai	statistic	num.df	den.df	p.value	experiment
**1**	model	4	0.446273	38040.732728	8.0	1059528.0	0.0	manova_summary
**2**	Residuals	529764	-	-	-	-	-	manova_summary

manova_summary_aov

	Df	Sum.Sq	Mean.Sq	F.value	Pr.. F.	response	experiment
**model**	4	521.056302	130.264076	4481.321737	0.0	Response	manova_summar

	Df	Sum.Sq	Mean.Sq	F.value	Pr..F.	response	experiment
						1	y_aov
**Residuals**	529764	15399.299973	0.029068	-	-	Response 1	manova_summar y_aov
**model 1**	4	931.543864	232.885966	95228.956695	0.0	Response 2	manova_summar y_aov
**Residuals 1**	529764	1295.557625	0.002446	-	-	Response 2	manova_summar y_aov

Plots

Refer to main manuscript for more details.

Suppl. Fig. 1. MVO fitness vs. generations.

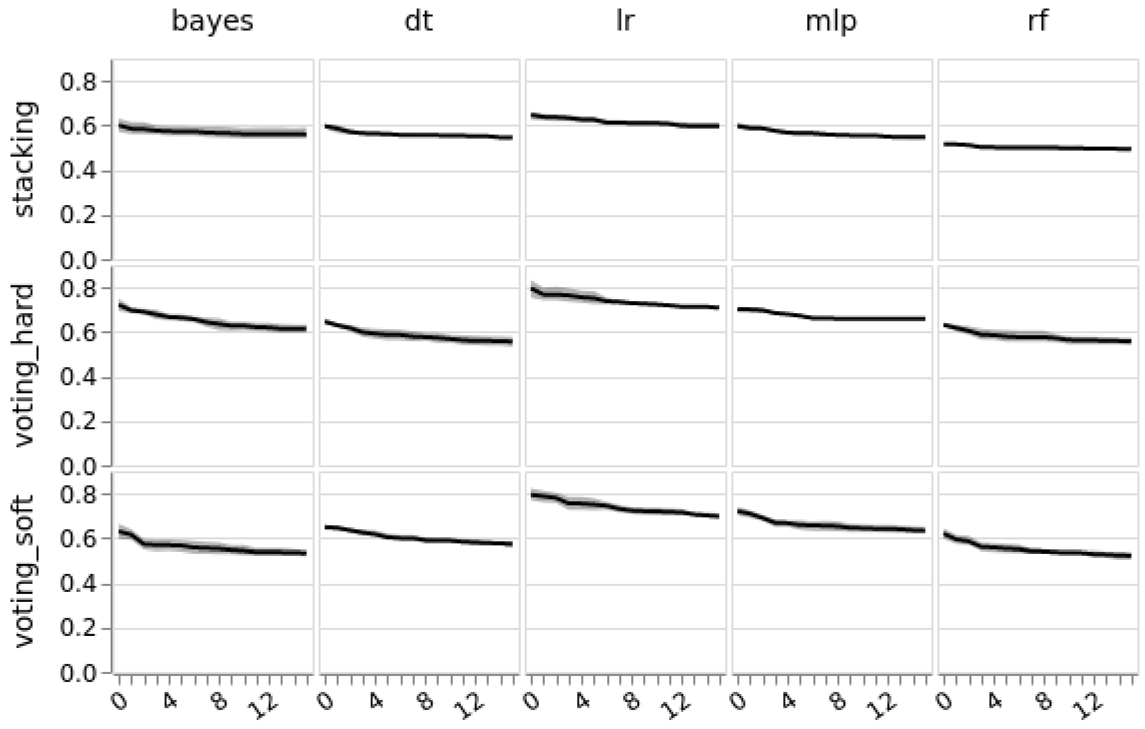

Suppl. Fig. 2. XCD chart

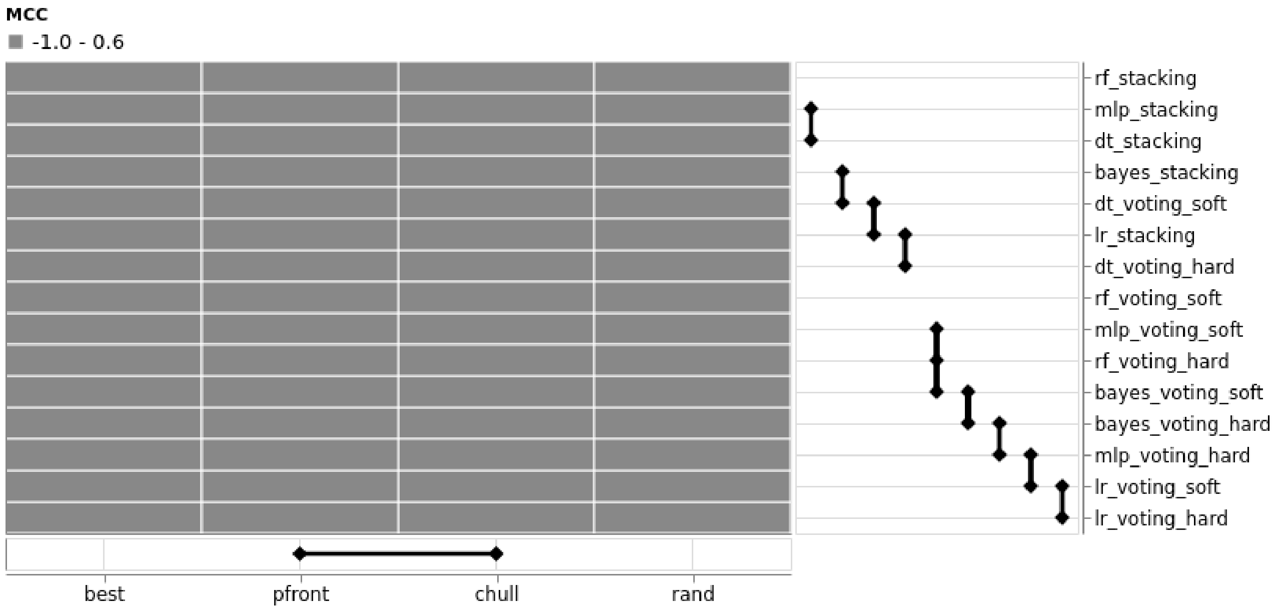

Suppl. Fig. 3. Boxplot

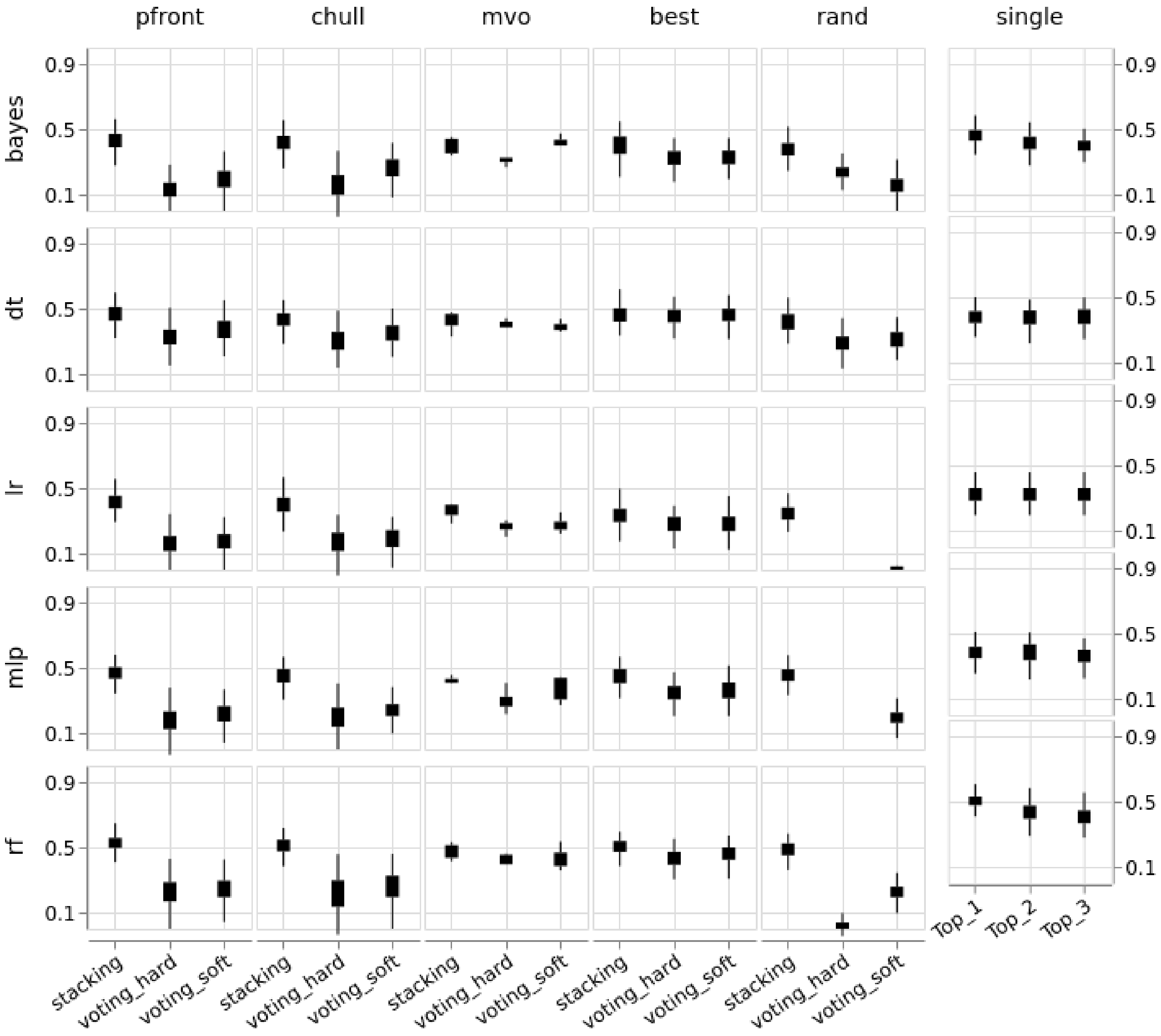

Suppl. Fig. 4. Kappa-error plot

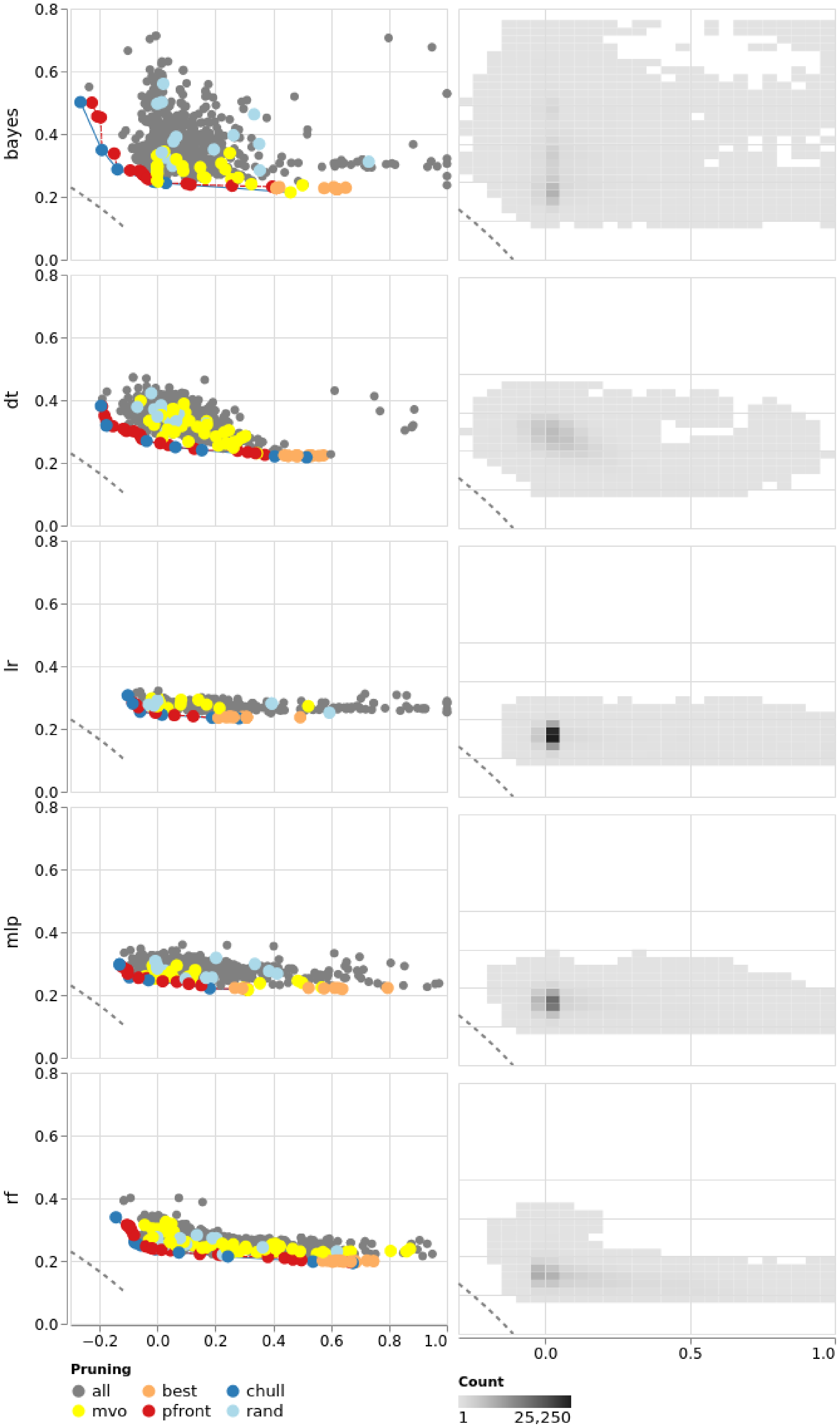

bayes
dt
lr
mlp
rf
Pruning
all
best
chull
mvo
pfront
rand
Count
1
25,250

Suppl. Fig. 5. Boxplot MANOVA

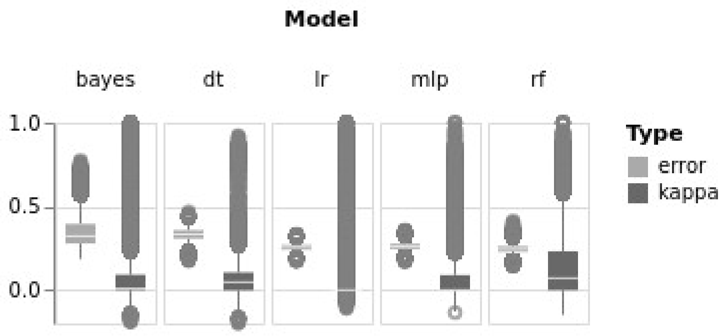

Supplement: Supplementary file 1 — Additional file 1. [file 13040_2022_317_MOESM1_ESM.zip › supplements/pip_pipelR1.pdf]
